# Apitegromab for lean mass preservation during tirzepatide-induced weight loss: a randomized, double-blind, placebo-controlled phase 2 trial

**DOI:** 10.1038/s41591-026-04440-4

**Published:** 2026-06-08

**Authors:** Richard E. Pratley, Douglas Scott Denham, Rupal Trivedi, Elaine Watkins, Lisa Connery, Jennifer Barnes, Dongzi Yu, Janet Hong, Christopher Simard, Kimberly Umans, Lan Liu, Giridhar S. Tirucherai, Jing L. Marantz

**Affiliations:** 1 AdventHealth Translational Research Institute, Orlando, FL USA; 2 Flourish Research - San Antonio, San Antonio, TX USA; 3 Flourish Research - Chicago, Chicago, IL USA; 4 ProSciento, Inc., Chula Vista, CA USA; 5 AMR Clinical, Norman, OK USA; 6 Scholar Rock, Inc., Cambridge, MA USA

**Keywords:** Phase II trials, Outcomes research

## Abstract

Loss of lean mass in proportion to total weight loss is observed with incretin mimetic therapies such as tirzepatide and has the potential to adversely affect health and function. Apitegromab is an investigational, fully human monoclonal antibody that selectively inhibits myostatin activation and is, thereby, capable of increasing muscle mass. In the randomized, double-blind, placebo-controlled phase 2 EMBRAZE study, adults with overweight or obesity (*n* = 102) were randomized 1:1 to receive tirzepatide plus apitegromab (10 mg kg^−1^) or tirzepatide plus placebo. At week 24, apitegromab resulted in a least square mean (80% confidence interval (CI)) of 1.9 (1.2−2.7) kg less lean mass loss than placebo (*P* = 0.001), despite similar total body weight loss between groups, representing a 54.9% retention of lean mass relative to placebo. In participants receiving apitegromab, trough concentrations of apitegromab and total latent myostatin, a pharmacodynamic marker, both increased over time and reached a plateau after approximately 16 weeks. Incidence of adverse events (AEs) (% (95% CI)) was generally similar across apitegromab-treated participants and placebo-treated participants, with 39 of 51 (76% (63−86%)) and 36 of 51 (71% (57−81%)) participants experiencing an AE, respectively. Serious adverse events (SAEs) were balanced and experienced by one of 51 (2% (0−10%)) participants in each arm. In summary, this proof-of-concept study demonstrated that selective targeting of myostatin by apitegromab was well tolerated and effective in preserving lean mass when combined with tirzepatide. ClinicalTrials.gov identifier: NCT06445075.

## Abstract (web summary)

In the phase 2 EMBRAZE study, participants receiving tirzepatide and apitegromab lost less lean mass compared to participants receiving tirzepatide and placebo.

## Main

The prevalence of overweight and obesity represents a global public health challenge^1^. Incretin mimetic therapies, including glucagon-like peptide 1 (GLP-1) receptor agonists and dual GLP-1/glucose-dependent insulinotropic polypeptide (GIP) receptor agonists, have substantially improved outcomes for patients, including significant weight loss and reduced risk of adverse cardiovascular events^2,3^. However, incretin mimetic therapy is accompanied by a loss of lean body mass that accounts for approximately 25−40% of total weight loss^4^. Lean body mass, primarily comprising skeletal muscle, is crucial for physical strength and overall health^5,6^. Higher lean body mass is associated with a higher metabolic rate^5^, improved insulin sensitivity and lower risk of type 2 diabetes^7,8^. Lean mass preservation is, therefore, an important consideration during pharmacologic weight management.

Myostatin is a member of the transforming growth factor beta (TGFβ) superfamily of growth factors that regulates catabolism of skeletal muscle^9–11^, making it an attractive target for muscle atrophy and for preserving muscle during pharmacologic weight loss^12,13^. Despite decades of attempts, targeting myostatin has failed to demonstrate clinical benefit until recently. Reasons for this include off-target side effects due to lack of target selectivity or inability to demonstrate efficacy due to inappropriate choice of disease or suboptimal trial design^14^.

Apitegromab is an investigational, fully human monoclonal antibody that selectively binds precursor forms of myostatin to potently inhibit myostatin activation, without activity toward other TGFβ superfamily members. In mouse models of muscle atrophy, apitegromab increased overall lean mass, muscle size and muscle force^11,15^. In phase 2 TOPAZ (NCT03921528) and phase 3 SAPPHIRE (NCT05156320) trials, apitegromab treatment improved motor function in patients with spinal muscular atrophy (SMA)^9,16^. Collectively, these studies suggest that selective targeting of myostatin has therapeutic potential in preserving muscle as part of pharmacologic weight management.

Here we report results from the randomized, double-blind, placebo-controlled, multicenter phase 2 EMBRAZE trial, which evaluated the efficacy and safety of apitegromab in adults with overweight or obesity receiving incretin mimetic therapy for pharmacologic weight management.

## Results

**Fig. 1:**
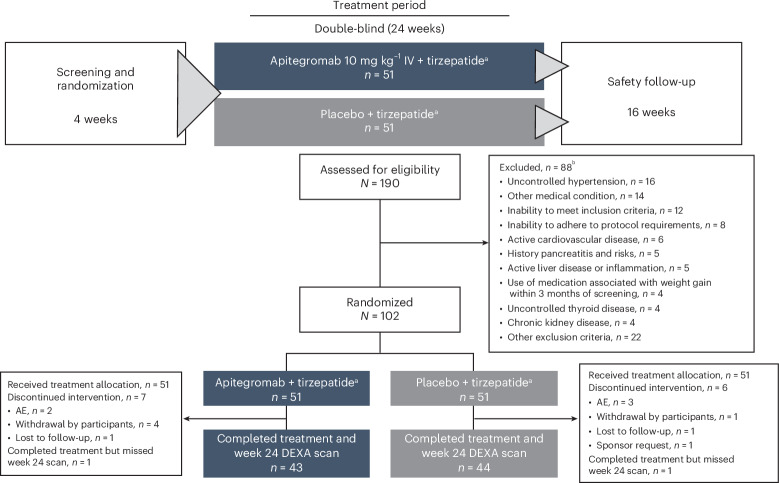
Trial design and participant disposition. ^a^Tirzepatide dose was increased by 2.5 mg every 4 weeks according to the recommended dose-escalation schedule and subject to treatment response and tolerability up to a maximum maintenance dose of 15 mg weekly. ^b^Individuals may have had more than one reason for exclusion. IV, intravenous.

From 18 June to 17 September 2024, 102 participants were enrolled and randomized to receive apitegromab 10 mg kg^−1^ every 4 weeks with tirzepatide (*n* = 51) or placebo every 4 weeks with tirzepatide (*n* = 51) (Fig. 1). Tirzepatide was titrated up to a maximum maintenance dose of 15 mg weekly according to the approved label. Efficacy analyses were based on the 43 participants receiving apitegromab and the 44 participants receiving placebo who completed treatment and had a week 24 dual-energy X-ray absorptiometry (DEXA) scan. Participant demographics and clinical characteristics were similar between treatment groups (Table 1). Most participants were women (apitegromab, 84.3% (43/51); placebo, 80.4% (41/51)), and mean age (s.d.) was similar (apitegromab, 44.2 years (11.0); placebo, 42.6 years (11.5)). At baseline, total body weight (s.d.) was 93.2 kg (14.9) and 98.8 kg (14.4), and body mass index (BMI) (s.d.) was 34.4 kg m^−^^2^ (4.1) and 36.4 kg m^−^^2^ (3.9) in the apitegromab and placebo groups, respectively.

**Table 1:** Participant baseline demographic and clinical characteristics

Characteristic	Apitegromab 10 mg kg^−1^ + tirzepatide (*n* = 51)	Placebo + tirzepatide (*n* = 51)
Female, *n* (%)	43 (84.3)	41 (80.4)
Age at screening, mean (s.d.) [min, max] (years)	44.2 (11.0) [21, 63]	42.6 (11.5) [18, 64]
Race, *n* (%)		
White	40 (78.4)	38 (74.5)
Black or African American	7 (13.7)	7 (13.7)
Other^a^	4 (7.8)	6 (11.8)
Weight, mean (s.d.) (kg)	93.2 (14.9)	98.8 (14.4)
BMI, mean (s.d.) (kg m^−^^2^)	34.4 (4.1)	36.4 (3.9)
≥30 kg m^−^^2^, *n* (%)	48 (94.1)	51 (100)
Lean body mass, mean (s.d.) (kg)	48.3 (9.9)	49.6 (8.1)
Body fat mass, mean (s.d.) (kg)	41.3 (8.8)	45.1 (9.2)
HgA1C (%)	5.40 (0.43)	5.43 (0.39)
Glucose, mean (s.d.) (mmol l^−1^)	5.24 (0.67)	5.13 (0.50)
Cholesterol, mean (s.d.) (mmol l^−1^)	5.01 (0.85)	5.02 (1.08)

^a^Includes American Indian or Alaska Native, Asian, multiple races and other (unspecified).

### Pharmacokinetics and pharmacodynamics

**Extended Data Fig. 1:**
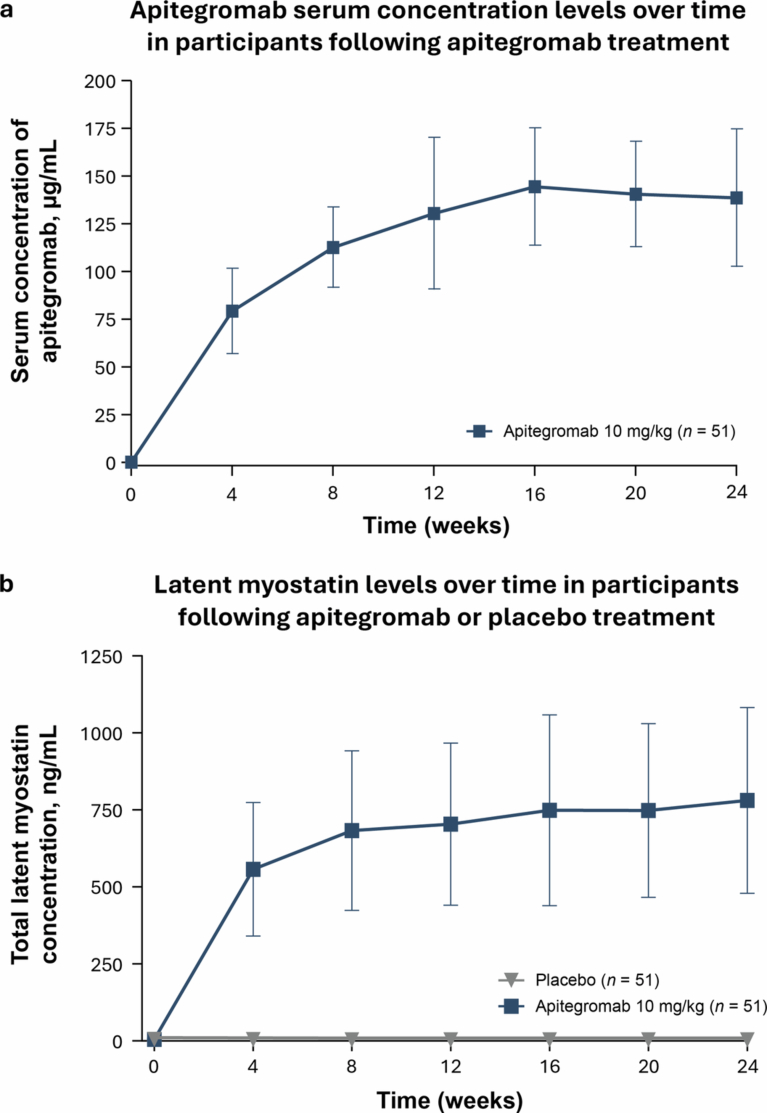
Serum trough concentration of apitegromab and total latent myostatin levels. **a**) Trough serum apitegromab concentration levels (PK) data are shown as geometric mean (CV%) µg/mL. **b**) Latent myostatin level (PD) data are shown as arithmetic mean (SD) ng/mL. PK samples from participants receiving placebo were not tested and therefore are not included in PK assessments. PD, pharmacodynamics; PK, pharmacokinetics; SD, standard deviation.

In participants receiving apitegromab, trough concentrations of apitegromab steadily increased over time, achieving steady-state levels by week 16 (Extended Data Fig. 1a). In these participants, concentrations of latent myostatin, a pharmacodynamic marker, increased substantially after the first dose, achieved a plateau at approximately week 16 and remained consistently elevated throughout the treatment period, demonstrating successful target engagement (Extended Data Fig. 1b). Overall, serum apitegromab concentrations varied over two-fold among study participants with minimal differences in total latent myostatin levels, indicating target saturation at 10 mg kg^−1^. Trough concentrations of tirzepatide were similar for both treatment groups (Extended Data Table 1).

### Efficacy

**Fig. 2:**
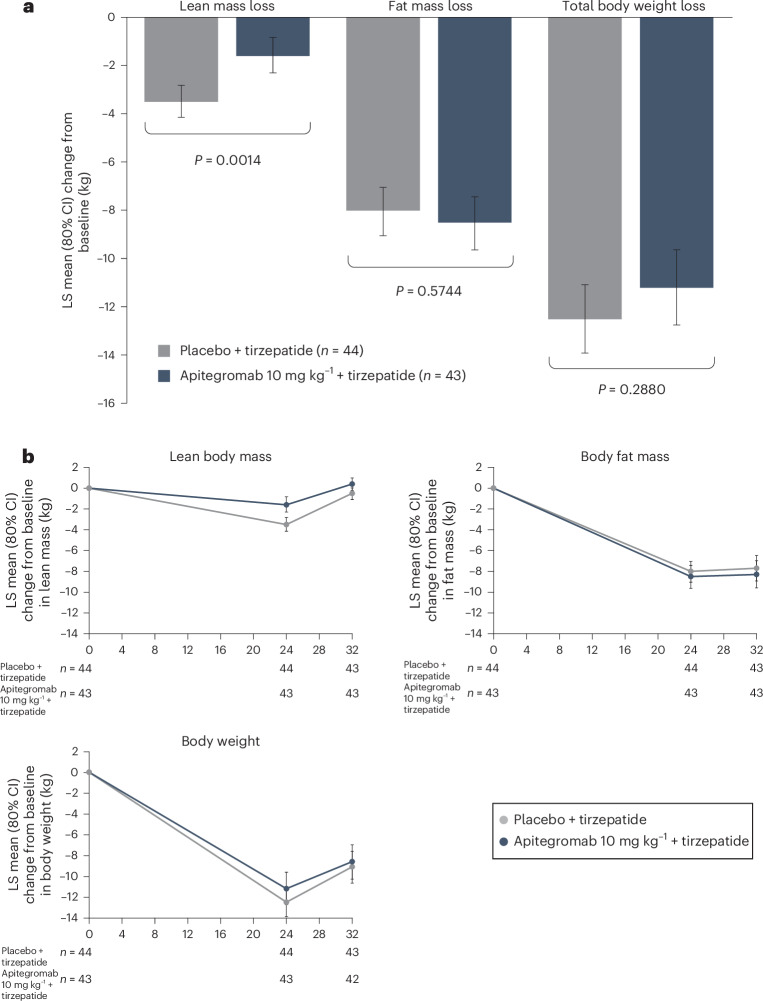
Lean body mass preservation with apitegromab versus placebo at week 24 and week 32. Data are presented as least square mean (80% CI), based on a linear regression model controlling for baseline weight, baseline lean body mass, age and sex. **a**, For the primary endpoint (week 24), apitegromab preserved an additional 1.9 kg of lean mass compared to placebo (nominal, *P* = 0.0014). Two-sided *P* values are based on the *t*-statistic, considered descriptive, and no adjustments were made for multiple comparisons. **b**, At week 32, the difference in lean mass between apitegromab and placebo remained significant (least square mean (80% CI), 0.9 (0.3−1.5); nominal, *P* = 0.0480). LS, least square.

At week 24, apitegromab treatment significantly reduced lean mass loss, preserving 1.9 kg (1.2−2.7, least square means (80% confidence interval (CI))) of lean mass compared to placebo (nominal, *P* = 0.0014). Lean mass loss was 1.6 kg (0.8−2.3) with apitegromab versus 3.5 kg (2.8−4.1) with placebo, a relative retention of 54.9% lean mass (Table 2 and Fig. 2a). Total body weight loss was similar between apitegromab and placebo (11.2 kg (9.6−12.7) versus 12.5 kg (11.1−13.9), respectively), with lean mass loss representing 14.6% (10.5−18.7%) of weight loss with apitegromab and 30.2% (26.4−33.9%) of weight loss with placebo. Correspondingly, fat mass loss comprised a larger proportion of total weight loss among those who received apitegromab (85.3% of total loss (81.2−89.5%) or 8.5 kg (7.4−9.6)) compared to those who received placebo (69.5% (65.7−73.3%) or 8.0 kg (7.0−9.0)).

**Table 2:** Efficacy summary at 24 weeks

	Apitegromab 10 mg kg^−1^ + tirzepatide (*n* = 43)	Placebo + tirzepatide (*n* = 44)	Difference apitegromab versus placebo	*P* value
Lean mass				
Change in lean mass (kg) (80% CI) (primary endpoint)	−1.6 (−2.3, −0.8)	−3.5 (−4.1, −2.8)	1.9 (1.2, 2.7)	0.0014
Percentage lean mass retention^a^			54.9%	
Change in lean mass as percent of total body mass (%) (80% CI)	4.2 (3.5, 4.9)	2.8 (2.2, 3.5)	1.4 (0.7, 2.1)	0.0122
Percentage of total loss due to lean mass loss (%) (80% CI)	14.6 (10.5, 18.7)	30.2 (26.4, 33.9)	−15.6 (−19.8, −11.4)	<0.0001
Fat mass				
Change in fat mass (kg) (80% CI)	−8.5 (−9.6, −7.4)	−8.0 (−9.0, −7.0)	−0.5 (−1.6, 0.6)	0.5744
Change in fat mass as percent of total body mass (%) (80% CI)	−4.6 (−5.3, −3.8)	−3.2 (−3.9, −2.5)	−1.4 (−2.1, −0.6)	0.0208
Percentage of total loss due to fat mass loss (%) (80% CI)	85.3 (81.2, 89.5)	69.5 (65.7, 73.3)	15.8 (11.6, 20.1)	<0.0001
Body weight				
Change in total body weight (kg) (80% CI)	−11.2 (−12.7, −9.6)	−12.5 (−13.9, −11.1)	1.3 (−0.3, 2.9)	0.2880
Visceral fat mass				
Change in visceral fat mass (kg) (80% CI)	−0.4 (−0.5, −0.3)	−0.4 (−0.4, −0.3)	0.0 (−0.1, 0.0)	0.5255
Change in percent of visceral fat mass in total adipose tissue (%) (80% CI)	−0.9 (−2.1, 0.3)	−1.0 (−2.1, 0.1)	0.1 (−1.1, 1.3)	0.8946
Subcutaneous fat mass				
Change in subcutaneous fat mass (kg) (80% CI)	−0.7 (−0.8, −0.6)	−0.7 (−0.8, −0.6)	0.0 (−0.1, 0.1)	0.8817
Change in percent of subcutaneous fat mass in total adipose tissue (%) (80% CI)	0.9 (−0.3, 2.1)	1.0 (−0.1, 2.1)	−0.1 (−1.3, 1.1)	0.8946
Trunk fat mass				
Change in trunk fat mass (kg) (80% CI)	−5.1 (−5.8, −4.5)	−4.9 (−5.5, −4.3)	−0.3 (−0.9, 0.4)	0.6362
Change in percent of trunk fat mass in total trunk mass (%) (80% CI)	−5.4 (−6.3, −4.5)	−4.1 (−4.9, −3.2)	−1.3 (−2.2, −0.4)	0.0744

Data are presented as least square mean (80% CI), based on a linear regression model controlling for baseline weight, baseline lean body mass, age and sex.

^a^Calculated as least square mean difference (apitegromab to placebo) divided by least square mean for placebo in lean mass loss.

Changes in other secondary endpoints or body composition parameters, including visceral fat, subcutaneous fat and trunk fat, were similar between apitegromab and placebo, as shown in Table 2. The study also included an exploratory assessment at week 32, 8 weeks after both interventions were discontinued, to evaluate durability of effect. At this timepoint, the difference in lean mass between apitegromab and placebo remained significant (least square mean (80% CI), 0.9 (0.3−1.5); nominal, *P* = 0.0480; Fig. 2b). For additional exploratory endpoints at weeks 24 and 32, no notable differences were observed between apitegromab and placebo in change from baseline in cardiometabolic parameters or physical function, respectively (Extended Data Table 2). Post hoc exploratory efficacy analyses confirmed the primary analysis, with statistically significant treatment effect favoring apitegromab; the results showed that treatment effect estimates were not influenced by site and that the results were directionally consistent across sex (Extended Data Table 3).

### Safety

The incidence of AEs was generally similar between apitegromab (76% (39/51), 95% CI: 63−86%) and placebo (71% (36/51), 95% CI: 57−81%). Overall, gastrointestinal AEs were the most frequently reported (Table 3). AEs that occurred in 10% or more of apitegromab-treated participants with 5% or higher frequency than in placebo-treated participants included nausea, fatigue and headache. Most of these AEs were mild and resolved with continued treatment, and none was an SAE or led to treatment discontinuation. SAEs in each treatment group (apitegromab: 2% (1/51), 95% CI: 0−10%; placebo: 2% (1/51), 95% CI: 0−10%) and AEs leading to discontinuation (apitegromab: 4% (2/51), 95% CI: 1−13%; placebo: 6% (3/51), 95% CI, 2−16%) were balanced, with none determined to be related to apitegromab. Two participants who received apitegromab discontinued treatment. One participant had an SAE of upper abdominal pain that resolved and was considered related to tirzepatide. The second participant had a non-serious AE of increased creatine phosphokinase (CPK) after initiation of a vigorous strength training program and discontinued study drug due to protocol-specified criteria. The event was resolved and was attributed to physical activity.

**Table 3:** Summary of AEs^a^

Adverse events^b^, *n* (% [95% CI])	Apitegromab 10 mg kg^−1^ + tirzepatide *n* = 51	Placebo + tirzepatide *n* = 51	Risk difference, apitegromab − placebo, % (95% CI)
Any AE	39 (76 [63−86])	36 (71 [57−81])	6% (−11, 22)
Study drug related	22 (43 [31−57])	19 (37 [25−51])	6% (−13, 24)
Tirzepatide related	30 (59 [45−71])	30 (59 [45−71])	0% (−18, 18)
Any SAE	1 (2 [0, 10])	1 (2 [0, 10])	0% (−8, 8)
Study drug related	0 (0 [0, 7])	0 (0 [0, 7])	NA
Tirzepatide related	1 (2 [0, 10])	0 (0 [0, 7])	2% (−5, 10)
Any AE grade^c^ ≥3	2 (4 [1, 13])	3 (6 [2, 16])	−2% (−12, 8)
AE leading to study drug discontinuation	2 (4 [1, 13])	3 (6 [2, 16])	−2% (−12, 8)
Abdominal pain upper	1 (2 [0, 10])^d^	0 (0 [0, 7])	2% (−5, 10)
Blood CPK increased	1 (2 [0, 10])^e^	0 (0 [0, 7])	2% (−5, 10)
Transaminases increased	0 (0 [0, 7])	1 (2 [0, 10])^f^	−2% (−10, 5)
Obsessive thoughts	0 (0 [0, 7])	1 (2 [0, 10])	−2% (−10, 5)
Pruritus	0 (0 [0, 7])	1 (2 [0, 10])	−2% (−10, 5)
AEs reported in ≥10% of participants in either group			
Nausea	20 (39 [27, 53])	16 (31 [20, 45])	8% (−10, 25)
Fatigue	13 (25 [16, 39])	6 (12 [6, 23])	14% (−2, 28)
Headache	11 (22 [12, 35])	5 (10 [4, 21])	12% (−3, 26)
Dyspepsia	9 (18 [10, 30])	10 (20 [11, 32])	−2% (−17, 13)
Constipation	8 (16 [8, 28])	9 (18 [10, 30])	−2% (−17, 13)
Diarrhea	7 (14 [7, 26])	11 (22 [12, 35])	−8% (−23, 7)
Vomiting	6 (12 [6, 23])	6 (12 [6, 23])	0% (−13, 13)
Upper respiratory tract infection	6 (12 [6, 23])	5 (10 [4, 21])	2% (−11, 15)

Wilson CIs are presented for the treatment group percentages, and Newcombe−Wilson CIs are presented for the risk differences.

^a^Includes data available for all participants who received at least one dose of study treatment.

^b^Medical Dictionary for Regulatory Activities version 27.1 Preferred Terms.

^c^All AEs and SAEs were graded using the National Cancer Institute CTCAE grading scale, version 5.0 or higher.

^d^SAE not considered related to apitegromab.

^e^AE not considered related to apitegromab.

^f^SAE not considered related to study drug.

CTCAE, Common Terminology Criteria for Adverse Events; NA, not applicable.

Clinical laboratory results, vital signs and electrocardiogram parameters were assessed for all patients receiving treatment at multiple timepoints; no notable findings were observed between the apitegromab and placebo groups (Extended Data Table 4). No patients met Hyʼs law or experienced a QT interval corrected using Fridericiaʼs formula (QTcF) >480 ms at any timepoint. Among those receiving apitegromab, 6% (3/51) tested positive for postbaseline antidrug antibodies (ADAs); the ADAs were transient (that is, isolated to one or two visits and negative at the last visit) and associated with the lowest possible titer (≤10). No notable safety events were observed in the participants with these transiently positive ADAs, including no hypersensitivity reactions and no SAEs or AEs leading to discontinuation related to apitegromab.

## Discussion

GLP-1 receptor agonists and GLP-1/GIP dual receptor agonists are highly effective for weight reduction in patients with obesity; however, they are associated with loss of lean mass and corresponding potential for undesirable health risks. Consistent with previous studies^4^, lean mass loss (80% CI) represented 30.2% (26.4−33.9%) of total weight loss among adults receiving tirzepatide and placebo in EMBRAZE. By contrast, only 14.6% (10.5−18.7%) of total weight loss was attributed to lean mass with tirzepatide and apitegromab 10 mg kg^−1^. Selective myostatin inhibition by apitegromab resulted in a 54.9% retention of lean mass relative to placebo, with similar total weight loss between treatment arms, indicating preservation of lean mass in the context of GLP-1 weight management. These findings extend trial results demonstrating improved function in SMA, suggesting a broader therapeutic potential of selective myostatin inhibition.

The observed elevation in total serum latent myostatin supports robust and durable target engagement in study participants receiving apitegromab. Although serum apitegromab concentrations varied over two-fold among study participants, minimal differences were observed in total latent myostatin levels, indicating that the 10 mg kg^−1^ dose is sufficient to achieve target saturation.

Apitegromab treatment was well tolerated, with similar incidence of AEs, SAEs and AEs leading to discontinuation between apitegromab and placebo. The favorable safety profile is consistent with apitegromab’s mechanism of action and observed AEs consistent with the safety profile of tirzepatide^17^. Apitegromab selectively targets structurally distinct myostatin precursors with no binding of other targets, thus minimizing undesirable side effects^11,18,19^. This aligns with previous reports that myostatin mutations across species did not cause long-term AEs and with previous studies investigating apitegromab in individuals with SMA^9,14,16,19^.

Several limitations should be considered when interpreting these results. The primary efficacy analysis is based on participants who completed the 24 weeks of treatment, although supportive analysis based on the intention-to-treat (ITT) population showed consistent findings. Additionally, a longer study would enable a more definitive assessment of sustained effects on body composition and long-term safety. Individuals with clinically significant cardiometabolic abnormalities, including diabetes, were excluded, limiting assessment of the effect of lean mass preservation on cardiometabolic parameters. The study population was relatively small and predominantly female, thus potentially limiting our ability to assess effect size across sex. As such, a larger study with broader eligibility criteria would augment generalizability of the study outcomes. The average baseline weight was somewhat higher in the placebo group; however, baseline weight was prespecified in the statistical models analyzing body composition to account for potential confounding. Although participants received standardized counseling regarding physical activity, levels of physical activity were not objectively collected; thus, its variability has the potential to confound data interpretation. Methodologically, DEXA scan has a limitation in differentiating compartments within lean mass, with accuracy impacted by hydration levels. Additionally, a two-sided significance level of 20% was used for this phase 2 study (without adjustment for multiplicity), and, therefore, CI widths must be interpreted only within that context. Notwithstanding these limitations, findings from our study showing increased lean mass preservation after apitegromab treatment are in alignment with the well-established myostatin biology as well as evidence in preclinical models and consistent clinical findings in SMA studies^9,16^. Taken together, results from this phase 2 trial illustrate the broader therapeutic potential of selective myostatin inhibition to preserve lean mass in the setting of pharmacological weight management. Extensive literature has established the role of myostatin in muscle regulation from early development into adulthood. To our knowledge, EMBRAZE marks the first study with participants over 21 years of age to demonstrate the efficacy of apitegromab.

Incretin mimetic therapies are effective pharmacological treatments for individuals with overweight and obesity; however, consistent with other weight loss approaches, a substantial portion of total body weight loss is due to lean mass loss. Results from EMBRAZE demonstrate clinical proof of concept for a highly selective antimyostatin antibody to preserve lean mass with tirzepatide therapy.

## Methods

### Trial details

EMBRAZE was a randomized, double-blind, placebo-controlled, phase 2 trial (NCT06445075) conducted at seven sites in the United States to evaluate the efficacy, safety, pharmacokinetics and pharmacodynamics of apitegromab in adults receiving tirzepatide. The trial protocol was approved by Advarra, a central institutional review board (IRB), as well as IRBs at participating sites and adhered to the Declaration of Helsinki and Good Clinical Practice guidelines. IRBs at participating sites included ProSciento CRU (Chula Vista, California); AdventHealth Translational Research Institute (Orlando, Florida); Great Lakes Clinical Trials, LLC, dba Flourish Research (Chicago, Illinois); Tandem Clinical Research GI, LLC (Marrero, Louisiana); Alliance for Multispecialty Research, LLC (Norman, Oklahoma); Apex Mobile Clinical Research (Bellaire, Texas); and Clinical Trials of Texas, LLC, dba Flourish Research (San Antonio, Texas). The study protocol was amended from version 1 to version 2 prior to enrollment of the first participant to add tirzepatide as an incretin mimetic therapy and to designate AEs of special interest (pancreatitis, liver injury, depression/suicidality and creatine kinase elevations).

### Participants

Eligible participants were aged 18−65 years with either a BMI of ≥27.0 to <30 kg m^−^^2^ and at least one weight-related comorbid condition or a BMI of ≥30.0 to ≤45 kg m^−^^2^. Stable body weight (±5 kg) within 90 days of screening and at least one self-reported unsuccessful dietary effort to lose weight were also required. Females of childbearing potential were required to have a negative pregnancy test and agree to use adequate birth control throughout trial participation. In addition, participants were required to adhere to the study visit schedule and the prespecified prohibitions and restrictions (Study Protocol).

Individuals with a history of or active cardiovascular disease were ineligible, including clinically significant arrhythmias; congestive heart failure (New York Heart Association grades I−IV); mild, moderate or severe coronary artery disease or history of angina; uncontrolled hyperlipidemia; myocardial infarction, stroke, coronary artery bypass graft surgery or percutaneous coronary intervention; valve disorders or defects; abdominal aortic aneurysm; or pulmonary hypertension. Also excluded were individuals with uncontrolled hypertension (American Heart Association stage 1 or stage 2) or history of hypertensive crisis or treatment-resistant hypertension; history of or active ischemic, hemorrhagic or anatomical neurovascular disease, including transient ischemic attack, cerebrovascular accident, arteriovenous malformation or brain aneurysm, peripheral vascular disease such as deep vein thrombosis/pulmonary embolism, chronic venous insufficiency, claudication or lymphedema; active pulmonary disease, including chronic obstructive pulmonary disease, pulmonary fibrosis, moderate-to-severe sleep apnea and moderate-to-severe asthma; hepatic, pancreatic, neuromuscular and/or psychiatric disease; active malignancy (other than local subcutaneous squamous cell and basal cell carcinomas); or history of immunosuppressive, chemotherapeutic or radiation treatment within 12 months prior to screening. Individuals with a history of type 1 or type 2 diabetes were also excluded. Type 2 diabetes that was resolved for more than 12 months and prediabetes managed with lifestyle and diet were not exclusions. Additional exclusion criteria included history of gastroparesis, gastric or peptic ulcer, active gastritis or esophagitis, uncontrolled gastroesophageal reflux disease or severe inflammatory bowel disease; uncontrolled thyroid disease; severe endocrine disorders such as Cushingʼs disease, hypogonadism and growth hormone deficiency; autoimmune or inflammatory disorders that may cause muscle wasting, such as myasthenia gravis, rheumatoid arthritis, lupus, polymyositis or dermatomyositis; neuromuscular disorders that may cause muscle wasting, such as muscular dystrophy, spinal muscular atrophy or amyotrophic lateral sclerosis; neurologic diseases such as epilepsy, dementia, Parkinsonʼs disease or Bellʼs palsy; acute or chronic pancreatitis (or at high risk for pancreatitis), clinically significant abnormal lipase and/or amylase or taking medications that may cause serious damage to the pancreas, such as valproic acid; history of or active acute or chronic liver disease; uncontrolled psychiatric disease; severe coagulopathy; chronic kidney disease stages 1−5; any chronic active infection or treatment of infection within 6 months prior to screening; bariatric surgery or use of gastric balloons or other gastric volume reduction devices; or donation or loss of ≥500 ml (1 pint) of blood within 8 weeks prior to screening or plasma donation of ≥600 ml within 14 days prior to screening.

Individuals with a history of apitegromab treatment were ineligible. Other medication-related exclusions included use of antiobesity medications, nutritional supplements or over-the-counter products for weight loss within 3 months prior to screening; use of antidiabetic medications, nutritional supplements or over-the-counter products for lowering blood sugar within 3 months prior to screening; use of medications known to induce weight gain within 3 months prior to screening; use of therapies with potentially significant muscle effects (for example, androgens, insulin-like growth factor, growth hormone, systemic beta-agonists, neurotoxins or muscle relaxants or muscle-enhancing supplements) in any form within 3 months prior to screening; use of systemic corticosteroids within 60 days before screening or intraarticular corticosteroid injection (inhaled or topical steroids were allowed); or medications that impede coagulation or platelet aggregation. History of alcoholism or illicit drug use or the use of inhaled vasoconstrictive tobacco or cannabis or synthetic products, such as vape pens, pipes, cigars and cigarettes, were prohibited. Individuals were also ineligible if they had any contraindications to study treatment or if, in the opinion of the investigator, they had any other medical condition, clinically significant laboratory result or electrocardiogram findings that may compromise safety or compliance, preclude the participant from successful study completion or interfere with interpretation of results.

### Procedures

Baseline characteristics were collected via standard assessments and a complete physical examination (see Study Protocol for additional details). Demographic information, such as age, sex, race and ethnicity, were self-reported by eligible participants. Participant sex was not considered in the study design; however, it was used as a covariate in the data analysis as described in the statistical methods section, and post hoc subgroup analysis of the primary endpoint by sex was performed on an exploratory basis. All participants received standard care counseling with regard to lifestyle recommendations such as diet, physical activity and behavior modification; however, reporting on these variables was not mandated or monitored.

Participants were randomized (1:1 via an interactive web response system managed by an independent vendor, without blocking or stratification factors) to receive apitegromab plus incretin mimetic therapy or placebo plus incretin mimetic therapy. The study sponsor, study participants, investigators and site personnel, with the exception of the pharmacist, were blinded to treatment assignments. The site pharmacist remained unblinded throughout the duration of the trial. DEXA scans were performed and assessed by blinded personnel, and statistical analyses were planned prior to unblinding at the end of the treatment period.

Apitegromab and placebo treatments were administered intravenously. After a 4-week screening period and written informed consent, site personnel administered an initial dose of tirzepatide 2.5 mg plus apitegromab or placebo (day 1). Thereafter, participants were trained to administer weekly doses of tirzepatide using an injection pen for 24 weeks. Tirzepatide dose was increased by 2.5 mg every 4 weeks according to the recommended dose-escalation schedule^20^ and subject to treatment response and tolerability up to a maximum maintenance dose of 15 mg per week. No participants received semaglutide as a treatment assignment, due to a lack of drug availability at the time of EMBRAZE study initiation, which lasted throughout study duration. Participants received apitegromab or placebo every 4 weeks for 24 weeks, followed by a 16-week safety period during which study treatments were not administered.

Concomitant therapies or interventional procedures medically indicated as part of standard supportive care for the participant were permitted at the discretion of the investigator. Antiobesity medications, antidiabetic medications or medications associated with serious risk to the pancreas (for example, valproic acid and diuretics), potentially significant muscle anabolic or catabolic effects or weight gain were prohibited.

### Endpoints and assessments

The primary endpoint was change from baseline in lean body mass at 24 weeks as assessed by whole-body DEXA (Hologic and GE Lunar) scans in participants receiving apitegromab with tirzepatide compared to those receiving tirzepatide alone. Specifically, each DEXA scan included bilateral arms, bilateral legs and trunk (including the body organs). Cross-calibration between DEXA systems was not warranted. Secondary endpoints included change from baseline in body weight, total fat mass and DEXA parameters on body composition at week 24. Body weight was obtained using a calibrated scale in a fasted state at each visit from screening through the last trial visit. For primary and secondary endpoints based on DEXA measurements, scans were obtained within 7 days prior to participants’ initial dose of apitegromab or placebo and were repeated at 24 weeks. Exploratory endpoints included change from baseline in DEXA measurements at week 32, physical function (force production with handheld dynamometry and number of repetitions in the chair sit-to-stand test) at weeks 24 and 32 and cardiometabolic markers (for example, hemoglobin A1C (HbA1C)) at weeks 24 and 32.

Prespecified pharmacokinetics and pharmacodynamics endpoints were assessed through the treatment and safety follow-up periods for all participants who received at least one dose of apitegromab or placebo. Apitegromab and tirzepatide trough samples were collected every 4 weeks during the treatment period and during safety follow-up visits, and end-of-infusion samples of apitegromab were collected on day 1, week 12 and week 20. Trough concentrations of latent myostatin were collected through 24 weeks and during the safety follow-up period.

Safety endpoints included the frequency of AEs, clinical laboratory tests, vital signs, electrocardiogram measurements and psychiatric evaluations reported through the last study visit. Data were reviewed throughout the study in a blinded manner by medical monitors and the sponsor to ensure participant safety. The presence of serum ADAs against apitegromab was also assessed. Safety data were collected through week 40 of the study.

### Statistical analysis

The primary population for efficacy analyses consisted of all randomized participants who completed treatment and had evaluable DEXA lean body mass data at baseline and at the scheduled week 24 visit (designated as the completer population in the Statistical Analysis Plan). Most efficacy analyses were repeated on the ITT population (all randomized participants) and the modified ITT population (all randomized participants with evaluable baseline and one postbaseline lean body mass measurement, regardless of timing). Safety analyses included all dosed participants, and pharmacokinetics/pharmacodynamics analyses included all participants with quantifiable pharmacokinetics/pharmacodynamics data. Analyses were conducted using SAS version 9.4 software.

All efficacy, safety and pharmacokinetics/pharmacodynamics endpoints are summarized descriptively by treatment group. Least square means together with 80% CIs for each treatment group and the difference between groups in change from baseline at week 24 in lean body mass were estimated using a linear regression model controlling for baseline weight, baseline lean body mass, age and sex. The same regression model was used to estimate least square means and differences between groups with their associated CIs for the changes in other DEXA parameters and the change in weight. Post hoc analyses for the primary endpoint included a subgroup analysis by sex and a sensitivity analysis exploring effects of study sites.

A two-sided significance level of 0.20 was selected for this phase 2 study investigating the effects of apitegromab in this population. All *P* values are nominal with no adjustment for multiplicity, and the CIs presented should be interpreted within this context.

Assuming a standard deviation of 4.6 kg, an evaluable sample size of 43 participants per study arm was determined to yield approximately 75% power to detect an effect size of 2 kg for the primary endpoint of lean body mass change from baseline at week 24. Anticipating that approximately 13% would not be evaluable for the primary analysis, a total of 50 participants per arm was planned.

### Reporting summary

Further information on research design is available in the Nature Portfolio Reporting Summary linked to this article.

## Online content

Any methods, additional references, Nature Portfolio reporting summaries, source data, extended data, supplementary information, acknowledgements, peer review information; details of author contributions and competing interests; and statements of data and code availability are available at 10.1038/s41591-026-04440-4.

## Supplementary information

Supplementary Information Study protocol and statistical analysis plan.

Reporting Summary

## Figures and Tables

**Extended Data Table 1:**
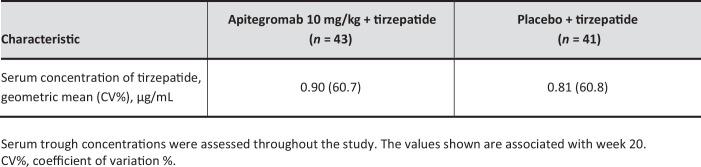
Serum trough concentration of tirzepatide

**Extended Data Table 2:**
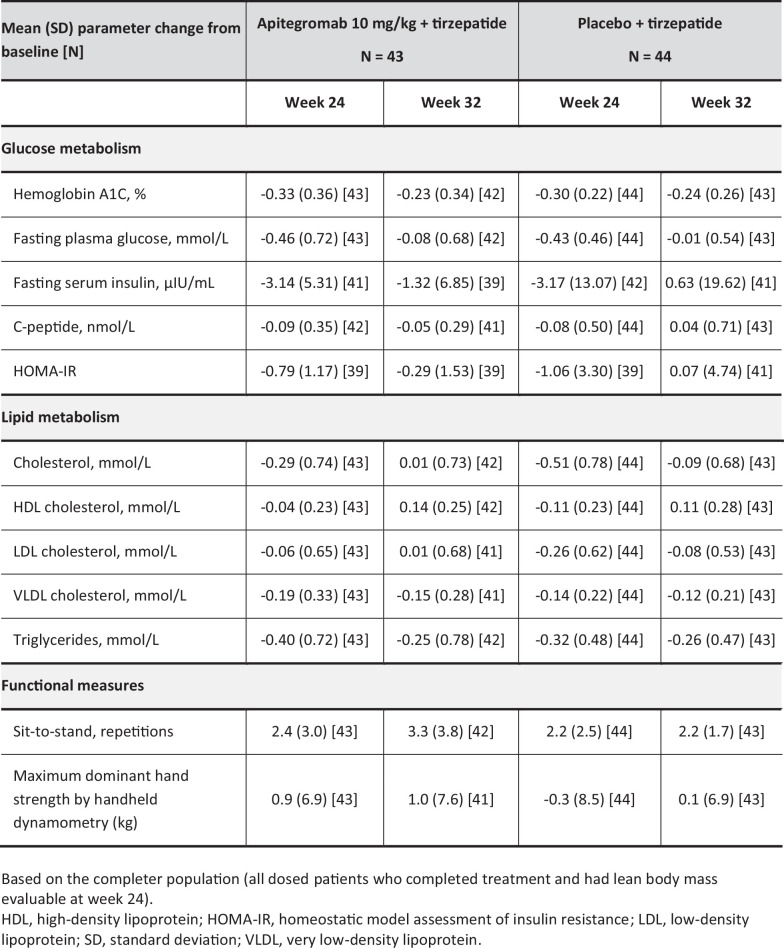
Exploratory cardiometabolic parameters and physical function assessments at weeks 24 and 32

**Extended Data Table 3:**
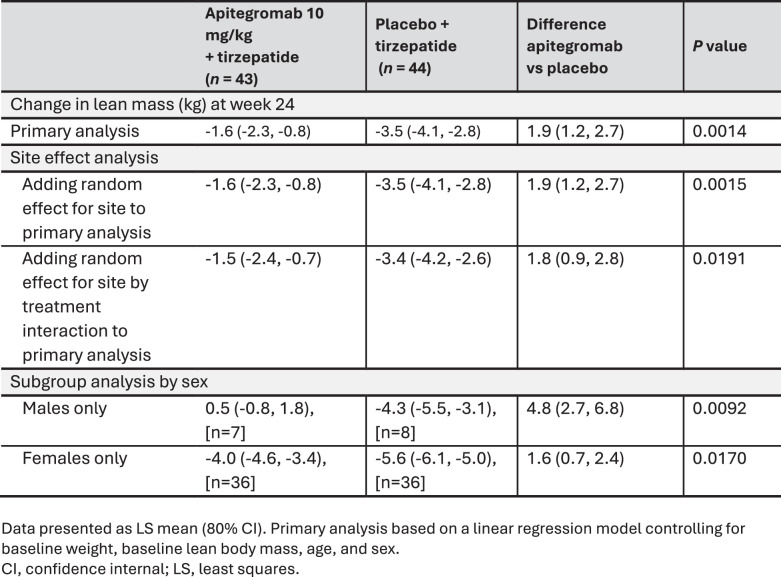
Change in lean mass (kg) at week 24, post hoc exploratory analyses

**Extended Data Table 4:**
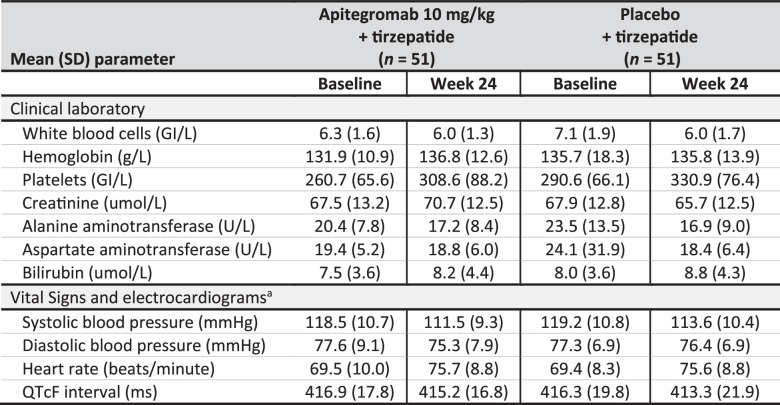
Additional safety parameters

^a^Mean of electrocardiogram triplicate values used for summaries.

## Data Availability

Scholar Rock, Inc. is committed to sharing deidentified clinical trial data with external investigators upon reasonable request. Individual researchers requesting clinical trial data for academic or non-commercial use must reach out to Scholar Rock (medicalinformation@scholarrock.com) and include a research proposal clarifying how the data will be used, including proposed analysis methodology. Inquiring researchers should anticipate a response acknowledging their request within 2−3 business days. Scholar Rock will consider and evaluate unsolicited requests for clinical trial data on a case-by-case basis.
