# Supplementary material for: Apitegromab for lean mass preservation during tirzepatide-induced weight loss: a randomized, double-blind, placebo-controlled phase 2 trial
Source: Nat Med. 2026 Jun 8;32(7):2673–8. doi: 10.1038/s41591-026-04440-4 (PMC13375528; doi:10.1038/s41591-026-04440-4)
Supplement: Supplementary file 1 — Study protocol and statistical analysis plan. [file 41591_2026_4440_MOESM1_ESM.pdf]

nature medicine

Supplementary information https://doi.org/10.1038/s41591-026-04440-4

Apitegromab for lean mass preservation during tirzepatide-induced weight loss: a randomized, double-blind, placebo-controlled phase 2 trial

In the format provided by the authors and unedited

APITEGROMAB (SRK-015)

SRK-015-006 CLINICAL STUDY PROTOCOL

A PHASE 2A RANDOMIZED, DOUBLE-BLIND, PLACEBO-CONTROLLED, MULTICENTER STUDY TO EVALUATE THE EFFICACY, SAFETY, AND PHARMACOKINETICS OF APITEGROMAB IN OVERWEIGHT AND OBESE ADULT SUBJECTS (EMBRAZE)

**Protocol Number:**	SRK-015-006
**Brief Title:**	Efficacy and Safety of Apitegromab for the Treatment of Adults who are Overweight or Obese
**Indication Studied:**	Overweight and Obesity
**Study Phase:**	2a
**Investigational Product:**	Apitegromab (SRK-015)
**IND Number:**	169049
**Sponsor:**	Scholar Rock, Inc.<br>301 Binney Street, 3rd Floor<br>Cambridge, MA, 02142<br>USA
**Sponsor Contact:**	[redacted]
**Protocol Date and Version:**	07 December 2023, Original Protocol Version 1.0<br>04 March 2024, Version 2.0

CONFIDENTIAL

This document contains confidential information. Any use, distribution, or disclosure without the prior written consent of Scholar Rock is strictly prohibited except to the extent required under applicable laws or regulations. Persons to whom the information is disclosed must be informed that the information is confidential and may not be further disclosed by them.

SPONSOR SIGNATURE

I have read and approve this protocol. My signature, in conjunction with the signature of the Principal Investigator, confirms the agreement of both parties that the clinical study will be conducted in accordance with the protocol and all applicable laws and regulations, including, but not limited to, the International Council for Harmonisation (ICH) Guidelines for Good Clinical Practice (GCP), the United States Code of Federal Regulations (US CFR), and the ethical principles that have their origins in the Declaration of Helsinki. In addition, I will inform the Principal Investigator and all other Investigators of all relevant information that becomes available during the conduct of this study.

Sponsor's Authorized Officer:

[redacted]

INVESTIGATOR'S AGREEMENT

I have received and read this clinical protocol for Study SRK-015-006 dated [redacted]. My signature, in conjunction with the signature of the Sponsor, confirms the agreement of both parties that the clinical study will be conducted in accordance with the protocol and all applicable laws and regulations, including, but not limited to, the ICH Guideline for GCP, the US CFR, and the ethical principles that have their origins in the Declaration of Helsinki.

Nothing in this document is intended to limit the authority of a physician to provide emergency medical care under applicable regulations.

[redacted]

TABLE OF CONTENTS

SPONSOR SIGNATURE 2
INVESTIGATOR'S AGREEMENT 3
TABLE OF CONTENTS 4
LIST OF TABLES 9
LIST OF FIGURES 10
LIST OF ABBREVIATIONS AND DEFINITION OF TERMS 11
1. PROTOCOL SUMMARY 15
1.1. Synopsis 15
1.2. Schedule of Assessments 23
2. INTRODUCTION 27
2.1. Study Rationale 27
2.2. Background 28
2.2.1. Obesity 28
2.2.2. Adiposity Assessment 29
2.2.3. Weight Loss and Lean Body Mass Loss 29
2.2.4. Overweight and Obesity 30
2.2.5. Current Therapies 30
2.2.6. Apitegromab 30
2.2.6.1. Mechanism of Action 31
2.2.6.2. Nonclinical Pharmacology 32
2.2.6.3. Summary of Safety of Apitegromab in SMA 32
2.2.6.4. Summary of Efficacy of Apitegromab in SMA 32
2.3. Benefit/Risk Assessment for Apitegromab 33
2.3.1. Benefit Assessment 33
2.3.2. Risk Assessment 33
2.3.3. Overall Benefit/Risk Conclusion 34
3. STUDY OBJECTIVES AND PURPOSE 36
4. INVESTIGATIONAL PLAN 38
4.1. Overall Study Design 38
4.2. Scientific Rationale for Study Design 39
4.3. Number of Subjects 39

4.4. Treatment Assignment 39
4.5. Dosing 40
4.5.1. Dose Justification 40
4.5.1.1. Apitegromab 40
4.5.1.2. Incretin Mimetic Therapy 41
4.5.2. Dose Adjustment Criteria 42
4.5.2.1. Apitegromab or Placebo 42
4.5.2.2. Incretin Mimetic Therapy 42
4.5.3. Missed Doses 42
4.5.3.1. Apitegromab or Placebo 42
4.5.3.2. Incretin Mimetic Therapy 43
5. SELECTION AND WITHDRAWAL OF SUBJECTS 44
5.1. Subject Inclusion Criteria 44
5.2. Subject Exclusion Criteria 44
5.3. Lifestyle Considerations 47
5.4. Screen Failures 47
5.5. Criteria for Temporarily Delaying Randomization/Administration of Study Drug 48
5.6. Subject Withdrawal Criteria 48
5.6.1. Discontinuation of Study Drug and/or Study 48
5.6.1.1. Suspension of Dosing for all Subjects 48
5.6.1.2. Discontinuation of Study Treatment and/or Study Participation 48
5.6.2. Lost to Follow-up 50
6. TREATMENT OF SUBJECTS 52
6.1. Description of Study Drug 52
6.1.1. Apitegromab or Placebo 52
6.1.2. Incretin Mimetic Therapy 53
6.1.2.1. Tirzepatide 53
6.1.2.2. Semaglutide 53
6.1.2.3. Dose Modification 54
6.2. Concomitant Medications 55
6.3. Treatment Compliance 55
6.4. Randomization and Blinding 55

6.5. Dose Modification 56
6.6. Treatment of Overdose 56
6.6.1. Apitegromab or Placebo 56
6.6.2. Incretin Mimetic Therapy 57
6.7. Unscheduled Visit....57
6.8. Continued Access to Study Drug After the End of Treatment 57
6.9. Criteria for Study Termination 57
6.9.1. End of Study Definition....57
6.9.2. Study Termination 58
6.9.3. Site Closure....58
7. STUDY DRUG MATERIALS AND MANAGEMENT 59
7.1. Study Drug....59
7.2. Study Drug Packaging and Labeling 59
7.3. Study Drug Preparation/Handling/Storage/Accountability 59
7.3.1. Apitegromab or Placebo 59
7.3.2. Incretin Mimetic Therapy 60
7.4. Study Drug Administration....60
7.4.1. Apitegromab or Placebo 60
7.4.2. Incretin Mimetic Therapy 60
7.4.2.1. Tirzepatide 60
7.4.2.2. Semaglutide 61
8. ASSESSMENT OF EFFICACY 62
8.1. Anthropometric Measurements 62
8.1.1. Height 62
8.1.2. Weight....62
8.1.3. Waist and Hip Circumference Measurements 62
8.1.4. Body Mass Index Calculation....62
8.2. Handheld Dynamometry Assessment....62
8.3. Chair Sit-to-Stand Test 62
8.4. Efficacy Laboratory Tests....63
8.5. Blood Pressure....63
9. ASSESSMENT OF SAFETY....64
9.1. Safety Parameters 64

9.1.1. Demographics/Medical History/Weight Management History 64
9.1.2. Vital Signs 64
9.1.3. Physical Examination 64
9.1.4. Electrocardiogram....64
9.1.5. Safety Laboratory Tests 64
9.1.5.1. Biomarkers....65
9.1.6. Site Check-In Telephone Call....66
9.1.7. Pregnancy 66
9.1.8. Psychiatric Evaluations....67
9.1.8.1. Columbia Suicide Severity Rating Scale....67
9.1.8.2. Patient Health Questionnaire - 9 67
9.2. Adverse Events and Serious Adverse Events 67
9.2.1. Definition of Adverse Events 67
9.2.1.1. Special Cases 68
9.2.1.2. Events NOT Meeting the Definition of an Adverse Event....69
9.2.2. Procedure-related Adverse Events....69
9.2.3. Definition of Serious Adverse Events 69
9.2.4. Treatment-Emergent Adverse Event 70
9.2.5. Adverse Events of Special Interest 70
9.3. Common Terminology Criteria for Adverse Events/Serious Adverse Events 74
9.4. Adverse Event Monitoring 74
9.5. Recording Adverse Events and Serious Adverse Events 74
9.5.1. Assessment of Intensity 75
9.5.2. Assessment of Causality 75
9.5.3. Assessment of Expectedness 76
9.5.4. Instructions for Recording AEs in eCRF 76
9.5.4.1. Recording Diagnosis Versus Signs and Symptoms....76
9.5.4.2. Abnormal Laboratory Values or Vital Signs 77
9.6. Reporting Serious Adverse Events or AESIs 77
9.6.1. Reporting to Safety Using an Electronic Data Collection Tool 77
9.6.2. Reporting to Safety Using a Paper SAE/AESI Form 77
9.7. Time Period and Frequency for Collecting AE, SAE, and AESI Information....78
9.8. Follow-up of AEs, SAEs, and AESIs 78

9.8.1. Regulatory Reporting Requirements for SAEs/AESIs 78
9.9. Contraceptive and Barrier Guidance 79
9.9.1. Definitions 79
9.9.2. Contraception Guidance 79
9.9.3. Acceptable Methods of Contraception 80
10. STATISTICS 81
10.1. Statistical Hypotheses 81
10.2. Sample Size Determination 81
10.3. Analysis and Populations 81
10.4. Statistical Analyses 81
10.4.1. General Considerations 81
10.4.2. Primary Efficacy Endpoint Analysis 82
10.4.3. Secondary and Exploratory Efficacy Endpoint Analysis 82
10.4.4. Multiplicity 82
10.4.5. Safety Endpoints Analyses 82
10.4.6. Pharmacokinetic and Pharmacodynamic Endpoints Analyses 83
10.4.7. Anti-apitegromab Antibody Endpoint Analyses 83
10.4.8. Additional Endpoints Analyses 83
10.4.9. Subgroup Analyses 83
10.5. Interim Analysis 83
11. DIRECT ACCESS TO SOURCE DATA/DOCUMENTS 84
11.1. Study Monitoring 84
11.2. Audits and Inspections 84
11.3. Institutional Review Board/Independent Ethics Committee 85
12. QUALITY CONTROL AND QUALITY ASSURANCE 85
12.1. Protocol Adherence 85
13. ETHICS 85
13.1. Ethics Review 85
13.2. Ethical Conduct of the Study 85
13.3. Written Informed Consent 85
14. DATA HANDLING AND RECORDKEEPING 86
14.1. Inspection of Records 86
14.2. Retention of Records 86

15. PUBLICATION POLICY 86
16. LIST OF REFERENCES....87
17. APPENDICES 92
17.1. Appendix 1: Regulatory, Ethical, and Study Oversight Considerations 92
17.1.1. Regulatory and Ethical Considerations 92
17.1.2. Financial Disclosure 92
17.1.3. Informed Consent Process 93
17.1.4. Data Protection 93
17.1.5. Dissemination of Clinical Study Data 93
17.1.6. Data Quality Assurance 93
17.1.7. Source Documents 94

LIST OF TABLES

Table 1: Schedule of Assessments....23
Table 2: Body Mass Index and Obesity Classifications 30
Table 3: Study Objectives and Endpoints....36
Table 4: Simulated Exposure in Obese Subject (200 kg) and Comparison to Exposures in Prior Studies....41
Table 5: Specific Laboratory Abnormalities[a]/Clinical Findings Leading to Subject Discontinuation From Study Treatment/Study Participation 49
Table 6: Investigational Product 52
Table 7: Recommended Dose Escalation Schedule for Tirzepatide....53
Table 8: Recommended Dose Escalation Schedule for Semaglutide 54
Table 9: Efficacy Laboratory Assessments....63
Table 10: Safety Laboratory Assessments....65
Table 11: Urinalysis Assessments 65
Table 12: Clinical Evaluation of Drug-Induced Liver Injury 71
Table 13: Safety Monitoring Guidance for Creatine Kinase Elevations 73
Table 14: Adverse Event and Serious Adverse Event Assessment of Intensity....75
Table 15: Methods of Contraception Allowed During the Study....80

LIST OF FIGURES

Figure 1: Apitegromab Mechanism of Action 31

Figure 2: Overall Study Design..38

LIST OF ABBREVIATIONS AND DEFINITION OF TERMS

**Abbreviation or Specialist Term**	**Explanation**
A1c	glycated hemoglobin
ActRII	activin receptor II
ADA	antidrug antibody
ADL	activities of daily living
AE	adverse event
AESI	adverse event of special interest
ALP	adverse event of special interest
ALT	alkaline phosphatase
AST	alanine aminotransferase
AT	aspartate aminotransferase
BIW	aminotransferase
BMI	body mass index
BMP	bone morphogenetic protein
BMR	basal metabolic rate
C-SSRS	Columbia Suicide Severity Rating Scale
CDC	Center for Disease Control
CFR	Code of Federal Regulations
CK	creatine kinase
CL	clearance
CNS	central nervous system
COVID-19	Coronavirus disease 2019
CT	computed tomography
CRF	case report form
CTCAE	Common Terminology Criteria for Adverse Events
DEXA	dual-energy x-ray absorptiometry
ECG	electrocardiogram
eCRF	electronic case report form
EDC	electronic data collection
EOI	end of infusion
EOS	End of Study
EOT	End of treatment

Abbreviation or Specialist Term	Explanation
ET	Early Termination
Fc	fragment crystallizable
FDA	Food and Drug Administration
GCP	Good Clinical Practice
GDF11	growth differentiation factor 11
GLP-1 RA	glucagon-like peptide-1 receptor agonists
Hb	hemoglobin
HC	hip circumference
HFMSE	Hammersmith Functional Motor Scale – Expanded
HIPPA	Health Insurance Portability and Accountability Act
HIV	human immunodeficiency virus
ICF	informed consent form
ICH	International Council for Harmonisation
IC_{50}	concentration of apitegromab to achieve half the inhibiting effect
IEC	Independent Ethics Committee
IgG4	immunoglobulin G4
I_{max}	maximum inhibiting effect
IND	Investigational New Drug
INR	international normalized ratio
IRB	Institutional Review Board
ITT	Intention-to-Treat
IV	intravenous
IWRS	interactive web response system
K_{out}	first order degradation rate of total latent myostatin
LBM	lean body mass
LST	lean soft tissue
mAb	monoclonal antibody
MAD	multiple ascending dose
MedDRA	Medical Dictionary for Regulatory Affairs
MHP	mental health professional
MRI	magnetic resonance imaging
MTD	maximum tolerated dose

Abbreviation or Specialist Term	Explanation
NIH	National Institutes of Health
ONYX	Study SRK-015-004
PD	pharmacodynamic(s)
PHQ-9	Patient Health Questionnaire - 9
PK	pharmacokinetic(s)
PROMIS	Patient Reported Outcome Measurement Information System
PT	preferred term
QTL	quality tolerance limit
Q4W	once every 4 weeks
QW	weekly
RA	receptor agonist
RHS	Revised Hammersmith Scale
RULM	Revised Upper Limb Module
Rx	treatment
SAD	single ascending dose
SAE	serious adverse event
SAP	statistical analysis plan
SAPPHIRE	Study SRK-015-003
SAR	serious adverse reactions
SAT	subcutaneous adipose tissue
SaO_2	saturated oxygen in arterial blood
SCR	Screening (period)
SMA	spinal muscular atrophy
SMN	survival motor neuron protein
SoA	Schedule of Assessments
SOC	system organ class
SOI	start of infusion
SRK-015	Scholar Rock designation for apitegromab
T2D	Type 2 diabetes
TBWL	total body weight loss
TEAE	treatment-emergent adverse event
TEE	total energy expenditure

Abbreviation or Specialist Term	Explanation
TGF-β	transforming growth factor beta
TIW	Thrice weekly
TOPAZ	Study SRK-015-002
ULN	upper limit of normal
Unsch	unscheduled
US(A)	United States (of America)
V	Visit
VAT	visceral adipose tissue
V_c	volume of distribution – central
V_d	volume of distribution
V_p	volume of distribution – peripheral
WC	waist circumference
WHO	World Health Organization
WHR	waist-to-hip ratio
WHtR	waist-to-height-ratio

1. PROTOCOL SUMMARY

1.1. Synopsis

**Name of Sponsor/Company:** Scholar Rock, Inc., 301 Binney Street, 3rd Floor, Cambridge, MA 02142, USA		
**Name of Investigational Product:** Apitegromab (SRK-015)	**IND Number:** 169049	**Active Ingredient:** Apitegromab
**Protocol Number :** SRK-015-006	**Phase of Development:** 2a	**Country:** United States (US)

Title of Study:

A Phase 2a Randomized, Double-Blind, Placebo-Controlled, Multicenter Study to Evaluate the Efficacy, Safety, and Pharmacokinetics of Apitegromab in Overweight and Obese Adult Subjects

Study center(s): Approximately 20 study sites in the US

Study Rationale

This is a Phase 2a randomized, double-blind, placebo-controlled, multicenter study targeting enrollment of approximately 100 overweight or obese adults without diabetes (WHO 2023). The study will assess the efficacy, safety, and pharmacokinetics (PK) of apitegromab when administered as an adjunctive therapy to incretin mimetic therapy.

Adjunctive pharmacotherapy for weight loss is indicated for individuals with a body mass index (BMI) $\geq$30 kg/m^2 or $\geq$27 kg/m^2 in persons with 1 or more weight-related comorbid condition(s), who have not met weight loss goals with a lifestyle intervention approach (Acosta 2017, Jensen 2014). A growing number of incretin mimetic therapies are being developed for weight loss in patients with overweight or obesity and without Type-2 diabetes (T2D). Incretin mimetics, including glucagon-like peptide 1 (GLP-1) receptor agonists (RAs) and GLP-1 receptor and glucose dependent insulinotropic polypeptide (GIP) dual agonists, have been shown to lower body weight through decreased caloric intake, most likely through appetite suppression. Clinical use of semaglutide, a GLP-1 RA for obesity treatment, has led to robust improvements in weight loss (Bergmann 2023) and improvements in cardiometabolic risk factors and quality of life; however, common side effects include delayed gastric emptying, nausea, vomiting, and diarrhea (Joshi 2023, Klein 2023, Kobori 2023, Silveira 2023). Real-world evidence demonstrates sub-optimal adherence and/or discontinuation of GLP-1 RA therapy (Ghusn 2022, Weiss 2020). Tirzepatide, a GLP-1/GIP dual receptor agonist approved for obesity treatment, has also been shown to provide substantial and sustained reductions in body weight in patients with overweight or obesity with similar common side effects (Jastreboff 2022).

Rapid reversal of weight loss and cardiometabolic benefits has been observed following withdrawal of semaglutide (Rubino 2021, Wilding 2022) and tirzepatide (Aronne 2024) as well as other obesity therapeutics such as orlistat (Sjöström 1998, Smith 2010) despite continued lifestyle intervention. These observations highlight the chronic nature of obesity and the significant unmet need for improved tolerability and durability of pharmacological interventions.

Both dynamic weight loss and attempts to maintain a reduced weight are associated with a disproportionate decline in energy expenditure beyond that predicted solely on the basis of changes in body weight and composition; this decline in energy expenditure may contribute to weight regain. Importantly, a substantial portion (20% to 50%; Sargeant 2019) of total body weight loss (TBWL) mediated by incretin mimetic therapy may be attributed to loss of lean body mass (LBM), which is consistent with diet induced weight loss and bariatric surgery (Ida 2021, Sargeant 2019, Wilding

2021). Within this document, LBM is synonymous with lean soft tissue (LST), which is the sum of body water, total body protein, carbohydrates, nonfat lipids and soft tissue mineral (skeletal muscle, organs and connective tissue). Skeletal muscle makes up the largest portion of the LBM, which does not include bone (Prado 2014, Yu 2013). Fat free mass includes bone and LST (Prado 2014, Yu 2013). Increases in LBM have been shown to improve basal metabolic rate [BMR] (Aristizabal 2015), improve insulin sensitivity (Lindegaard 2008, Srikanthan 2011), reduce visceral adipose tissue (VAT) (Wewege 2022), and increase caloric expenditure postexercise (Zurlo 1990). Therefore, preservation of LBM during and after weight loss may be beneficial. Myostatin, a member of the transforming growth factor beta (TGF-β) superfamily of growth factors, is expressed primarily by skeletal muscle cells; the myostatin pathway is highly conserved from animals to humans. Myostatin is a secreted protein that negatively regulates skeletal muscle mass and function (Day 2022, Long 2019). Bimagrumab, an investigational monoclonal antibody (mAb) that inhibits myostatin via activin receptor II (ActRII) blockade, was evaluated in a Phase 2 study in adult overweight and obese subjects with T2D. Treatment with bimagrumab led to a gain in LBM, reduction in fat mass, and improvements in glycemic parameters (Heymsfield 2021). Therefore, combining inhibition of myostatin activation with incretin mimetic-driven weight loss may address the loss of LBM in this setting and consequently avoid the decline in energy expenditure associated with loss of lean muscle mass.

Apitegromab is an investigational, fully human immunoglobulin G4 (IgG4) monoclonal antibody (mAb) that specifically binds with high affinity to promyostatin and latent myostatin, the inactive precursor forms of myostatin, a growth factor of the TGF-β superfamily that is expressed predominantly in skeletal muscle and functions to restrict skeletal muscle size. Myostatin is initially produced in an inactive form, and 2 sequential proteolytic steps are required to release the active growth factor form of myostatin, which signals locally within skeletal muscle. Approaches which inhibit myostatin signaling are therefore expected to result in increased skeletal muscle mass and potential increases in strength. Apitegromab binds to both promyostatin and latent myostatin and prevents the release of the mature myostatin growth factor in skeletal muscle.

Apitegromab's high selectivity for latent myostatin, in addition to the lack of effect on signaling through other endogenous-TGF-β family members, is distinct from bimagrumab. Apitegromab is administered via intravenous (IV) infusion. Nonclinical studies demonstrated that selective inhibition of myostatin with apitegromab or its analog was associated with an increase in LBM in the mouse, rat, and cynomolgus monkey (Apitegromab CM Obesity IB, Long 2019). Clinical administration of apitegromab in pediatric and adult subjects with spinal muscular atrophy (SMA) was associated with improved motor function (as measured by Hammersmith Functional Motor Scale – Expanded [HFMSE]) and reduced fatigue (as measured by the Patient Reported Outcome Measurement Information System [PROMIS] Fatigue Scale) (Day 2022). Overall, the tolerability profile of apitegromab is favorable, with no identified safety risks.

This study will be evaluating the effects of apitegromab when used as an adjunctive therapy to incretin mimetic therapy in subjects with overweight or obesity and without diabetes. Taken together, the Sponsor believes that apitegromab in the setting of incretin mimetic-driven weight loss has the potential to attenuate the loss of LBM associated with incretin mimetic therapy alone, thus improving body composition and mitigating the reductions in metabolic rate, which in turn could further enhance fat loss.

Objectives and Endpoints:

**Objectives**	**Endpoints**
**Primary**	
• Evaluate the efficacy of apitegromab versus placebo when used as an adjunctive therapy to incretin mimetic therapy in subjects with overweight or obesity and without diabetes	• Change from Baseline at 24 weeks in LBM
**Secondary**	
• Evaluate the effect of apitegromab versus placebo on body weight when used as an adjunctive therapy to incretin mimetic therapy in subjects with overweight or obesity and without diabetes	• Change from Baseline at 24 weeks in body weight
• Evaluate the effect of apitegromab versus placebo on body composition when used as an adjunctive therapy to incretin mimetic therapy in subjects with overweight or obesity and without diabetes	• Change from Baseline at 24 weeks in DEXA measurements, including:<br>○ Percent LBM<br>○ Total and percent of fat body mass<br>○ Total and percent of VAT, SAT, and trunk fat body mass<br>○ Percent of weight loss from Baseline due to fat body mass loss or LBM
• Evaluate the PK and PD of apitegromab when used as an adjunctive therapy to incretin mimetic therapy in subjects with overweight or obesity and without diabetes	• Trough and EOI concentration of apitegromab through 24 weeks of treatment<br>• Trough concentrations of latent myostatin through 24 weeks of treatment<br>• Concentration of apitegromab and latent myostatin during Safety Follow-up Period (Week 25 through Week 40)
• Evaluate the safety and tolerability of apitegromab when used as an adjunctive therapy to incretin mimetic therapy in subjects with overweight or obesity and without diabetes	• Frequency of TEAEs and SAEs by severity<br>• Change from Baseline in clinical safety laboratory tests, vital signs, ECG measurements, and psychiatric evaluations<br>• Presence or absence of ADAs against apitegromab in serum from blood samples
**Exploratory**	

• Evaluate exploratory efficacy parameters of apitegromab versus placebo when used as an adjunctive therapy to incretin mimetic therapy in subjects with overweight or obesity and without diabetes	• Change from Baseline at 32 weeks in DEXA measurements:<br>○ Proportion of subjects with categorical percent changes from Baseline in body weight, fat body mass, and/or LBM<br>• Proportion of subjects with categorical changes from Baseline in waist circumference<br>• Proportion of subjects with change from Baseline in WHtR and WHR categories<br>• Change from Baseline in force production assessed by handheld dynamometry<br>• Change from Baseline in the number of sit-to-stand repetitions in the chair sit-to-stand test
• Evaluate effect of apitegromab versus placebo on glucose metabolism, lipid metabolism, and systolic and diastolic blood pressure when used as an adjunctive therapy to incretin mimetic therapy in subjects with overweight or obesity and without diabetes	• Change from Baseline in glucose metabolism assessments, including plasma glucose, HbA1c, serum insulin, C-peptide, and HOMA-IR values<br>• Change from Baseline in lipid panel tests including total cholesterol, triglycerides, LDL, HDL, and VLDL<br>• Change from Baseline in systolic and diastolic blood pressure
• Evaluate effect of apitegromab versus placebo on an exploratory biomarker when apitegromab is used as an adjunctive therapy to incretin mimetic therapy in subjects with overweight or obesity and without diabetes	• Change from Baseline in adiponectin values
• Evaluate the PK of incretin mimetic therapy in the presence and absence of apitegromab in subjects with overweight or obesity and without diabetes	• Concentration of incretin mimetic therapy through 24 weeks of treatment

Abbreviations: A1c, glycated hemoglobin; ADA, antidrug antibody; DEXA, dual-energy x-ray absorptiometry; ECG, electrocardiogram; EOI, endo of infusion; Hb, hemoglobin; LBM, lean body mass; PD, pharmacodynamics; PK, pharmacokinetics; SAE, serious adverse event; SAT, subcutaneous adipose tissue; TEAE, treatment-emergent adverse event; VAT, visceral adipose tissue; VLDL, very low density lipoprotein; WHR, waist-to-hip ratio; WHtR, waist-to-height ratio.

Study Design:

This Phase 2a randomized, double-blind, placebo-controlled, multicenter study will be conducted to evaluate the efficacy, safety, and PK of apitegromab in adult subjects with overweight or obesity and without diabetes at approximately 20 study sites in the US. Approximately 100 overweight or obese adult subjects without diabetes will be randomized 1:1 to receive apitegromab 10 mg/kg Q4W + incretin mimetic therapy weekly (QW) or placebo Q4W + incretin mimetic therapy QW. Administration of incretin mimetic therapy will be based on a dose escalation schedule, beginning at the lowest dose and escalating Q4W to the next dose until a recommended maximum tolerated maintenance dose has been reached.

As shown below, the study will include a Screening Period (up to 4 weeks), a Treatment Period, and a Safety Follow-up Period. Subjects completing the 24-week (169-day) Treatment Period will be

followed for 16 weeks (112 days) in the Safety Follow-up Period, during which time they will not be receiving any study drug (apitegromab, placebo, or incretin mimetic therapy).

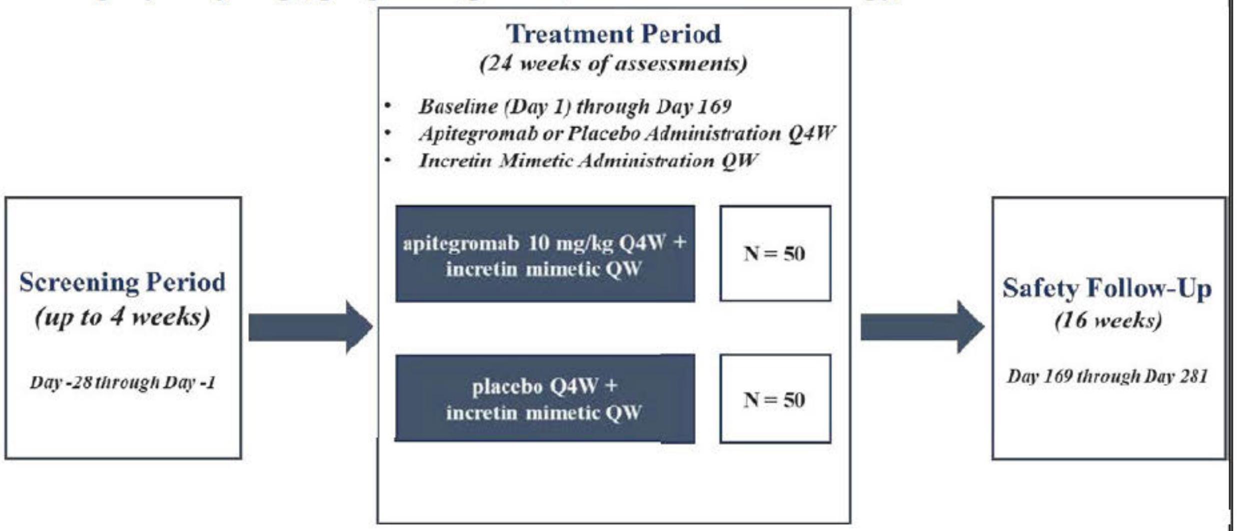

Abbreviations: N, number of subjects; Q4W, once every 4 weeks; QW, weekly.

The Schedule of Assessments (SoA) for the study is shown in Table 1. During the Screening Period, all subject Screening activities and eligibility determinations will be conducted after written informed consent has been provided and within 28 days before administration of the first dose of study drug. Assessments conducted during the study will include cardiometabolic function measurements, body weight and composition changes, assessments of a circulating biomarker and fasting glucose metabolism, PK and PD measurements, testing for antidrug antibodies (ADA)s, and safety monitoring. Dual-energy X-ray absorptiometry (DEXA), a 3-compartmental model for measuring bone mineral, fat mass, and LST (Prado 2014), will be used to evaluate body composition. Data will be reviewed on an ongoing basis in a blinded manner by the Medical Monitor(s) and the Sponsor to ensure subject safety and study integrity.

All subjects will receive the first administration of incretin mimetic therapy at the study site on Visit 1 (Day 1), at which time they will also be trained to self-administer incretin mimetic therapy QW, using the injection pen, and will receive a 1-month supply of incretin mimetic therapy. Thereafter, all subjects will receive additional 1-month supplies of incretin mimetic therapy at the Q4W visits (Visit 2 [Day 29], Visit 3 [Day 57], Visit 4 [Day 85], Visit 5 [Day 113], and Visit 6 [Day 141]).

Subjects will be randomized 1:1 in a double-blind manner to receive either apitegromab or placebo via intravenous (IV) infusion at Visits 1 through 6 at the study sites, with subject visits occurring approximately every 4 weeks through the end of the Treatment Period.

Upon completion of the Treatment Period, all subjects will enter the Safety Follow-up Period where they will be monitored for safety. During this time, subjects will not be receiving any study drug (apitegromab, placebo, or incretin mimetic therapy).

Number of subjects (planned):

Approximately 100 adults who are overweight or obese and who do not have diabetes

Study Population:

The study population will consist of obese subjects (BMI $\geq$30.0 to $\leq$45.0 kg/m^2, or subjects who are overweight (BMI $\geq$27 to <30.0 kg/m^2) with 1 or more weight-related comorbid condition(s) and meet all the inclusion criteria and none of the exclusion criteria.

Eligibility Criteria:

The main inclusion and exclusion criteria are shown below. Complete criteria for inclusion in the study are included in Section 5.1 and for exclusion from the study are included in Section 5.2.

Main Criteria for Inclusion

1. Able to comprehend the informed consent process and provide written informed consent prior to study enrollment and the conduct of any study-related assessments
2. Male or female, age ≥18 and ≤65 years old at the time of informed consent
3. Stable body weight (±5 kg) within 90 days of Screening
4. At Screening, a BMI of:
    a. ≥30.0 kg/m^2 to ≤45.0 kg/m^2 or
    b. ≥27.0 kg/m^2 to <30.0 kg/m^2 with the presence of 1 or more weight-related comorbid condition(s). Note: See exclusion criteria for specific organ class disease parameters

Main Criteria for Exclusion

1. History of or active cardiovascular, neurovascular, peripheral vascular, pulmonary, hepatic, pancreatic, neuromuscular, and/or psychiatric disease
2. Active malignancy, other than local subcutaneous squamous cell and basal cell carcinomas
3. History of immunosuppressive, chemotherapeutic, or radiation treatment within 12 months prior to Screening
4. History of Type 1 diabetes or active T2D. If there was a history of T2D and it resolved, then the resolution must have occurred >12 months prior to Screening. Prediabetes managed with nonpharmacologic approaches (lifestyle (exercise and diet) is not an exclusion

Randomization:

Subjects will be randomized 1:1 in a double-blind manner to receive apitegromab + incretin mimetic therapy or placebo + incretin mimetic therapy.

Investigational product, dosage and mode of administration:

Apitegromab is [redacted]

[redacted]

[redacted]

For the first 2 doses (Visits 1 and 2), the infusion will be administered over [redacted]. If there are no acute reactions following the first 2 doses for a subject, and if the Investigator determines that it would be safe to do so, then duration of subsequent infusions (Visits 3 to 6) may be [redacted]

Incretin mimetic therapy:

Subjects will initiate dosing of an incretin mimetic therapy (tirzepatide or semaglutide) at the lowest dose QW and should follow the recommended dose escalation schedule. The choice of the incretin mimetic will be pre-assigned by interactive web response system (IWRS). The recommended maintenance dose QW should be targeted for all subjects.

Depending on the subject's tolerability, the incretin mimetic dosing schedule may be modified as follows:

- If the subject is able to tolerate the dose according to the schedule, then dose escalation should be followed (WEGOVY USPI; ZEPBOUND USPI).
- If the subject is able to tolerate the current dose, but it is determined by the Investigator that the subject has reached the maximum tolerated dose (MTD), then the subject should be held at the current dose for up to 4 weeks. The subject will be evaluated at the next site visit for dose determination.
- If the subject is unable to tolerate the current dose, the subject should notify the site. If the subject is in between site visits, then the subject may return for an Unscheduled Visit for further evaluation by the Investigator. The Investigator may determine 1 of the following:
  - Decrease the dose to the MTD or;
  - Discontinue the incretin mimetic if the subject is not able to tolerate the decreased dose, or
  - Discontinue the incretin mimetic if the Investigator and/or the subject determine the subject is unable to tolerate both the current and decreased dose to MTD.

Duration of treatment:

Total study participation for an individual subject will consist of a Screening Period (up to 4 weeks) and a Treatment Period (24 weeks [169 days]), for a total of 28 weeks. Subjects will then enter a Safety Follow-up period of 16 weeks (112 days), during which subjects will not be receiving study drug (apitegromab, placebo, or incretin mimetic therapy). The total duration of study participation for each study subject is approximately 44 weeks.

Reference therapy, dosage and mode of administration:

The apitegromab placebo [redacted], but does not contain the active ingredient. The container closure system is identical to the apitegromab drug product.

The apitegromab placebo is administered Q4W. For the first 2 doses (Visits 1 and 2), the infusion will be administered [redacted]. If there are no acute reactions following the first 2 doses for a subject, and if the Investigator determines that it would be safe to do so, then duration of subsequent infusions (Visits 3 to 6) may be changed to [redacted]

Concomitant Medications, Therapies, and Procedures:

<u>Diet/Exercise/Counseling</u>

All subjects will receive standard care counseling with regards to lifestyle recommendations such as diet, physical activity, and behavior modification.

<u>Therapies/Procedures/Concomitant Medications</u>

Concomitant therapies or interventional procedures that are medically indicated for any adverse events (AEs) the subject has during the study or that are provided as part of standard supportive care for the subject, are permitted at the discretion of the Investigator and supersede any of the restrictions outlined in this protocol.

Criteria for Evaluation:

<u>Efficacy</u>

Efficacy will be assessed based the following measurements, including:

- Primary endpoint – DEXA scan measurements
- Secondary endpoints – body weight measurements, DEXA scan measurements

- Exploratory endpoints – DEXA scan measurements, body weight measurements, waist and height measurements, dynamometry measurements, sit-to-stand repetitions, glucose metabolism assessments, lipid panel tests, blood pressure, and adiponectin measurements

Pharmacokinetic/Pharmacodynamic

Whole blood samples will be collected to evaluate the PK of apitegromab and incretin mimetic therapy (including measurement of serum concentration) and PD of apitegromab (measurement of serum circulating latent myostatin concentration).

Immunogenicity

Immunogenicity will be evaluated through the following parameters:

- Presence or absence of ADA against apitegromab in serum from blood samples

Safety

Safety will be evaluated based on the occurrence of or changes in the following parameters:

- TEAEs, serious adverse events (SAEs), and AE of special interest (AESIs)
- Vital signs, including blood pressure, heart rate, body temperature, and respiratory rate
- Clinical safety laboratory tests (hematology, serum chemistry, coagulation, urinalysis)
- 12-lead ECGs
- Psychiatric evaluations

Adverse events will be evaluated in terms of medical severity, expectedness, relatedness, and whether they meet the regulatory definition to be considered serious.

Safety assessments may be revised if any new safety signals emerge from any ongoing Sponsor clinical studies (including this study).

Sample Size Justification:

For this descriptive proof-of-concept study, sample size is not based on providing statistical power for a formal hypothesis test. A sample size of 50 subjects per arm yields approximately 80% power to detect an effect size of 2 kg for the primary endpoint assuming a standard deviation of 4.6 and using an 80% confidence interval.

Statistical Methods:

All efficacy, safety, and PK/PD endpoints will be summarized descriptively by treatment group (ie, apitegromab + incretin mimetic therapy or placebo + incretin mimetic therapy). For the primary endpoint, the difference between treatment groups will be estimated together with a 2-sided 80% confidence interval using a linear regression model, controlling for Baseline weight, LBM, age, and sex.

Analyses are considered descriptive, and no formal hypothesis testing will be conducted. Details of planned statistical analyses and any changes to protocol-specified analyses will be delineated in the statistical analysis plan (SAP).

1.2. Schedule of Assessments

Table 1: Schedule of Assessments

Activity/Assessment	SCR	Treatment Period							Safety Follow-up Period		
**Visit**<br>**Timepoint (Study Day)**	**SCR**<br>**-28 to -1**	**V1[a]**<br>**1**	**V2**<br>**29**	**V3**<br>**57**	**V4**<br>**85**	**V5**<br>**113**	**V6**<br>**141**	**V7[b,c]**<br>**169**<br>**EOT/ET**	**V8[c]**<br>**225**	**V9[c]**<br>**281**<br>**EOS/ET**	**Unscheduled Visit[w]**
**Visit window (±days)**			±7	±7	±7	±7	±7	±7	±7	±7	
Informed consent	X[d]										
Randomization		X									
Demographics	X										
Medical history	X										
Weight management history	X										
Inclusion/Exclusion criteria	X										
Body weight[e]	X	X	X	X	X	X	X	X	X	X	X
Height	X										
BMI	X	X	X	X	X	X	X	X	X	X	X
Physical examination	X	X	X	X	X	X	X	X	X	X	X
Vital signs[f]	X	X	X	X	X	X	X	X	X	X	X
12-lead ECG[g]	X							X		X	
Waist and hip circumference		X			X			X	X	X[v]	
Baseline DEXA scan[h]<br>(D[-7] to D[-1])		X									
Post-baseline DEXA scan[i] (within ±10 days of the visit)								X	X	X[v]	
Handheld dynamometry		X						X	X	X[v]	

Activity/Assessment	SCR	Treatment Period							Safety Follow-up Period		
**Visit**<br>**Timepoint (Study Day)**	**SCR**<br>**-28 to -1**	**V1[a]**<br>**1**	**V2**<br>**29**	**V3**<br>**57**	**V4**<br>**85**	**V5**<br>**113**	**V6**<br>**141**	**V7[b,c]**<br>**169**<br>**EOT/ET**	**V8[c]**<br>**225**	**V9[c]**<br>**281**<br>**EOS/ET**	**Unscheduled Visit[w]**
**Visit window (±days)**			**±7**	**±7**	**±7**	**±7**	**±7**	**±7**	**±7**	**±7**	
Chair sit-to-stand test		X						X	X	X[v]	
Safety laboratory tests[j]	X	X	X	X	X	X	X	X	X	X	X
HbA1c	X	X			X			X	X	X[v]	
Lipid panel	X	X			X			X	X	X[v]	
C-peptide		X			X			X	X	X[v]	
Serum insulin		X			X			X	X	X[v]	
Adiponectin		X			X			X	X	X[v]	
PK and PD sampling[k]		X	X	X	X	X	X	X	X	X	X
ADA sampling[l]		X	X	X	X	X	X	X	X	X	X
Pregnancy test (if applicable)[m]	X	X	X	X	X	X	X	X	X	X	X
C-SSRS (baseline/screening version)[n]	X										
C-SSRS (since last visit version)[n]		X	X	X	X	X	X	X	X	X	
PHQ-9[n]	X	X	X	X	X	X	X	X	X	X	
Diet and physical activity counseling		X	X	X	X	X	X	X	X	X	
Site check-in telephone call		X[s]	X[s]	X[s]	X[s]	X[s]	X[s]	X[t]	X[t]		
Apitegromab or placebo dosing[o,p]		X	X	X	X	X	X				
Incretin mimetic therapy injection training and use[q,r]		X									

**Activity/Assessment**	**SCR**	**Treatment Period**							**Safety Follow-up Period**		
**Visit**<br>**Timepoint (Study Day)**	**SCR**<br>**-28 to -1**	**V1[a]**<br>**1**	**V2**<br>**29**	**V3**<br>**57**	**V4**<br>**85**	**V5**<br>**113**	**V6**<br>**141**	**V7[b,c]**<br>**169**<br>**EOT/ET**	**V8[c]**<br>**225**	**V9[c]**<br>**281**<br>**EOS/ET**	**Unscheduled Visit[w]**
**Visit window (±days)**			±7	±7	±7	±7	±7	±7	±7	±7	
Incretin mimetic therapy dispensing[r]		X	X	X	X	X	X				X[r]
Incretin mimetic therapy accountability			X	X	X	X	X	X		X[u]	X
AE recording	*To be collected from the date the ICF is signed through the last study visit.*										
SAE reporting	*To be reported from the date the ICF is signed through the last study visit.*										
Concomitant medication recording	*To be collected from the date the ICF is signed through the last study visit.*										

Abbreviations: A1c, glycated hemoglobin; Ab, antibody; ADA, antidrug antibody; AE, adverse event; BMI, body mass index; C-SSRS, Columbia Suicide Severity Rating Scale; D, study day; DEXA, dual-energy x-ray absorptiometry; ECG, electrocardiogram; EOI, end of infusion; EOS, End of Study; EOT, End of Treatment; ET, Early Termination; Hb, hemoglobin; hCG, human chorionic gonadotrophin; ICF, informed consent form; mAb, monoclonal antibody; PD, pharmacodynamics; PHQ-9, Patient Health Questionnaire – 9; PK, pharmacokinetics; SAE, serious adverse event; SCR, Screening; SOI, start of infusion; PC, telephone call; Unsch, unscheduled; V, study visit

a Baseline is Visit 1.

b Visit 7 (Day 169) serves as both the EOT/ET visit for the Treatment Period and the first visit for the Safety Follow-up Period.

c Subjects who ET prior to V7 and agree to participate in the Safety Follow-up Period visits will complete V7/EOT/ET as their ET visit and then complete V8 and V9 during the Safety Follow-up Period. Subjects who ET prior to V7 and do not agree to participate in the Safety Follow-up Period visits will complete V9/EOS/ET as the ET visit. The ET Visit should be conducted within 8 weeks of the last apitegromab or placebo dose.

d The ICF must be signed before any study-specific procedures are performed.

e Weight is collected in a fasted state at each visit before the apitegromab or placebo dose to calculate weight-based dosing.

f Vital signs (body temperature, heart rate, blood pressure, and respiratory rate) will be collected pre- and post-infusion. Subjects will remain at the study site for 1 hour for AE monitoring after each EOI but may be observed longer at the Investigator's discretion. Vital signs will also be collected prior to the subject leaving the study site (Section 9.1.2).

g 12-lead ECGs will be collected after at least 5 minutes of rest, in triplicate [3 individual tracings should be obtained no more than 2 minutes apart] (Section 9.1.4).

h The dual-energy x-ray absorptiometry (DEXA) scan at Baseline (predose) must be performed within 7 days prior to V1.

i The post-Baseline DEXA scans may be performed within ±10 days of the visit.

j Safety laboratory tests (see Table 10 and Table 11 for specific laboratory and urinalysis values/panels). Subjects should fast for at least 10 to 12 hours prior to the visit. On apitegromab or placebo dosing days, laboratory samples will be collected pre-infusion.

k Trough samples for assessment of PK and PD of apitegromab, and PK of incretin mimetic therapy will be collected on V2, V3, V4, V5, V6, and V7/EOT/ET. EOI samples for PK of apitegromab will be collected on V1, V4, and V6. PK and PD samples for apitegromab will be collected during the Safety Follow-up visits on V8, V9/EOS/ET, and Unscheduled Visit.

l Blood sample for ADA testing is collected within 1 hour before the SOI on V1, V2, V3, V4, V5, and V6 dosing days. ADA samples will also be collected during the Safety Follow-up visits on V8, V9/EOS/ET, and Unscheduled Visit.

m Females of childbearing potential only (Section 9.1.7). Serum testing will be used at Screening. Urine test must be performed during on-site visits before apitegromab or placebo dosing; however, positive urine tests must be confirmed with serum testing. If a positive urine pregnancy test occurs, an unscheduled kit will be used for a serum pregnancy test. Testing may be performed more frequently as per local requirements.

n The C-SSRS and PHQ-9 should be administered after assessment of AEs.

o Dosing of apitegromab or placebo every 4 weeks should be targeted. There is a ±7-day window around each dosing visit, with a minimum of 21 days and a maximum of 35 days between apitegromab or placebo doses. If dosing cannot be performed within the ±7-day window, this divergence from the targeted dosing (every 4 weeks) will be considered a protocol deviation and missed visit.

p For the first 2 doses (V1 and V2), the infusion will be administered over [redacted] If there are no acute reactions following the first 2 doses for a subject, and if the Investigator determines that it would be safe to do so, then duration of subsequent infusions (V3 to V6) may be changed to [redacted]

q Prior to initiation of incretin mimetic therapy home administration, subjects will be trained by qualified site staff on proper injection technique. The first dose of incretin mimetic therapy will be administered by the subject at V1 while being monitored by site staff to ensure proper technique.

r Incretin mimetic therapy is supplied in cartons containing 4 injection pens—enough for 4 weekly injections. Subjects may also request an Unscheduled Visit for additional training on the self-administration procedure for incretin mimetic therapy.

s Sites will contact subjects by telephone within 7 days after each dosing visit to collect information on adverse events and concomitant medications. Subjects will also be asked if they have any questions regarding the self-administration procedure for incretin mimetic therapy.

t Sites will contact subjects by telephone within 28 days after each safety follow-up visit to check for adverse events and concomitant medications.

u Applicable only if subject is ET before V7/EOT is complete.

v If an Early Termination occurs following completion of V7/EOT but before V8 is completed, then the V9/ET assessments should be performed. If a subject completes V8, certain V9 assessments do not need to be performed again during V9, as delineated by the footnote.

w Procedures that can be performed at an Unscheduled Visit are defined. The Investigator can decide which of the remaining assessments (any and/or all) need to be performed at each Unscheduled Visit.

2. INTRODUCTION

Apitegromab is an investigational, fully human immunoglobulin G (IgG) 4 monoclonal antibody (mAb) that specifically binds with high affinity to promyostatin and latent myostatin, the inactive precursor forms of myostatin, a growth factor of the TGF-β superfamily that is expressed predominantly in skeletal muscle and functions to restrict skeletal muscle size. Myostatin is initially produced in an inactive form, and 2 sequential proteolytic steps are required to release the active growth factor form of myostatin, which signals locally within skeletal muscle. Approaches which inhibit myostatin signaling are therefore expected to result in increased skeletal muscle mass and potential increases in strength. Apitegromab binds to both promyostatin and latent myostatin and prevents the release of the mature myostatin growth factor in skeletal muscle.

Apitegromab is being developed as an adjunctive therapy to incretin mimetic therapy for the treatment of adults who are overweight or obese.

2.1. Study Rationale

This is a Phase 2a randomized, double-blind, placebo-controlled, multicenter study targeting enrollment of approximately 100 adults with overweight or obesity without diabetes (WHO 2023). The study will assess the safety, efficacy, and pharmacokinetics (PK) of apitegromab when used as an adjunctive therapy to incretin mimetic therapy.

Adjunctive pharmacotherapy for weight loss is indicated for individuals with a BMI ≥30 kg/m^2 or ≥27 kg/m^2 in persons with 1 or more weight-related comorbid condition(s), who have not met weight loss goals with a lifestyle intervention approach (Acosta 2017, Jensen 2014). A growing number of incretin mimetic therapies are being developed for weight loss in patients with overweight or obesity and without Type-2 diabetes (T2D). Incretin mimetics, including glucagon-like peptide 1 (GLP-1) receptor agonists (RAs) and GLP-1 receptor and glucose dependent insulinotropic polypeptide (GIP) dual agonists, have been shown to lower body weight through decreased caloric intake, most likely through appetite suppression. Clinical use of semaglutide, a GLP-1 RA for obesity treatment, has led to robust improvements in weight loss (Bergmann 2023) and improvements in cardiometabolic risk factors and quality of life; however, common side effects include delayed gastric emptying, nausea, vomiting, and diarrhea (Joshi 2023, Klein 2023, Kobori 2023, Silveira 2023). Real-world evidence demonstrates sub-optimal adherence and/or discontinuation of GLP-1 RA therapy (Ghusn 2022, Weiss 2020). Tirzepatide, a GLP-1/GIP dual receptor agonist approved for obesity treatment, has also been shown to provide substantial and sustained reductions in body weight in patients with overweight or obesity (Jastreboff 2022), with similar common side effects.

Rapid reversal of weight loss and cardiometabolic benefits has been observed following withdrawal of semaglutide (Rubino 2021, Wilding 2022) and tirzepatide (Aronne 2024) as well as other obesity therapeutics such as orlistat (Sjöström 1998, Smith 2010) despite continued lifestyle intervention. These observations highlight the chronic nature of obesity and the significant unmet need for improved tolerability and durability of pharmacological interventions.

Both dynamic weight loss and attempts to maintain a reduced weight are associated with a disproportionate decline in energy expenditure beyond that predicted solely on the basis of

changes in body weight and composition; this decline in energy expenditure may contribute to weight regain. Importantly, a substantial portion (20% to 50%; Sargeant 2019) of total body weight loss (TBWL) mediated by incretin mimetic therapy may be attributed to loss of lean body mass (LBM), which is consistent with diet induced weight loss and bariatric surgery (Ida 2021, Sargeant 2019, Wilding 2021). Skeletal muscle makes up the largest portion of the LBM, which does not include bone (Prado 2014, Yu 2013). Increases in LBM have been shown to improve basal metabolic rate (BMR) (Aristizabal 2015), improve insulin sensitivity (Lindegaard 2008, Srikanthan 2011), reduce visceral adipose tissue [VAT] (Wewege 2022), and increase caloric expenditure postexercise (Zurlo 1990). Therefore, preservation of LBM during and after weight loss may be beneficial. Myostatin, a member of the transforming growth factor beta (TGF-β) superfamily of growth factors, is expressed primarily by skeletal muscle cells; the myostatin pathway is highly conserved from animals to humans. Myostatin is a secreted protein that negatively regulates skeletal muscle mass and function (Day 2022, Long 2019). Bimagrumab, an investigational monoclonal antibody (mAb) that inhibits myostatin via activin receptor II (ActRII) blockade, was evaluated in a Phase 2 study in adult overweight and obese subjects with T2D. Treatment with bimagrumab led to a gain in LBM, reduction in fat mass, and improvements in glycemic parameters (Heymsfield 2021). Therefore, combining inhibition of myostatin activation with incretin mimetic-driven weight loss may address the loss of LBM in this setting and consequently avoid the decline in energy expenditure associated with loss of lean muscle mass.

Apitegromab is a fully human IgG4 mAb that specifically binds with high affinity to both promyostatin and latent myostatin and prevents the release of the mature myostatin growth factor in skeletal muscle. Apitegromab's high selectivity for latent myostatin, in addition to the lack of effect on signaling through other endogenous-TGF-β family members, is distinct from bimagrumab. Apitegromab is administered via intravenous (IV) infusion. Nonclinical studies demonstrated that selective inhibition of myostatin with apitegromab or its analog was associated with an increase in LBM in the mouse, rat, and cynomolgus monkey (Apitegromab CM Obesity IB, Long 2019). Clinical administration of apitegromab in pediatric and adult subjects with spinal muscular atrophy (SMA) was associated with improved motor function (as measured by Hammersmith Functional Motor Scale – Expanded [HFMSE]) and reduced fatigue (as measured by the Patient Reported Outcome Measurement Information System [PROMIS] Fatigue Scale (Day 2022). Overall, the tolerability profile of apitegromab is favorable, with no identified safety risks.

This study will be evaluating the effects of apitegromab when used as an adjunctive therapy to incretin mimetic therapy in subjects with overweight or obesity and without diabetes. Taken together, the Sponsor believes that apitegromab in the setting of incretin mimetic-driven weight loss has the potential to attenuate the loss of LBM associated with incretin mimetic therapy alone, thus improving body composition and mitigating the reductions in metabolic rate, which in turn could further enhance fat loss.

2.2. Background

2.2.1. Obesity

The Obesity Medicine Association defines obesity as a chronic, progressive, relapsing, and treatable multi-factorial, neurobehavioral disease, wherein an increase in body fat promotes

adipose tissue dysfunction and abnormal fat mass physical forces, resulting in adverse metabolic, biomechanical, and psychosocial health consequences (OMA 2021). Adipose tissue is a metabolically active endocrine organ (Coelho 2013) that has complex systemic effects and is linked to as many as 236 comorbid conditions including 13 types of cancer (Ligibel 2014), T2D, hypertension, hyperlipidemia, sleep apnea, depression, and reduction in life span by up to 8 years (Garvey 2009, Rubino 2021).

2.2.2. Adiposity Assessment

Most commonly, adiposity is measured with general and central obesity anthropometric measurements including body mass index (BMI); weight in kilograms (kg) divided by square of height in meters (m), waist circumference (WC), hip circumference (HC), waist-to-height ratio (WHtR), and waist-to-hip ratio [WHR] (Goh 2014). BMI is a calculation that estimates a person's body fat using their height and weight. While BMI assessment is a simple, inexpensive, and noninvasive surrogate measure of body fat, variables such as age, sex, ethnicity, and muscle mass, have an influence on the relationship between BMI and body fat. BMI value does not indicate the relative proportion of excess fat, muscle, or bone mass, nor does BMI provide any indication of the distribution of fat among individuals. BMI may be particularly unreliable for the following groups, individuals with increased muscle mass such as athletes, individuals with increased third space volume such as with ascites, heart and renal failure, or individuals with decreased muscle mass such as the elderly (Romero-Corral 2008).

Dual-energy X-ray absorptiometry (DEXA) is a quick, noninvasive, low dose ionizing imaging modality that is safe for repeated measurements. DEXA is a dynamic imaging tool that can measure 3 body composition compartments: bone, fat, and LBM, which is the sum of body water, total body protein, carbohydrates, nonfat lipids, and soft tissue minerals. (Shepherd 2017, Prado 2014). Within this document, LBM is synonymous with lean soft tissue (LST), which is the sum of body water, total body protein, carbohydrates, nonfat lipids and soft tissue mineral (skeletal muscle, organs and connective tissue). Skeletal muscle makes up the largest portion of the LBM, which does not include bone (Prado 2014, Yu 2013).

2.2.3. Weight Loss and Lean Body Mass Loss

Considerable evidence indicates that successful treatment of obesity results in a reduced incidence of comorbid diseases. Specifically, a 5% to 10% total weight loss (TWL) can significantly reduce or prevent many chronic diseases in overweight and obese individuals (NIH 1998). Although TWL in overweight and obese individuals has major health benefits, none of the current weight loss treatments including diet, bariatric surgery, Food and Drug Administration (FDA) approved anti-obesity devices and medications specifically target fat mass and/or preserve or increase LBM. Importantly, a substantial portion (20% to 50%) (Sargeant 2019) of TBWL mediated by incretin mimetic therapy may be attributed to loss of LBM (which is consistent with diet induced weight loss and bariatric surgery) (Ida 2021, Sargeant 2019, Wilding 2021). The preservation of LBM has many benefits for overall health and well-being, including an increased BMR (Aristizabal 2015), enhanced glucose homeostasis (Lindegaard 2008, Srikanthan 2011), reduced VAT (Wewege 2022), increased caloric expenditure postexercise (Zurlo 1990), increased bone density, strength, function, and longevity and decreased risk of injury, and disability (Fukushima 2016, Roh 2020, Volpi 2004). Efforts to preserve LST becomes more important with age since there is an involuntary loss of muscle

around 3% to 8% per decade after the age of 30. This rate of decline increases between 60 to 70 years of age where the prevalence of sarcopenia is between 5% to 13%. By age 70, 25% to 30% of skeletal muscle mass is lost and there is a 40% decline in strength (Casati 2019, Volpi 2004).

2.2.4. Overweight and Obesity

The Centers for Disease Control (CDC 2023) categorizes individuals based on their BMI. The BMI categories and the corresponding weight classifications are listed in Table 2.

Table 2: Body Mass Index and Obesity Classifications

<table>
<tr><th>Classification</th><th colspan="2">Body Mass Index Range</th></tr>
<tr><td>Underweight</td><td colspan="2"><18.5 kg/m^2</td></tr>
<tr><td>Healthy weight</td><td colspan="2">18.5 to <25 kg/m^2</td></tr>
<tr><td>Overweight</td><td colspan="2">25 to <30 kg/m^2</td></tr>
<tr><td rowspan="4">Obesity</td><td colspan="2">≥ 30 kg/m^2</td></tr>
<tr><td>Class 1</td><td>30 to <35 kg/m^2</td></tr>
<tr><td>Class 2</td><td>35 to <40 kg/m^2</td></tr>
<tr><td>Class 3 (severe obesity)</td><td>≥ 40 kg/m^2</td></tr>
</table>

2.2.5. Current Therapies

There are several categories of therapies available for the treatment of overweight and obesity. These include lifestyle changes such as diet, physical activity, and behavior modification. Lifestyle changes are the first line of treatment and an important component of the other therapeutic categories that include weight loss medications, weight loss devices, and bariatric surgery. However, as mentioned above, the benefits of TBWL are often compromised by the loss of LBM, which happens with all aforementioned therapies. LBM loss contributes to decreased energy expenditure and the loss of counterregulatory hormones, which can lead to weight rebound, worsening glucose homeostasis, and other health problems (Friedl 2000, Harman 2004).

2.2.6. Apitegromab

Apitegromab specifically binds with high affinity to both promyostatin and latent myostatin and prevents the release of the mature myostatin growth factor in skeletal muscle. Apitegromab's high selectivity for latent myostatin, in addition to the lack of effect on signaling through other endogenous-TGF-β family members, is distinct from bimagrumab. Apitegromab is administered via intravenous (IV) infusion. Preclinically, selective inhibition of myostatin with apitegromab or its analog was associated with an increase in lean muscle mass in the mouse, rat, and cynomolgus monkey (Long 2019).

Clinical administration of apitegromab in pediatric and adult patients with SMA was associated with improved motor function and reduced fatigue (Day 2022, SRK-015-002 [TOPAZ] Interim CSR, Crawford 2023). Overall, the tolerability profile of apitegromab is favorable, with no identified safety risks as described in Section 2.2.6.3 and Section 2.2.6.4. Apitegromab administration increased muscle mass and strength in healthy mice and rats, mouse models of

SMA, and increased muscle mass in healthy monkeys compared with vehicle-treated animals. Furthermore, apitegromab administration in SMA patients have demonstrated substantial and sustained improvement in motor function.

This study will be evaluating the effects of apitegromab when used as an adjunctive therapy to incretin mimetic therapy in subjects who are overweight and obese and who do not have diabetes. Taken together, the Sponsor believes that apitegromab in the setting of incretin mimetic-driven weight loss has the potential to attenuate the loss of LBM associated with incretin mimetic therapy alone, thus improving body composition and mitigating the reductions in metabolic rate, which in turn could further enhance fat loss.

2.2.6.1. Mechanism of Action

Data from the nonclinical program demonstrate that apitegromab specifically binds the precursor forms of myostatin and prevents activation and release of the mature growth factor. Apitegromab does not bind the mature myostatin growth factor and, unlike the majority of other myostatin inhibitors, does not bind any form of growth differentiation factor 11 (GDF11), or Activin A, or the mature forms of bone morphogenetic protein (BMP) 9/10 or transforming growth factor-β1. The highly specific property of apitegromab has the potential to minimize off-target effects associated with less selective myostatin-directed agents. The mechanism of action for apitegromab is shown in Figure 1.

Figure 1: Apitegromab Mechanism of Action

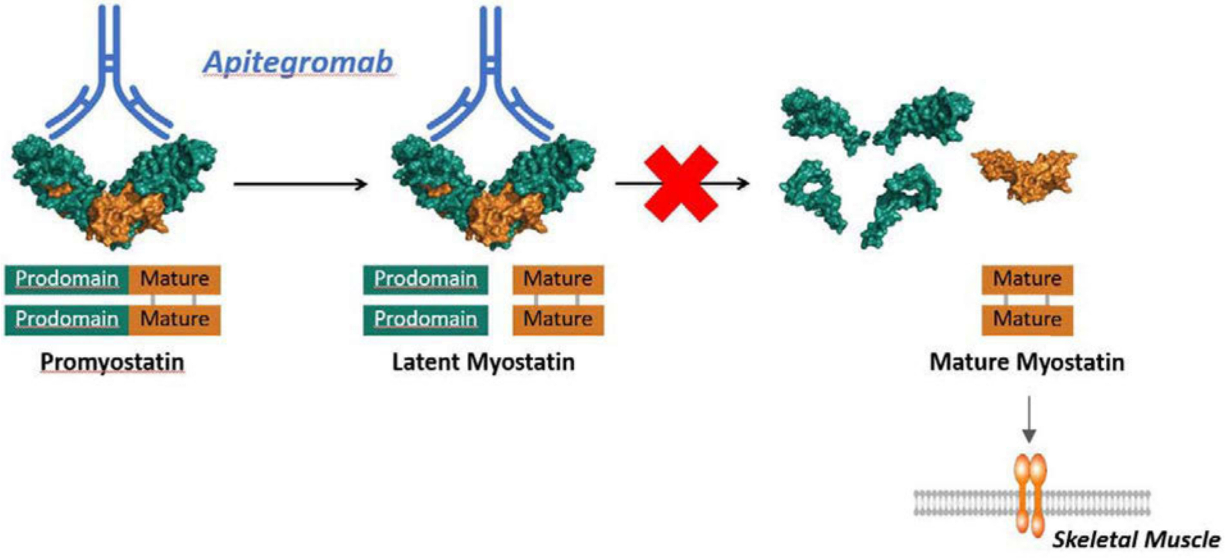

Myostatin is found in a latent and inactive form, with 2 proteolytic steps necessary for release of mature myostatin, which is the active form. Apitegromab binds to both pro-myostatin and latent myostatin and blocks the conversion of the latent form to mature myostatin. By inhibiting the release of mature myostatin, apitegromab prevents myostatin from interacting with receptors on skeletal muscle, thereby enabling muscle growth.
Source: Adapted from Long 2019 and Pirruccello-Straub 2018.

Please refer to Section 2.4.1 of the Apitegromab Investigator's Brochure for CM Obesity for more detailed information on the mechanism of action.

2.2.6.2. Nonclinical Pharmacology

The ability of apitegromab to increase muscle mass and strength was demonstrated in nonclinical pharmacology studies in healthy animals as well as in multiple rodent models of muscle atrophy, including 2 models of SMA. Apitegromab also significantly increased muscle mass in nonhuman primates. Notably, in nonhuman primates, apitegromab administration led to increases in the mass of muscles rich with fast-twitch fibers. Together, these results demonstrate the potential of apitegromab in treating diseases of muscle atrophy, especially those associated with reduced fast-twitch fiber size such as SMA. Refer to Section 4.0 of the apitegromab Investigator's Brochure for CM Obesity for more detailed information on the nonclinical studies.

Activity of apitegromab, using an ex vivo functional assay measuring inhibition of proteolytic activation of promyostatin or latent myostatin, is similar across all nonclinical and toxicologically relevant species (Scholar Rock Studies SRP015-17-04, RPT-00120, and RPT-00242). Results from these in vitro studies demonstrate that apitegromab specifically targets myostatin via inhibition of proteolytic activation of precursor forms of myostatin in all 5 species [human, cynomolgus monkey, rabbit, rat, mouse] (Pirrucello-Straub 2018) (Scholar Rock Studies SRP015-17-04, RPT-00120, and RPT-00242). Apitegromab does not bind the mature myostatin growth factor and does not bind any form of GDF11 or Activin A, or the mature forms of BMP9/10 or TGFβ1. Data from cryosections of tibialis anterior muscles from healthy mice indicate that the inactive precursor forms of myostatin are present in the extracellular space in skeletal muscle and therefore accessible for binding and inhibition by apitegromab (Pirrucello-Straub 2018).

2.2.6.3. Summary of Safety of Apitegromab in SMA

As of 24 April 2023, no deaths or serious adverse reactions (SARs) have been identified in clinical studies with apitegromab. No adverse drug reactions (ADRs) or safety signals have been identified for apitegromab. Refer to the apitegromab Investigator's Brochure for CM Obesity for the full safety information.

2.2.6.4. Summary of Efficacy of Apitegromab in SMA

Efficacy data from the 12-month Treatment Period analysis in Study SRK-015-002 (TOPAZ) support the clinical effect of apitegromab on motor function in subjects with Type 2 and Type 3 SMA.

Although each cohort in Study SRK-015-002 (TOPAZ) essentially represents an independent study with a different population and a different clinical setting, the results across the cohorts and analysis time points are consistent and mutually supportive.

At 12 months of apitegromab treatment, the majority of subjects - independent of age, SMA type, or time of SMN therapy initiation - had stable or improved motor function, as measured by the RHS or HFMSE scores. These findings suggest that the observed clinical benefit is due to apitegromab treatment given that patients in this study had received, on average, 24 months of nusinersen therapy, which is already the plateau phase of effectiveness from nusinersen treatment (Mercuri 2020). The observed dose response results further support the clinical benefit of apitegromab treatment. Furthermore, improvements in patient reported outcomes, as measured by PEDI-CAT and PROMIS Fatigue instruments, in the combined Type 2 and Type 3 SMA patients were consistent with the gains in the motor function scores and clinical benefit.

2.3. Benefit/Risk Assessment for Apitegromab

2.3.1. Benefit Assessment

All subjects in this study will receive an incretin mimetic therapy (tirzepatide or semaglutide) approved for chronic weight management in subjects meeting Inclusion Criteria 4 and 5 listed in Section 5.1.

Approximately 50% of subjects will also receive the investigational muscle-directed therapy apitegromab, at 10 mg/kg (one-third of the high dose and 5 times the low dose in Study SRK-015-001). Apitegromab, which is directed toward attenuating the loss of LBM, offers a mechanism of action (Section 2.2.6.1) and biological effect hypothesized to offer additional benefits to those of currently approved chronic weight management therapies. In vivo nonclinical data support that addition of apitegromab may increase muscle mass and strength (Section 2.2.6.2). In clinical studies, subjects with SMA treated with apitegromab in Study SRK-015-002 (TOPAZ) (Section 2.2.6.4) and SRK-015-003 (SAPPHIRE), demonstrated improved motor function through validated outcome measures (ie, HFMSE and Revised Upper Limb Module [RULM]. Additionally, through specific inhibition of mature myostatin's release from its inactive precursor, apitegromab has the potential to minimize off-target effects that have been implicated as the cause of adverse effects of other less selective myostatin-directed agents (Campbell 2017, Garito 2018).

These data support the clinical use of apitegromab in subjects at risk of LBM loss with incretin mimetic therapy, who may derive benefit due to potential retention or increase in lean body mass.

All subjects will receive medical evaluations and assessments (eg, physical examinations, electrocardiograms [ECGs], and blood tests) throughout the clinical study.

2.3.2. Risk Assessment

In the nonclinical toxicology program for apitegromab, no significant adverse findings have been observed across various studies. These include 4-week studies in adult rodents and nonhuman primates with doses up to 100 mg/kg, a 7-week study in juvenile rodents at doses up to 300 mg/kg, and 12-week and 26-week studies in adult rodents at doses up to 300 mg/kg. Additionally, a fertility and early embryonic development study was conducted in adult rodents at up to 300 mg/kg, covering phases from premating to early gestation. Two embryo-fetal development studies were also completed, one each in rabbits and adult rodents, with doses up to 300 mg/kg, focusing on critical gestation periods.

In Study SRK-015-001 with healthy subjects, and subsequent studies SRK-015-002 (TOPAZ), SRK-015-003 (SAPPHIRE), and SRK-015-004 involving subjects with SMA, no safety risks were identified. These results, covering a period up to 24 April 2023, include a 36-month primary analysis period in subjects with later-onset SMA. The incidence and severity of adverse events (AEs) observed were in line with what is expected in the SMA patient population and those undergoing background therapy with nusinersen or risdiplam. Overall, in all the studies involving apitegromab, consistent findings indicate a favorable safety profile for the drug. This consistency across various subject groups and study durations underscores the safety of apitegromab in both healthy individuals and those with SMA.

According to the United States Prescribing Information for semaglutide, a comprehensive range of risks associated with the use of semaglutide has been identified and documented under the warnings and precautions section. These risks include acute pancreatitis, acute gallbladder disease, hypoglycemia, acute kidney injury and hypersensitivity reactions have been noted. Patients with T2D may also experience complications related to diabetic retinopathy. Other risks highlighted are an increased heart rate, suicidal behavior and ideation, and a risk of developing thyroid C-cell tumors. Similar risks associated with the use of tirzepatide have been identified and documented, with 2 notable differences: heart rate increase is not an identified and documented risk of tirzepatide, while an associated risk of severe gastrointestinal disease has been identified and documented for this drug.

Drug interactions highlighted are that tirzepatide and semaglutide delay gastric emptying and may impact absorption of concomitantly administered oral medications. However, no clinically relevant drug-drug interaction between semaglutide and oral medications has been reported. Prescribers of tirzepatide are guided to monitor patients on oral medications dependent on threshold concentrations for efficacy and those with a narrow therapeutic index (eg, warfarin) when concomitantly administered with tirzepatide, and to advise patients using oral hormonal contraceptives to switch to a non-oral contraceptive method, or add a barrier method of contraception, for 4 weeks after initiation with tirzepatide and for 4 weeks after each dose escalation. Hormonal contraceptives that are not administered orally should not be affected by tirzepatide.

Apitegromab is not a cytokine modulator, and therefore is not expected to have any modulatory effects on metabolism of drugs that are metabolized by the cytochrome P450 enzymes. The potential for a drug-drug interaction between apitegromab and tirzepatide or semaglutide is low.

2.3.3. Overall Benefit/Risk Conclusion

In vivo nonclinical data support that addition of apitegromab may increase muscle mass and strength (Section 2.2.6.2). Clinical studies in subjects with SMA treated with apitegromab in Study SRK-015-002 (TOPAZ) (Section 2.2.6.4) and SRK-015-003 (SAPPHIRE) support the clinical efficacy of apitegromab to improve motor function. Taken together, these data support that overweight and obese subjects at risk of lean muscle loss due to incretin mimetic therapy for weight loss may potentially derive benefit from retention or increase of lean muscle mass. It is hypothesized that subjects treated with apitegromab (versus placebo) may experience increased durability of weight loss and cardiometabolic benefits from incretin-mediated therapy. As no safety risks for apitegromab have been identified to date in the clinical development program and no adverse toxicology findings have been observed to date in the nonclinical program, the potential risks identified in association with apitegromab are justified by the potential benefits that may be afforded to overweight and obese subjects.

Tirzepatide and semaglutide have shown significant efficacy in chronic weight management in the adult population with obesity, offering benefits such as weight reduction, improved glycemic control, and better cardiovascular health outcomes. These advantages are particularly valuable in patients who have not achieved desired results with conventional treatment methods. However, tirzepatide and semaglutide also have risks such as acute pancreatitis, acute gallbladder disease, hypoglycemia, acute kidney injury, hypersensitivity reactions, and complications related to

diabetic retinopathy. There is also an increased risk of heart rate elevation (semaglutide only) as well as suicidal behavior and ideation and thyroid C-cell tumors.

In conclusion, while tirzepatide and semaglutide present benefits in weight management in patients with overweight or obesity and improving blood sugar levels in patients with T2D, use of these drugs must be carefully evaluated against the potential risks. Overall, the benefits-risks balance for tirzepatide and semaglutide is considered favorable.

3. STUDY OBJECTIVES AND PURPOSE

The study objectives and endpoints are shown in Table 3.

Table 3: Study Objectives and Endpoints

Objectives	Endpoints
**Primary**	
• Evaluate the efficacy of apitegromab versus placebo when used as an adjunctive therapy to incretin mimetic therapy in subjects with overweight or obesity and without diabetes	• Change from Baseline at 24 weeks in LBM
**Secondary**	
• Evaluate the effect of apitegromab versus placebo on body weight when used as an adjunctive therapy to incretin mimetic therapy in subjects with overweight or obesity and without diabetes	• Change from Baseline at 24 weeks in body weight
• Evaluate the effect of apitegromab versus placebo on body composition when used as an adjunctive therapy to incretin mimetic therapy in subjects with overweight or obesity and without diabetes	• Change from Baseline at 24 weeks in DEXA measurements, including:<br>○ Percent LBM<br>○ Total and percent of fat body mass<br>○ Total and percent of VAT, SAT, and trunk fat body mass<br>○ Percent of weight loss from Baseline due to fat body mass loss or LBM loss
• Evaluate the PK and PD of apitegromab when used as an adjunctive therapy to incretin mimetic therapy in subjects with overweight or obesity and without diabetes	• Trough and EOI concentration of apitegromab through 24 weeks of treatment<br>• Trough concentrations of latent myostatin through 24 weeks of treatment<br>• Concentration of apitegromab and latent myostatin during Safety Follow-up Period (Week 25 through Week 40)
• Evaluate the safety and tolerability of apitegromab when used as an adjunctive therapy to incretin mimetic therapy in subjects with overweight or obesity and without diabetes	• Frequency of TEAEs and SAEs by severity<br>• Change from Baseline in clinical safety laboratory tests, vital signs, ECG measurements, and psychiatric evaluations<br>• Presence or absence of ADAs against apitegromab from serum blood samples

**Objectives**	**Endpoints**
**Exploratory**	
• Evaluate exploratory efficacy parameters of apitegromab versus placebo when used as an adjunctive therapy to incretin mimetic therapy in subjects with overweight or obesity and without diabetes	• Change from Baseline at 32 weeks in DEXA measurements:<br>○ Proportion of subjects with categorical percent changes from Baseline in body weight, fat body mass, and/or LBM<br>• Proportion of subjects with categorical changes from Baseline in waist circumference<br>• Proportion of subjects with change from Baseline in WHtR and WHR categories<br>• Change from Baseline in force production assessed by handheld dynamometry<br>• Change from Baseline in the number of sit-to-stand repetitions in the chair sit-to-stand test
• Evaluate effect of apitegromab versus placebo on glucose metabolism, lipid metabolism, and systolic and diastolic blood pressure when used as an adjunctive therapy to incretin mimetic therapy in subjects with overweight or obesity and without diabetes	• Change from Baseline in glucose metabolism assessments, including plasma glucose, HbA1c, serum insulin, C-peptide, and HOMA-IR values<br>• Change from Baseline in lipid panel tests including total cholesterol, triglycerides, LDL, HDL, and VLDL<br>• Change from Baseline in systolic and diastolic blood pressure
• Evaluate effect of apitegromab versus placebo on an exploratory biomarker when apitegromab is used as an adjunctive therapy to incretin mimetic therapy in subjects with overweight or obesity and without diabetes	• Change from Baseline in adiponectin values
• Evaluate the PK of incretin mimetic therapy in the presence and absence of apitegromab in subjects with overweight or obesity and without diabetes	• Concentration of incretin mimetic therapy through 24 weeks of treatment

Abbreviations: A1c, glycated hemoglobin; ADA, antidrug antibody; DEXA, dual-energy x-ray absorptiometry; ECG, electrocardiogram; EOI, end of infusion; Hb, hemoglobin; LBM, lean body mass; PD, pharmacodynamics; PK, pharmacokinetics; SAE, serious adverse event; SAT, subcutaneous adipose tissue; TEAE, treatment-emergent adverse events; VAT, visceral adipose tissue; VLDL, very low-density lipoprotein; WHR, waist-to-hip ratio; WHtR, waist-to-height ratio.

4. INVESTIGATIONAL PLAN

4.1. Overall Study Design

This Phase 2a randomized, double-blind, placebo-controlled, multicenter study will be conducted to evaluate the efficacy, safety, and PK of apitegromab in adult subjects with overweight or obesity and without diabetes at approximately 20 study sites in the United States (US). Approximately 100 adult subjects with overweight or obesity and without diabetes will be randomized to receive apitegromab 10 mg/kg Q4W + incretin mimetic therapy once weekly (QW) or placebo Q4W + incretin mimetic therapy QW. Administration of incretin mimetic therapy will be based on a dose escalation schedule, beginning at the lowest dose and escalating Q4W to the next dose until a recommended maximum tolerated maintenance dose has been reached.

As shown in Figure 2, the study will include a Screening Period (up to 4 weeks), a Treatment Period, and a Safety Follow-up Period. Subjects completing the 24-week (169-day) Treatment Period will be followed for 16 weeks (112 days) in the Safety Follow-up Period, during which time they will not be receiving any study drug (apitegromab, placebo, or incretin mimetic therapy).

Figure 2: Overall Study Design

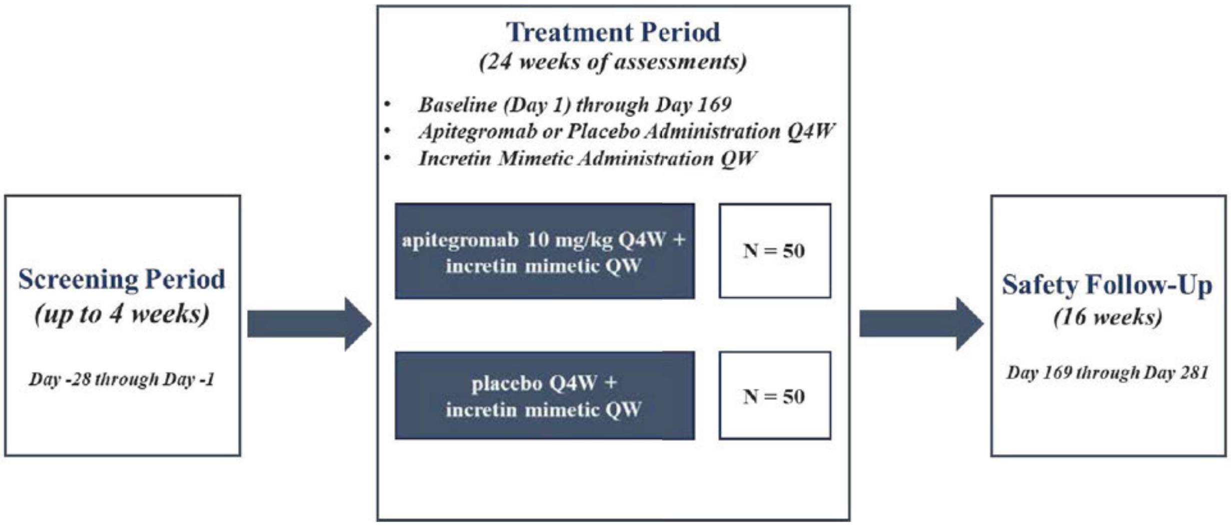

Abbreviations: N, number of subjects; Q4W, once every 4 weeks; QW, weekly.

The Schedule of Assessments (SoA) for the study is shown in Table 1. During the Screening Period, all subject Screening activities and eligibility determinations will be conducted after written informed consent has been provided and within 28 days before administration of the first dose of study drug. Assessments conducted during the study will include cardiometabolic function measurements, body weight and composition changes, assessments of a circulating biomarker and fasting glucose metabolism, PK and PD measurements, testing for antidrug antibodies, and safety monitoring. DEXA, a 3-compartmental model for measuring bone mineral, fat mass, and lean soft tissue (Prado 2014), will be used to evaluate body composition.

Data will be reviewed on an ongoing basis in a blinded manner by the Medical Monitor(s) and the Sponsor to ensure subject safety and study integrity.

All subjects will receive the first administration of an incretin mimetic therapy at the study site on Visit 1 (Day 1), at which time they will also be trained to self-administer the incretin mimetic therapy QW, using the injection pen, and will receive a 1-month supply of incretin mimetic therapy. Thereafter, all subjects will receive additional 1-month supplies of incretin mimetic therapy at the Q4W dosing visits (Visit 2 [Day 29], Visit 3 [Day 57], Visit 4 [Day 85], Visit 5 [Day 113], and Visit 6 [Day 141]).

Subjects will be randomized 1:1 in a double-blind manner to receive either apitegromab or placebo via intravenous (IV) infusion at Visits 1 through 6 at the study sites, with these dosing visits occurring approximately every 4 weeks through the end of the Treatment Period.

Upon completion of the Treatment Period, all subjects will enter the Safety Follow-up Period where they will be monitored for safety. During this time, subjects will not receive any study drug (apitegromab, placebo, or incretin mimetic therapy).

4.2. Scientific Rationale for Study Design

This study will be evaluating the effects of apitegromab when used as an adjunctive therapy to incretin mimetic therapy in subjects who are overweight and obese and who do not have diabetes. Taken together, the Sponsor believes that apitegromab in the setting of incretin mimetic-driven weight loss has the potential to attenuate the loss of LBM associated with incretin mimetic therapy alone, thus improving body composition and mitigating the reductions in metabolic rate, which in turn could further enhance fat loss.

All subjects will receive incretin mimetic therapy at a dose regimen escalating to the maximum tolerated maintenance dose QW, while approximately one-half of the subjects will receive apitegromab 10 mg/kg Q4W and the remaining one-half of the subjects will receive placebo. The randomized double-blind placebo-controlled study design is the best practice to eliminate the influence of unknown or immeasurable confounding variables that may otherwise lead to biased and incorrect estimate of treatment effect of apitegromab. The use of a placebo group to scientifically confirm the efficacy and safety of a study drug is common practice when subjects are on active background therapy.

4.3. Number of Subjects

Approximately 100 subjects who are overweight or obese and who do not have diabetes will be included in this study.

4.4. Treatment Assignment

Subjects will be randomized to receive apitegromab 10 mg/kg Q4W + incretin mimetic therapy QW or placebo Q4W + incretin mimetic therapy QW. Administration of the incretin mimetic therapy will be based on a dose escalation schedule, beginning at the lowest dose and escalating Q4W to the next dose until a maximum tolerated maintenance dose has been reached.

Subjects will receive either an incretin mimetic therapy (either tirzepatide or semaglutide - the choice will be preassigned) and will remain on the same incretin mimetic therapy throughout the treatment period, per interactive web response system (IWRS) assignment.

4.5. Dosing

4.5.1. Dose Justification

4.5.1.1. Apitegromab

The 10 mg/kg dose of apitegromab was selected based on a consideration of PK/PD data from the first in human study in healthy adults (SRK-015-001) as well efficacy data from the Phase 2 study in SMA (SRK-015-002 [TOPAZ]).

Apitegromab was tested in a SAD and MAD adult healthy volunteer clinical study (Study SRK-015-001), as well as a multi-year SMA patient study (Study SRK-015-002 [TOPAZ]; age range 2 through 21 years) (Section 2.2.6.3).

In Study SRK-015-001, the first in human study in healthy volunteers, single and multiple doses of up to 30 mg/kg of apitegromab were administered and were generally safe and well tolerated. Pharmacokinetic results suggest that apitegromab has a profile generally consistent with mAbs where dose-proportional increase in exposure, as well as low variability, was observed. The serum half-life ranged from 23 to 33 days across the apitegromab dose groups. These PK characteristics of apitegromab supported the investigation of a once every 4-week dosing regimen in Study SRK-015-002 (TOPAZ).

The population PK of apitegromab in healthy adults was described by a 2-compartment model with linear elimination, parameterized using clearance (central and intercompartmental), and volume of distribution (central and peripheral). The median (min-max) of body weight in subjects administered SRK-015 in the SRK-015-001 study was 81 (58 to 104) kg in the SAD, and 87 (52 to 107) kg in the MAD. Body weight at Baseline was determined to be a significant covariate on volume of distribution, but not on clearance (Bilic 2023).

Simulations performed using the population PK-PD model show that up to 1.85-fold, and up to 2.2-fold higher exposures of apitegromab may be expected in an obese subject with a body weight of 200 kg following a dose of 20 mg/kg, compared to exposures previously observed following 30 mg/kg Q2W in healthy adults, and exposures observed in SMA subjects following 20 mg/kg Q4W, respectively (Table 4).

Table 4: Simulated Exposure in Obese Subject (200 kg) and Comparison to Exposures in Prior Studies

Dose	Simulated Exposure		Exposure Increase in 200 kg Obese Subject (Fold Increase)	
	C_{maxss} (µg/mL)	AUC_{ss} (µg.day/mL)	C_{maxss}	AUC_{ss}
20 mg/kg Q4W (200 kg Obese)	1290	23500	0.82×[a]/1.70×[b]	1.85×[a]/2.2×[b]
10 mg/kg Q4W (200 kg Obese)	643	11800	0.41×[a]/0.85×[b]	0.93×[a]/1.1×[b]

Abbreviations: AUC, area under the concentration-time curve; C_{max}, maximum serum concentration
Note: C_{max} and AUC increase in 200 kg obese subject is expressed relative to exposures observed at:
a the highest dose tested in healthy adults (SRK-015-001; 30 mg/kg Q2W; 52-107 kg): C_{max} = 1582 µg/mL, and AUC = 12735 µg.day/mL
b the highest dose tested in SMA subjects (SRK-015-002; 20 mg/kg Q4W; 15-96 kg): C_{max} = 758 µg/mL, and AUC = 10800 µg.day/mL

In order to not exceed the exposures observed in all clinical studies of apitegromab, including the highest exposures tested in healthy adults administered the highest dose of 30 mg/kg Q2W, the dose of 10 mg/kg was selected for overweight and obese adults. 10 mg/kg is 1 of the 2 doses that is being evaluated in the pivotal Phase 3 study of apitegromab in SMA.

In addition, pharmacodynamic (PD) results suggest that apitegromab treatment leads to robust increases in latent myostatin concentrations in serum, which demonstrates successful target engagement in humans. Moreover, the levels of target engagement attain a plateau, suggesting that the target is saturated even with a single treatment of apitegromab at doses ≥3 mg/kg. The total latent myostatin was sustained for approximately 84 days following a single 20 mg/kg dose, suggesting that the target engagement is durable. Simulations using the final PK-PD model show that there is no meaningful difference in levels of total latent myostatin between 10 mg/kg and 20 mg/kg dose in overweight and obese subjects.

Considering a 2-fold increase in exposure (PK), but similar PD effects for 20 mg/kg relative to 10 mg/kg doses in the target patient population, the lower dose of 10 mg/kg was chosen for once every 4-week dosing regimen in Study SRK-015-006 to allow for a greater margin of safety in overweight and obese adults.

[redacted]

4.5.1.2. Incretin Mimetic Therapy

For the administration of incretin mimetic therapy, subjects will be dosed based on an escalation schedule that is intended to minimize gastrointestinal adverse reactions associated with either drug (WEGOVY® USPI; ZEPBOUND™ USPI). Subjects will receive either tirzepatide at a maintenance dose of 5, 10, or 15 mg QW (for adults) or semaglutide at a maintenance dose of 2.4 mg QW (recommended) or 1.7 mg QW. In a retrospective study to evaluate weight loss outcomes associated with treatment with subcutaneous semaglutide, subjects lost approximately

6.7 kg at 3 months and 12.3 kg at 6 months, equivalent to 5.9% of weight lost at 3 months and 10.9% of weight lost at 6 months (Ghusn 2022). This was despite only 44.0% (N = 77) receiving the highest current doses of subcutaneous semaglutide (1.7 and 2.4 mg), while 56.0% (N = 98) received lower doses (0.25, 0.5, and 1 mg). Although clinical efficacy may be observed at lower doses of incretin mimetic therapy, weight loss is expected to increase as more patients achieve the maximal doses. Investigators in this study are asked to consider treatment response and tolerability when selecting the maintenance dosage of incretin mimetic therapy, and to either reduce the dose or remain on the same dose to avoid exacerbation of the adverse effects.

4.5.2. Dose Adjustment Criteria

4.5.2.1. Apitegromab or Placebo

There will be no dose adjustment for apitegromab or placebo. Criteria for dose modifications are location in Section 6.5.

4.5.2.2. Incretin Mimetic Therapy

Subjects will initiate dosing at the lowest dose QW and will follow the dose escalation schedule for incretin mimetic therapy. All subjects should aim at reaching the maximum tolerated maintenance dose (5, 10, or 15 mg QW for tirzepatide; 2.4 or 1.7 mg QW for semaglutide).

Depending on the subject's tolerability, the incretin mimetic therapy dosing schedule may be modified as follows:

- If the subject is able to tolerate the dose according to the schedule, then dose escalation should be followed (WEGOVY USPI; ZEPBOUND USPI; Table 8).
- If the subject is able to tolerate the current dose, but it is determined by the Investigator that the subject has reached the maximum tolerated dose (MTD), then the subject should be held at the current dose for up to 4 weeks. The subject will be evaluated at the next site visit for dose determination.
- If the subject is unable to tolerate the current dose, the subject should notify the site. If the subject is in between site visits, then the subject may return for an Unscheduled Visit for further evaluation by Investigator. The Investigator may determine 1 of the following:
    - decrease the dose to the MTD, or
    - discontinue the incretin mimetic if the subject is not able to tolerate the decreased dose, or
    - discontinue the incretin mimetic if the Investigator and/or the subject determine the subject is unable to tolerate both the current dose and the dose decrease to MTD.

4.5.3. Missed Doses

4.5.3.1. Apitegromab or Placebo

Any subject who misses a dose of apitegromab or placebo (within the window of ±7 days of the planned dosing visit) will not make up for the missed dose and will receive the next dose as scheduled in the SoA (Table 1).

4.5.3.2. Incretin Mimetic Therapy

If a subject misses 1 dose of incretin mimetic therapy and the next scheduled dose is more than 3 days away (72 hours), then the subject should administer the incretin mimetic therapy dose as soon as possible. If a subject misses 1 dose and the next scheduled dose is less than 3 days away (72 hours), then the subject should not administer the dose and should resume dosing on the regularly scheduled day of the week. If 2 or more consecutive doses of incretin mimetic therapy are missed, resume dosing as scheduled or, if needed, reinitiate incretin mimetic therapy and follow the dose escalation schedule, which may reduce the occurrence of gastrointestinal symptoms associated with re-initiation of treatment.

5. SELECTION AND WITHDRAWAL OF SUBJECTS

The subject inclusion and exclusion criteria are included in Section 5.1 and Section 5.2, respectively.

5.1. Subject Inclusion Criteria

Subjects must meet ALL the following inclusion criteria to be eligible for participation on this study:

1. Able to comprehend the informed consent process and provide written informed consent prior to study enrollment and the conduct of any study-related assessments.
2. Male or female, age ≥18 and ≤65 years old at the time of informed consent.
3. Stable body weight (±5 kg) within 90 days of Screening.
4. At Screening, a BMI of:
   a. ≥30 kg/m^2 to ≤45 kg/m^2 or
   b. ≥27.0 kg/m^2 to <30 kg/m^2 with the presence of 1 or more weight-related comorbid condition(s). Note: See exclusion criteria for specific organ class disease parameters
5. History of at least 1 self-reported unsuccessful dietary effort to lose body weight.
6. Able to receive study drug infusions via peripheral IV infusion catheter and provide blood samples via venipuncture or peripheral IV blood sampling catheter throughout the clinical study.
7. Able to adhere to the requirements of the protocol, including travel to the study site, and completing all study procedures, and study visits.
8. Females of childbearing potential must have a negative pregnancy test at Screening and agree to use 1 or more highly effective method of contraception throughout the clinical study and for 20 weeks after the last dose of apitegromab or placebo.
9. Must agree to refrain from any reconstructive and/or cosmetic surgery that may affect body weight during the study such as mammoplasty or lipoplasty.
10. Must agree to refrain from any bariatric treatments, endoscopic or surgical procedures during the study such as gastric balloons, or sleeve gastroplasty.
11. Must agree to refrain from injection of neurotoxin into non-facial large muscle groups that may affect LBM during the study such as gastrocnemius or trapezius slimming.
12. Must be fully ambulatory without any chronic orthopedic diseases or reliance on crutches, walkers, or a wheelchair that could preclude normal activity.

5.2. Subject Exclusion Criteria

Subjects who have ANY of the following criteria are not eligible for study participation:

1. History of or active cardiovascular disease including clinically significant arrhythmias such as atrial flutter, atrial fibrillation, and prolonged QT interval (QTc >450 msec for males and >470 msec for females); New York Heart Association Class 1 to 4 heart

failure; mild, moderate or severe coronary artery disease (CAD) or if not previously diagnosed with CAD, history of angina or symptoms consistent with CAD or other CV disease; uncontrolled hyperlipidemia; previous myocardial infarction, stroke, coronary artery bypass graft surgery, or percutaneous coronary intervention; deep vein thrombosis/pulmonary embolism; valve disorders or defects; abdominal aortic aneurysm; or pulmonary hypertension.

2. History of American Heart Association Stage 1 or Stage 2 hypertension that is not well-controlled to a normotensive range, or history of hypertensive crisis defined by AHA as systolic blood pressure >180 mmHg and/or diastolic blood pressure >120 mmHg, or resistant HTN defined as blood pressure that remains above goal despite concurrent use of 3 anti-HTN agents of different classes taken at maximally tolerated doses.
3. History of or active ischemic, hemorrhagic, or anatomical neurovascular disease including trans ischemic attack, cerebrovascular accident, arterio-venous malformation, or brain aneurysm.
4. History of or active peripheral vascular disease such as deep vein thrombosis, pulmonary embolism, chronic venous insufficiency, claudication, or lymphedema.
5. History of active pulmonary diseases including chronic obstructive pulmonary disease, pulmonary fibrosis, moderate-severe sleep apnea, and moderate-severe asthma.
6. Active malignancy, other than local subcutaneous squamous cell and basal cell carcinomas.
7. History of immunosuppressive, chemotherapeutic, or radiation treatment within the last 12 months prior to Screening.
8. History of Type 1 diabetes (T1D) or active T2D. If there was a history of T2D and it resolved, must be resolved >12 months prior to Screening. Prediabetes managed with non-pharmacologic approaches (lifestyle (exercise and diet) is not an exclusion.
9. History of apitegromab treatment.
10. Use of anti-obesity medications, nutritional supplements, or over-the-counter products for weight loss within 3 months of Screening.
11. Use of anti-diabetic medications, nutritional supplements, or over-the-counter products for lowering blood sugar within 3 months of Screening.
12. History of bariatric surgery or use of gastric balloons, or other gastric volume reduction types of devices.
13. History of gastroparesis, gastric or peptic ulcer, active gastritis or esophagitis, or uncontrolled gastroesophageal reflux disease, or severe inflammatory bowel disease.
14. Treatment with other investigational drugs in a clinical study within 3 months or 5 half-lives, whichever is longer, before Screening.
15. Use of medications known to induce weight gain such as some anti-convulsant and psychotropic medications within 3 months of screening.

16. Any chronic active infection or recent treatment within 6 months of Screening (eg, HIV, hepatitis B or C, tuberculosis, Long COVID).
17. Uncontrolled thyroid disease. Subjects with thyroid disease that have been treated with medications and are euthyroid for at least 3 months are not excluded. Subjects with a personal or family history of any type of thyroid cancer, including medullary thyroid cancer or multiple endocrine neoplasia Type 2 are excluded.
18. History of severe endocrine disorders such as Cushing's disease, hypogonadism, and growth hormone deficiency.
19. History of autoimmune/inflammatory disorders that may cause muscle wasting such as myasthenia gravis, rheumatoid arthritis, lupus, or inflammatory myopathies such as polymyositis or dermatomyositis.
20. History of neuromuscular disorders that may cause muscle wasting such as muscular dystrophy, SMA, and amyotrophic lateral sclerosis.
21. History of neurologic diseases such as epilepsy, dementia, Parkinson's disease, Bell's Palsy.
22. History of acute or chronic pancreatitis or clinically significant abnormal lipase and/or amylase or taking medications that may cause serious damage to the pancreas such as Valproic acid or are high risk for pancreatitis due to symptomatic cholelithiasis, chronic cholecystitis or untreated hypertriglyceridemia (≥500 mg/dL).
23. History or presence of severe active acute or chronic liver disease (eg, cirrhosis), hepatic insufficiency defined as Child Pugh Class A or higher, nonalcoholic fatty liver disease (NAFLD), nonalcoholic steatohepatitis (NASH), general hepatic disease including serum alanine aminotransferase (ALT) or aspartate aminotransferase (AST) ≥3× ULN.
24. Uncontrolled psychiatric disease including major depressive disorder (history of suicidal behavior or a Patient Health Questionnaire-9 (PHQ-9) score ≥15 at Screening), bipolar, anxiety disorder, or eating disorders such as bulimia, night eating syndrome (NES), or binge eating disorder.
25. Use of therapies with potentially significant muscle effects (eg, androgens, insulin-like growth factor, growth hormone, systemic beta-agonist, neurotoxins, or muscle relaxants or muscle-enhancing supplements) in any form for 3 months prior to Screening.
26. Use of systemic corticosteroids within 60 days before Screening. Inhaled or topical steroids are allowed. Use of intra-articular corticosteroid injections are prohibited.
27. Severe coagulopathy prothrombin time (PT)/international normalized ratio >3 seconds over control or platelet count <100,000) or is presently taking medications that impede coagulation or platelet aggregation or has a history or active coagulopathy disorder.
28. History of alcoholism, or illicit drug use (drugs that are illegal and have not been prescribed).
29. Using inhaled vasoconstrictive tobacco or cannabis or synthetic products such as vape pens, pipes, cigars, and cigarettes.
30. Chronic kidney disease Stages 1 to 5.

31. Any acute or comorbid condition interfering with the well-being of the subject within 7 days before Screening, including active systemic infection, the need for acute treatment, or inpatient observation due to any reason.
32. Contraindications to either tirzepatide or semaglutide (ZEPBOUND™ or WEGOVY®; specifically, any history of medullary thyroid carcinoma, or in subjects with multiple endocrine neoplasia syndrome type 2 (MEN-2), gallbladder disease or pancreatitis or prior history of cytokine release syndrome or Types I to IV hypersensitivity reaction to a mAb or recombinant protein bearing an Fc domain (eg, a soluble receptor-Fc fusion protein), apitegromab, or excipients of apitegromab.
33. Donation or loss of ≥500 mL (1 pint) of blood within 8 weeks prior to Screening or longer if required by local regulation, or plasma donation of ≥600 mL within 14 days prior to Screening.
34. Pregnant (has a positive blood or urine pregnancy test), is suspected of being pregnant, is breastfeeding, intends to become pregnant or is of childbearing potential but refuses to use adequate contraception.
35. Any other medical condition or clinically significant laboratory result or ECG findings that, in the opinion of the Investigator, may compromise safety or compliance, would preclude the subject from successful completion of the study, or interfere with the interpretation of the results (Examples: pregnancy, decision to start an intense weight training program, an AE that compromises safety).

5.3. Lifestyle Considerations

All subjects will receive standard care counseling with regards to lifestyle recommendations such as diet, physical activity, and behavior modification.

5.4. Screen Failures

Screen failures are defined as subjects who consent to participate in the clinical study but are not eligible for participation according to the inclusion/exclusion eligibility criteria and are not subsequently randomized to receive either apitegromab 10 mg/kg + incretin mimetic therapy or placebo + incretin mimetic therapy.

A minimal set of screen failure information is required to ensure transparent reporting of screen failure subjects to meet the Consolidated Standards of Reporting Trials (CONSORT) publishing requirements and to respond to queries from regulatory authorities. Minimal information includes date of informed consent, demography, screen failure details, eligibility criteria, and any serious adverse event (SAE).

Subjects who do not meet the criteria for participation in this study (screen failure) may be rescreened after a suitable period of time (the exact length is dependent upon the reason for the screen failure) per the documented agreement of the Sponsor and the Investigator.

If a subject is rescreened beyond 14 days from the previous ICF signature date, the subject must reconsent to study participation. All rescreened subjects will be assigned a new subject identification number. A subject may only rescreen once.

5.5. Criteria for Temporarily Delaying Randomization/Administration of Study Drug

If a subject who is determined to be eligible based on Screening has an acute or comorbid condition that causes a delay (occurs >28 days from Screening) in their Baseline (Visit 1), the subject may be eligible to rescreen, at the discretion of the Investigator.

5.6. Subject Withdrawal Criteria

5.6.1. Discontinuation of Study Drug and/or Study

Subjects may discontinue study drug and/or study or withdraw consent at any time and for any reason. Any discontinuation of study drug or from the study must be fully documented in the electronic case report form (eCRF). The Investigator may withdraw a subject from the study at any time if this is considered to be in the subject's best interest. Unless subject safety precludes doing so, the Medical Monitor should be consulted prior to stopping treatment or withdrawing a subject.

Subjects who discontinue study drug either temporarily or permanently should continue with the scheduled visits and assessments to ensure continued counselling and data collection. At the time of permanent discontinuation of study drug, the reason for discontinuation of treatment will be documented. If possible, an Early Termination (ET) Visit should be conducted within 8 weeks of the last dose (ie, End of Study [EOS]/ET Visit in Table 1).

5.6.1.1. Suspension of Dosing for all Subjects

Dosing for all subjects in the study may be suspended at any time for an emergent safety concern by the Medical Monitor, and/or Sponsor until the event can be evaluated completely and an appropriate course of action can be taken.

Applicable regulatory authority and IRB approval(s) must also be obtained before dosing is restarted.

5.6.1.2. Discontinuation of Study Treatment and/or Study Participation

Study treatment may be temporarily discontinued for an emergent safety event by the Investigator or Sponsor until the event can be evaluated.

The subject may be permanently discontinued from study treatment, and the study, if any of the following applies:

1. The subject is considered by the Investigator and/or Sponsor to have substantial noncompliance with the protocol or included in the study despite having violation(s) of the inclusion and/or exclusion criteria.
2. The subject has a treatment-emergent intolerable AE or SAE considered related to study treatment or clinically significant findings for which the investigator believes that discontinuation of study treatment is the appropriate measure (Section 9.2.3).
3. The subject has specific laboratory abnormalities (ie, acute pancreatitis, hepatic events, or elevated creatine kinase levels), as defined in Table 5 and in Section 9.5.4.2.

4. The subject becomes pregnant (the subject will be permanently discontinued and will be followed as described in Section 9.1.7).
5. Subject withdraws consent from study participation.
6. The subject is simultaneously participating in another clinical trial of an approved or non-approved investigational medicinal product.
7. Sponsor or Investigator decision.

Table 5: Specific Laboratory Abnormalities[a]/Clinical Findings Leading to Subject Discontinuation From Study Treatment/Study Participation

Laboratory Abnormality/Clinical Finding	Recommended Action
Creatine Kinase	
Levels >5× but <10× ULN and subject is:	
• Asymptomatic	Follow safety monitoring guidance for creatine kinase elevations (Section 9.2.5, Table 13) until there is no longer medical concern, or until symptoms worsen and meet criteria for discontinuation
• Symptomatic[b] with alternative explanation	
• Symptomatic with no alternative explanation	Discontinue study treatment
Levels >10× ULN and subject is:	
• Asymptomatic or symptomatic[b]	Discontinue study treatment
Hepatic Events	
ALT or AST >8× ULN	Follow safety monitoring guidance for hepatic events (Section 9.2.5, Table)
ALT or AST >5× for >2 weeks	
ALT or AST >3× ULN and total bilirubin >2× ULN or INR >1.5	
ALT or AST >3× ULN with the appearance of symptoms[c]	
ALT or AST >3× ULN and bilirubin is >2× ULN	Discontinue study treatment. Follow the reporting procedures for AESIs (Section 9.2.5)
Acute Pancreatitis	
If a subject demonstrates 2 of the 3 following criteria:	
• Abdominal pain	Discontinue study treatment. Follow the reporting procedures for AESIs (Section 9.2.5)
• Serum amylase or lipase ≥3× ULN	
• Findings consistent with pancreatitis on a cross-sectional abdominal imaging (CT or MRI)	

Abbreviations: AESI, adverse event of special interest; ALT, alanine transaminase; AST, aspartate transaminase; CT, computed tomography; INR, international normalized ratio; MRI, magnetic resonance imaging; ULN, upper limit of normal

a Laboratory values that meet the above criteria, in addition to other laboratory values that are out of range and represent a safety concern within the context of the overall clinical evaluation, must be repeated to confirm that the critical laboratory test values are correct.

b Muscle symptoms may include muscle pain or weakness associated with malaise or fever, rash, and/or urine discoloration.

c Fatigue, nausea, vomiting, right upper quadrant pain or tenderness, fever, rash, and/or eosinophilia (>5%).

The Investigator should promptly (within 24 hours) notify the Medical Monitor if study treatment is discontinued for a subject. If the reason to discontinue study drug is the occurrence of an SAE, the Investigator must report the SAE following the procedure in Section 9.6. The Investigator should request an ad hoc meeting with the Medical Monitor for any single event or combination of events that in his/her professional opinion may jeopardize the safety of the subject or the reliability of the data.

If study treatment is permanently discontinued due to an AE or other safety concerns and the subject does not want to continue with their monthly visit schedule, the subject will complete the assessments for the End of Treatment (EOT) Visit (Table 1) and continue to be followed for 20 weeks after their final dose of apitegromab or placebo or until the resolution of any ongoing clinically significant AE, whichever occurs later.

Subjects who develop either an SAE or other findings meeting the individual temporary discontinuation criteria listed above will be carefully monitored and may be required to have additional assessments, including additional clinical laboratory tests and/or other clinical investigations as needed and at the discretion of the Investigator.

At the time of withdrawal/discontinuation from the study, if possible, an ET Visit should be conducted within 8 weeks of the last dose (ie, EOS/ET Visit in Table 1). Any SAE will be followed until resolution, stabilization, the event is otherwise explained, or the subject is lost to follow-up (Section 9.8).

If a subject withdraws consent, the Sponsor may retain and continue to use any data collected before such a withdrawal of consent.

If a subject withdraws from the study, the subject may request destruction of any samples taken and not tested, and the Investigator must document this in the site records.

5.6.2. Lost to Follow-up

A subject will be considered lost to follow-up if they fail to return for a scheduled visit, within the protocol-defined window, and are unable to be contacted by the clinical study site after multiple attempts to reach them.

The following actions must be taken if a subject fails to return to the clinic for a required clinical study visit:

- The site must attempt to contact the subject and reschedule the missed visit as soon as possible and counsel the subject on the importance of maintaining the assigned visit schedule and ascertain whether the subject wishes to and/or should continue in the clinical study.
- Before a subject is deemed lost to follow-up, the Investigator or designee must make every effort to regain contact with the subject (where possible, 3 telephone calls and, if necessary, a certified letter to the subject’s last known mailing address or local equivalent methods). These contact attempts should be documented in the subject’s medical record.

- Should the subject continue to be unreachable, they will be considered to have withdrawn from the clinical study with a primary reason of lost to follow-up.

6. TREATMENT OF SUBJECTS

6.1. Description of Study Drug

Study drug is defined as any investigational intervention(s), marketed product(s), or placebo intended to be administered to a subject according to the study protocol.

6.1.1. Apitegromab or Placebo

Characteristics of apitegromab or placebo are shown in Table 6.

Table 6: Investigational Product

	**Investigational Product**	
**Product Name**	**Apitegromab**	**Placebo**
**Type**	Biologic	Biologic
**Dosage Formulation**	IV infusion	IV infusion
**Unit Dose Strength**	50 mg/mL fully human mAb that specifically binds to precursor forms of myostatin	Not applicable
**Excipients**	[redacted]	[redacted]
**Route of Administration**	IV infusion	IV infusion
**Dosage Level**	10 mg/kg Q4W	Q4W
**Infusion Time**	[redacted]	[redacted]
**Use**	Experimental	Placebo
**Physical Description**	[redacted]	[redacted]
**Manufacturer**	Scholar Rock, Inc.	Scholar Rock, Inc.

Abbreviations: IV, intravenous; mAb, monoclonal antibody; Q4W, once every 4 weeks

6.1.2. Incretin Mimetic Therapy

6.1.2.1. Tirzepatide

Tirzepatide injection, for subcutaneous use, contains tirzepatide, a GIP receptor and GLP-1 receptor agonist. Tirzepatide is a clear, colorless to slightly yellow, sterile, preservative-free solution available in prefilled single-dose pens containing 0.5 mL solution of 2.5, 5, 7.5, 10, 12.5, or 15 mg of tirzepatide.

Subjects will receive tirzepatide in a dose escalation regimen, as shown in Table 7. Investigators will initiate dosing at the lowest dose QW and should follow the recommended dose escalation schedule. The recommended maintenance dose of tirzepatide therapy QW should be targeted for all subjects.

Table 7: Recommended Dose Escalation Schedule for Tirzepatide

Interval	Weeks	Weekly Dose	Volume for Injection
Dose Initiation	1 to 4	2.5 mg	0.5 mL
Dose Escalation	5 to 8	5.0 mg	0.5 mL
	9 to 12	7.5 mg	0.5 mL
	13 to 16	10.0 mg	0.5 mL
	17 to 20	12.5 mg	0.5 mL
	21 and onward	15 mg	0.5 mL
Dose Maintenance	Subject to treatment response and tolerability	5 mg, 10 mg or 15 mg	0.5 mL

6.1.2.2. Semaglutide

Semaglutide injection, for subcutaneous use, contains semaglutide, a GLP-1 analog with 94% sequence homology to human GLP-1.

Semaglutide is a clear, colorless solution available in a prefilled, disposable, injection pen with an integrated needle for subcutaneous injection in the abdomen, thigh, or upper arm. Semaglutide is supplied in cartons containing 4 pen-injectors—enough for 4 weekly injections. Each 0.5 mL single-dose pen contains a solution of semaglutide containing 0.25, 0.5, or 1 mg of semaglutide; each 0.75 mL single-dose pen contains a solution of semaglutide containing 1.7 or 2.4 mg of semaglutide.

Subjects will receive semaglutide in a dose escalation regimen, as shown in Table 8. Investigators will initiate dosing at the lowest dose QW and should follow the recommended dose escalation schedule. The recommended maintenance dose of semaglutide therapy QW should be targeted for all subjects.

Table 8: Recommended Dose Escalation Schedule for Semaglutide

Interval	Weeks	Weekly Dose	Volume for Injection
Dose Initiation	1 to 4	0.25 mg	0.5 mL
Dose Escalation	5 to 8	0.5 mg	0.5 mL
	9 to 12	1.0 mg	0.5 mL
	13 to 16	1.7 mg	0.75 mL
Dose Maintenance	17 and onward	2.4 mg or 1.7 mg	0.75 mL

6.1.2.3. Dose Modification

Depending on the subject's tolerability, the incretin mimetic dosing schedule may be modified as follows:

- If the subject is able to tolerate the dose according to the schedule, then dose escalation should be followed (Table 7 or Table 8).
- If the subject is able to tolerate the current dose, but it is determined by the Investigator that the subject has reached the MTD, then the subject should be held at the current dose for up to 4 weeks. The subject will be evaluated at the next site visit for dose determination.
- If the subject is unable to tolerate their current dose, the subject should notify the site. If the subject is in between site visits, then the subject may return for an Unscheduled Visit for further evaluation by the Investigator. The Investigator may determine 1 of the following:
    - decrease the dose to the MTD or;
    - discontinue the incretin mimetic if the subject is not able to tolerate the decreased dose or;
    - discontinue the incretin mimetic if the Investigator and/or the subject determines the subject is unable to tolerate both the current and decreased dose to MTD.

All subjects will receive the first administration of incretin mimetic therapy at the study site on Visit 1 (Day 1), at which time they will also be trained to self-administer incretin mimetic therapy QW, using the injection pen, and will receive a 1-month supply of incretin mimetic therapy. Thereafter, all subjects will receive additional 1-month supplies of incretin mimetic therapy at the Q4W visits (Visit 2 [Day 29], Visit 3 [Day 57], Visit 4 [Day 85], Visit 5 [Day 113], and Visit 6 [Day 141]). If the subject does not tolerate the dose, the subject may return for an Unscheduled Visit for dose determination. All subjects, regardless of the incretin mimetic dose they are receiving, must bring used and/or unused incretin mimetic pens to the site at each study visit for accountability and reconciliation.

6.2. Concomitant Medications

Concomitant therapies or interventional procedures that are medically indicated for any AEs the subject has during the study or that are provided as part of standard supportive care for the subject, are permitted at the discretion of the Investigator and supersede any of the restrictions outlined in this protocol.

Investigational therapies are not permitted throughout the duration of the study. The concomitant use of the following prescription medications, supplements, or over-the-counter drugs or product classes listed below are prohibited from Baseline through the final visit.

- Anti-obesity medications
- Anti-diabetic medications
- Medications that may cause serious risk to the pancreas, eg, valproic acid, diuretics
- Medications that have potentially significant muscle anabolic or catabolic effects
- Medications that are associated with weight gain

As this list is not exhaustive, please consult the Study Manual or contact the Medical Monitor if there are questions about concomitant medications.

6.3. Treatment Compliance

Apitegromab or placebo will be administered under the supervision of the Investigator or qualified study personnel. The date and start/end time of each infusion administered will be recorded in the source documents. The dose of study drug and study subject identification will be confirmed at the time of dosing. The study site is required to adhere to all applicable laws, regulations, and guidelines including, but not limited to, the US Code of Federal Regulations (CFR), the International Council for Harmonisation of Technical Requirements for Pharmaceuticals for Human Use (ICH), the Health Insurance Portability and Accountability Act (HIPAA) of 1996, as well as any applicable local and federal regulations.

Incretin mimetic therapy should be administered per the ZEPBOUND USPI or WEGOVY USPI.

6.4. Randomization and Blinding

Subjects will be randomized after the Investigator has verified that they are eligible to participate in the study per the criteria in Section 5.1 and Section 5.2. Subjects will be randomized 1:1 via IWRS in a double-blind manner to receive apitegromab + incretin mimetic therapy or placebo + incretin mimetic therapy.

The Sponsor, subjects, Investigators, and site personnel, with the exception of the Pharmacist, will be blinded to treatment assignments.

The site Pharmacist will remain unblinded throughout the duration of the trial. If for any reason Sponsor personnel need to be unblinded, the list of personnel and the reason for unblinding will be documented. In the event of a drug-related, serious, unexpected AE, designated unblinded Sponsor personnel may provide a subject's treatment assignment for the purpose of regulatory authority agency reporting. In the event of a drug-related SAE, the Investigator may, if deemed

medically necessary to provide subject care, obtain the subject's treatment assignment from the IWRS.

In case of an emergency, the Investigator will determine if unblinding of a subject's treatment assignment is warranted. If a subject's treatment assignment is to be unblinded, the Sponsor must be notified as soon as possible but no later than 24 hours after breaking the blind. The date and reason that the blind was broken must be recorded in the source documentation and electronic case report form (eCRF), as applicable.

6.5. Dose Modification

If an acute infusion reaction occurs, further dosing of apitegromab or placebo will be suspended and the Investigator, in consultation with the Medical Monitor if needed, will evaluate the risk represented by the acute reaction. Such an evaluation will incorporate consideration of the nature of the event, relatedness to the drug, and seriousness and severity of the event.

Intervention for subjects who have an acute infusion reaction should be performed in accordance with standard procedures and may include restarting the infusion at a slower rate, terminating the infusion, administration of medications, or other medically supportive measures, as necessary.

See Section 6.1.2.3 for dose modification recommendations for incretin mimetic therapy.

6.6. Treatment of Overdose

6.6.1. Apitegromab or Placebo

An overdose is defined as a significant variation from the recommended/scheduled dosage for a product. Administration of study drug for this clinical study will be performed in a controlled clinical setting and an overdose is not anticipated. However, in the event of an accident, any dose of study drug that is at least 2-fold higher than 10 mg/kg will be considered an overdose. Two doses within 1 week will also be considered an overdose from a treatment perspective.

Overdoses are not considered AEs and should not be recorded as an AE in the electronic case report form (eCRF) unless an AE or an SAE occurs. All overdoses (regardless of whether or not they result in an AE) must be recorded on an overdose form. If an overdose results in an SAE, both the SAE and overdose forms must be completed.

In the event of an overdose, the Investigator or designee:

- Must contact the Medical Monitor within 24 hours.
- Should evaluate the subject to determine, in consultation with the Medical Monitor, whether study drug should be interrupted or whether the dose should be reduced. The evaluation process should be complete prior to the next infusion.
- Should closely monitor the subject for any AE/SAE and laboratory abnormalities for the remainder of the clinical study.
- In case of an AE or SAE, the Investigator, in conjunction with the Medical Monitor, may choose to stop the drug until the AE reduces in severity to Grade 1 (mild) or resolves completely. The decision to restart the study drug in this case should also be made in consultation with the Sponsor.

- Should document the quantity of the excess dose as well as the duration of the overdose.
- Must submit the completed Overdose Form to Safety within 24 hours of the site becoming aware of the overdose. Contacts for reporting the overdose can be found on the Overdose Form in the site binder.
- If applicable (ie, the overdose results in an SAE; Section 9.2.3), the Investigator must submit the SAE to Safety within 24 hours of the site becoming aware of the SAE. Refer to Section 9.6 for requirements for reporting SAEs.

6.6.2. Incretin Mimetic Therapy

For an overdose of tirzepatide or semaglutide, refer to the current prescribing information specific for each incretin mimetic therapy (WEGOVY USPI, ZEPBOUND USPI).

6.7. Unscheduled Visit

It is at the Investigator's discretion to initiate an Unscheduled Visit, if deemed necessary for the incretin mimetic dosing schedule or for the subject's safety and well-being. An Unscheduled Visit may occur due to the following, but not limited to, reasons:

- Safety reasons
- Incretin mimetic dosing determination
- Pregnancy test

All such visits will be documented in the electronic case report form (eCRF), with any additional required documentation based on the nature of the Unscheduled Visit. The date and reason for the Unscheduled Visit must be recorded in the source documentation.

Procedures that can be performed at an Unscheduled Visit are defined in the SoA (Table 1). The Investigator can decide which of the remaining assessments (any and/or all) need to be performed at each Unscheduled Visit. The Investigator should consult with the Medical Monitor, if needed, to discuss Unscheduled Visit testing.

6.8. Continued Access to Study Drug After the End of Treatment

Not applicable.

6.9. Criteria for Study Termination

6.9.1. End of Study Definition

The End of Study is defined as the timepoint when the final data point is collected from the last subject in the study.

A subject is considered to have completed the study if he/she has completed the Screening, Treatment, and Safety Follow-up Periods or Early Termination visit. This may also be when the subject withdraws consent, is discontinued, or is lost to follow-up.

Alternatively, End of Study is when the Sponsor may decide to terminate the study. The Sponsor shall promptly inform the Investigators, the regulatory authorities, and any CRO(s) used in the

trial of the reason for termination or suspension, as specified by the applicable regulatory requirements. Should this occur, prematurely discontinued subjects should be seen as soon as possible for an EOT or EOS Visit, as applicable.

The Investigator is responsible for informing Institutional Review Boards (IRBs)/Independent Ethics Committees (IECs) of the Early Termination of the study. The Investigator should promptly inform the subject and assure appropriate subject therapy and/or follow-up.

6.9.2. Study Termination

If the study is prematurely terminated or suspended, the Sponsor shall promptly inform the Investigators, the IECs/IRBs, the regulatory authorities, and any contract research organization(s) used in the study of the reason for termination or suspension, as specified by the applicable regulatory requirements. The Investigator shall promptly inform the subject and should assure appropriate subject therapy and/or follow-up. Subjects will continue to be monitored for adverse events for 20 weeks after their final dose of apitegromab or placebo.

Criteria for study termination include the assessment of safety concerns that may arise during the conduct of the study or from data from the apitegromab nonclinical and clinical program. The study may be terminated if the Sponsor determines that further drug exposure would pose an undue risk to subjects.

For study termination:

- Discontinuation of further study drug development
- Safety data suggesting that further drug exposure would pose an undue risk to subjects

6.9.3. Site Closure

The Sponsor or designee reserves the right to close the study site at any time for any reason at the sole discretion of the Sponsor.

Study sites will be closed upon study completion. A study site is considered closed when all required documents and study supplies have been collected and a study site closure visit has been performed.

The Investigator may initiate study site closure at any time, provided there is reasonable cause and sufficient notice is given in advance of the intended termination.

Reasons for the early closure of a study site by the Sponsor or Investigator may include but are not limited to:

For site termination:

- Failure of the Investigator to comply with the protocol, the requirements of the IRB/IEC or local health authorities, the Sponsor's procedures, or GCP guidelines
- Inadequate or no recruitment (evaluated after a reasonable amount of time) of subjects by the Investigator

7. STUDY DRUG MATERIALS AND MANAGEMENT

7.1. Study Drug

Apitegromab is an IgG4/lambda isotype, [redacted]

For a description of tirzepatide, refer to the prescribing information (ZEPBOUND PI). For a description of semaglutide, refer to the prescribing information (WEGOVY PI).

7.2. Study Drug Packaging and Labeling

The apitegromab drug product is supplied in a Type I borosilicate glass vial with a Flurotec®-coated, bromobutyl or chlorobutyl rubber stopper and crimp seal with a flip-off cap for single-use administration.

For a description of the tirzepatide packaging and labeling information, refer to the prescribing information (ZEPBOUND PI). For a description of the semaglutide packaging and labeling information, refer to the prescribing information (WEGOVY PI).

7.3. Study Drug Preparation/Handling/Storage/Accountability

7.3.1. Apitegromab or Placebo

The Investigators and site personnel, with the exception of the Pharmacist, will be blinded to treatment assignments. The site Pharmacist will remain unblinded throughout the duration of the trial.

Instructions for preparation of each IV dose of apitegromab or placebo will be provided to the Pharmacist. Preparation and dispensing of the study drug will be handled by the site pharmacy. Instructions for safe handling of the study drug are provided in the Pharmacy Manual:

1. The Investigator or designee must confirm appropriate temperature conditions have been maintained during transit for all study drug received and any discrepancies are reported and resolved before use of the study drug.
2. Only subjects randomized in the study may receive study drug and only authorized site staff may supply or administer study drug.
3. All study drug must be stored in a secure, environmentally controlled, and monitored (manual or automated) area in accordance with the labeled storage conditions [redacted]
4. The Investigator (or designee) is responsible for maintaining accurate accountability records of the investigational product throughout the clinical study. The site Pharmacist will inventory the investigational product received and will maintain records of disposition of the drug, including dates, quantity, and use. All dispensing and accountability records will be available for Sponsor review. Study drug accountability

will be verified during on-site monitoring visits. At the end of the study, Study Monitors or designees will conduct a final accountability of all study drugs.

5. Following accountability of study drug by a Study Monitor, used vials may be destroyed at the site according to local standard operating procedures containing well documented destruction procedures.
6. Unused vials should be returned to the Sponsor, or its designated storage location, for final disposition.

7.3.2. Incretin Mimetic Therapy

For a description of the recommended storage and handling information for tirzepatide, refer to the prescribing information (ZEPBOUND PI). For a description of the recommended storage and handling information for semaglutide, refer to the prescribing information (WEGOVY US PI).

7.4. Study Drug Administration

7.4.1. Apitegromab or Placebo

Apitegromab [redacted]

For administration, apitegromab or placebo is diluted in normal saline as a solution for infusion.

The apitegromab or placebo dose regimen will be 10 mg/kg Q4W. The dose will be administered by IV infusion. For the first 2 doses (Visits 1 and 2), the infusion will be administered over [redacted]

7.4.2. Incretin Mimetic Therapy

Subjects may receive either tirzepatide or semaglutide, but will receive only one throughout the treatment period, per IWRS assignment.

7.4.2.1. Tirzepatide

Tirzepatide is a clear, colorless to slightly yellow, sterile, preservative-free solution for subcutaneous use. Each single-dose pen contains 0.5 mL solution of 2.5 mg, 5 mg, 7.5 mg, 10 mg, 12.5 mg, or 15 mg of tirzepatide and the following excipients: sodium chloride (4.1 mg), sodium phosphate dibasic heptahydrate (0.7 mg), and water for injection. Hydrochloric acid solution and/or sodium hydroxide solution may have been added to adjust the pH. Tirzepatide has a pH of 6.5 to 7.5. For important monitoring and administration instructions, refer to the prescribing information for tirzepatide (ZEPBOUND PI).

7.4.2.2. Semaglutide

Semaglutide is a clear, colorless solution available in a prefilled, disposable, single-dose injection pen with an integrated needle for subcutaneous injection in the abdomen, thigh, or upper arm. Semaglutide is supplied in cartons containing 4 pen-injectors—enough for 4 weekly injections. Each 0.5 mL single-dose pen contains a solution of semaglutide containing 0.25 mg, 0.5 mg, or 1 mg of semaglutide; each 0.75 mL single-dose pen contains a solution of semaglutide containing 1.7 or 2.4 mg of semaglutide. For important monitoring and administration instructions, refer to the prescribing information for semaglutide (WEGOVY US PI).

8. ASSESSMENT OF EFFICACY

8.1. Anthropometric Measurements

Training to standardize performance of anthropometric measurements across study sites will be performed. Please refer to the Study Manual for additional information.

8.1.1. Height

Standing Screening height will be measured in bare feet.

8.1.2. Weight

Subjects shall be weighed with only their undergarments or a clinic/hospital gown on. Shoes and socks shall be removed. The scale shall be calibrated per the procedures of the study site.

8.1.3. Waist and Hip Circumference Measurements

A non-stretchable tape measure shall be used for measuring. Measurements shall be taken on bare skin (not on top of clothing). The tape should be snug but not compressing the skin. Read the measurements at the end of normal expiration. The measurements shall be taken while the subject is standing with their feet together.

Waist circumference measurements: first locate the top of the iliac crest (hip bone). Using the uppermost lateral top border of the iliac crest, bring the tape measure around the abdomen in a horizontal plane. Ensure the tape is flat against the skin (CDC 2023).

Hip circumference measurements: first locate the widest portion of the gluteal region (usually between the greater trochanter [top of the thigh bone] and the lower gluteal region). Bring the tape measure around the widest portion in a horizontal plane. Ensure the tape is flat against the skin (Czernichow 2011, NHS 2023).

8.1.4. Body Mass Index Calculation

The screening height and weight data shall be used in the calculation of the screening BMI. The BMI will be calculated by using the standard BMI calculator recommended by the National Institutes of Health (NIH) that is available on their website using weight and height measurements described above. The results will be classified based on BMI categories: underweight, normal weight, overweight, or obesity (NIH 2023). If the BMI meets the obesity classification, it will be further subclassified into Class 1, Class 2, and Class 3 obesity (CDC 2023).

8.2. Handheld Dynamometry Assessment

Handheld dynamometry (HHD) will be used to assess physical function in subjects at visits indicated in the SoA (Table 1). Qualified site staff will oversee the HHD.

8.3. Chair Sit-to-Stand Test

A chair sit-to-stand test will be used to assess physical function and endurance in subjects at visits indicated in the SoA (Table 1). Qualified site staff will oversee the test.

8.4. Efficacy Laboratory Tests

Changes in cardiometabolic parameters (eg, glucose, lipid metabolism, exploratory biomarker) will be evaluated by laboratory measurements, as listed in Table 9. Subjects must fast for 10 to 12 hours prior to all blood draws.

Table 9: Efficacy Laboratory Assessments

Glucose Homeostasis Tests	Lipid Panel Tests	[redacted]
• Plasma glucose[a]<br>• Serum insulin<br>• HbA1c (glycated hemoglobin)<br>• C-peptide	• Total cholesterol<br>• Triglycerides<br>• High-density lipoprotein<br>• Low-density lipoprotein<br>• Very low-density lipoprotein	• [redacted]

[a] Note that plasma glucose is also part of the safety laboratory tests and does not need to be drawn twice. It should be drawn as part of the safety laboratory tests. The test value, in addition to safety assessment, will also be used in the exploratory efficacy assessment of glucose homeostasis tests.

The Sponsor shall calculate the homeostatic model assessment of insulin resistance (HOMA-IR) using the fasting glucose and insulin measurements.

8.5. Blood Pressure

Changes in systolic and diastolic blood pressure will be evaluated. Note that blood pressure is also part of the vital sign assessments in the assessment of safety (Section 9.1.2).

Blood pressure using a completely automated device shall be taken at rest in a sitting position after the subject has rested for at least 5 minutes. Ensure the cuff is on the lower part of the upper arm at chest height and is the appropriate size for the subject. The cuff shall be on bare skin and not over clothing. Instruct the subject not to speak during the actual measurement.

9. ASSESSMENT OF SAFETY

9.1. Safety Parameters

9.1.1. Demographics/Medical History/Weight Management History

Subject demographics and medical history will be recorded on the source document and eCRF. Demographic characteristics include age, sex, race, and ethnicity. Medical history will capture the subject's past and current medical conditions including weight-related comorbid conditions (eg, hypertension or dyslipidemia), and non-weight-related comorbid conditions, past surgical history, drug and environmental allergies, and concomitant medications.

Weight management history will include prior attempts at weight loss including interventions tried such as diets, exercise, medications, weight loss programs, behavioral therapy.

9.1.2. Vital Signs

Vital signs will be performed at rest by the Investigator or his/her qualified designee. Vital sign assessments will include heart rate, blood pressure, body temperature, and respiratory rate. Baseline vital signs will be collected pre- and post-infusion. Subjects will remain at the study site for 1 hour for AE monitoring after each EOI but may be observed longer at the Investigator's discretion. Subjects will have vital signs checked again before leaving the study site.

Heart rate shall be recorded as part of the automated blood pressure and heart rate device. Temperature and respiratory rate will follow the standard clinical practice at the site.

9.1.3. Physical Examination

A physical examination will be performed by the Investigator or a qualified designee. The components of the physical examination will include an assessment of the following: general appearance, skin, lymph nodes, head-eyes-ears-nose-throat, neck, abdomen, respiratory, cardiovascular, musculoskeletal, and neurologic body systems.

Clinically significant findings at the Screening visit will be considered part of Medical History. Clinically significant physical examination findings post-Screening are considered AEs.

9.1.4. Electrocardiogram

Triplicate 12-lead ECGs are to be performed with the subject in the supine position after having rested for at least 5 minutes before the first reading and remain resting during the subsequent readings. The 3 individual ECG tracings should be obtained as closely as possible in succession, but no more than 2 minutes apart. The ECGs will be obtained using an ECG machine that automatically calculates the heart rate and measures PR, QRS, QT, and QTcF intervals.

9.1.5. Safety Laboratory Tests

Laboratory testing will be performed using established methods and at study visits indicated in the SoA (Table 1). An overview of the safety laboratory tests is shown in Table 10.

Table 10: Safety Laboratory Assessments

Hematology	Coagulation	Electrolytes	Liver
WBC count with differential<br>RBC<br>Hb<br>Hematocrit<br>MCHC<br>MCV<br>MCH<br>Platelet count	aPTT<br>PT<br>INR	Na<br>K<br>Cl<br>HCO_3<br>Mg<br>PO_4<br>Ca	Albumin<br>ALT<br>AST<br>Alkaline phosphatase<br>Bilirubin (direct, indirect, total)<br>Total protein<br>GGT
**Muscle**	**Thyroid**	**Pancreas**	**Kidney**
Creatine kinase<br>LDH<br>LDH isoenzyme	TSH	Amylase<br>Lipase	BUN<br>Creatinine<br>Glucose<br>Uric acid<br>eGFR

Abbreviations: ALT, alanine transaminase; AST, aspartate aminotransferase; aPPT, activated partial thromboplastin time; BUN, blood urea nitrogen; Ca, calcium; Cl, chloride; eGFR, estimated glomerular filtration rate; GGT, gamma-glutamyl transferase; HCO_3, bicarbonate; Hb, hemoglobin; INR, international normalized ratio; K, potassium; LDH, lactate dehydrogenase; MCH, mean corpuscular hemoglobin; MCHC, mean corpuscular hemoglobin concentration; MCV, mean corpuscular volume; Mg, magnesium; Na, sodium; PO_4, phosphate; PT, prothrombin time; RBC, red blood cell; TSH, thyroid stimulating hormone; WBC, white blood cell

Table 11: Urinalysis Assessments

Laboratory Assessments[a]	
Appearance	Leukocyte esterase
Bilirubin	Nitrite
Blood	pH
Color	Protein
Glucose	Specific gravity
Ketone	Urobilinogen

[a] A urine microscopic panel will be run if urinalysis is abnormal.

See Section 9.2.5 for details on monitoring for increases in creatine kinase.

9.1.5.1. [redacted]

[redacted]

[redacted]

9.1.6. Site Check-In Telephone Call

The purpose of the site staff check-in telephone call to the subject during the Treatment and during the Safety Follow-up Period is to monitor AEs and concomitant medication information. Subjects will also be asked if they have any questions regarding the self-administration procedure for incretin mimetic therapy.

The site staff will elicit AE information and record any new or change in ongoing signs and/or symptoms as well as any events that have been resolved since the last evaluation. The site staff will review the current list of concomitant medications and note any medications that were started or stopped.

During the Treatment Period, the site will contact subjects by telephone within 7 days after each dosing visit to collect information on AEs and concomitant medications.

During the Safety Follow-up Period, the site will contact subjects by telephone within 28 days after visit to check for AEs and concomitant medications.

9.1.7. Pregnancy

A pregnancy test (females of childbearing potential only) will be assessed at intervals per the SoA (Table 1):

- Details of all pregnancies in female subjects will be collected after the start of study drug and until 20 weeks after the last dose of apitegromab (the study drug with the longest half-life) or placebo.
- If a pregnancy is reported, the Investigator will record pregnancy information on the appropriate form and submit it to Safety within 24 hours of learning of the female subject pregnancy.
- While pregnancy itself is not considered to be an AE or SAE, any pregnancy complication or elective termination of a pregnancy for medical reasons will be reported as an AE or SAE.
- Abnormal pregnancy outcomes (eg, spontaneous abortion, fetal death, stillbirth, congenital anomalies, ectopic pregnancy) are considered SAEs and will be reported as such.
- The subject will be followed to determine the outcome of the pregnancy. The Investigator should collect follow-up information on the subject and the neonate, and the information will be forwarded to Safety.
- Any post-clinical study pregnancy-related SAE considered reasonably related to the study drug by the Investigator will be reported to Safety. Although the Investigator is not obligated to actively seek this information in former study subjects, he or she may learn of an SAE through spontaneous reporting.

- Any female subject who becomes pregnant while participating in the clinical study will discontinue study drug and be discontinued from the clinical study.

9.1.8. Psychiatric Evaluations

The Columbia Suicide Severity Rating Scale (C-SSRS) and PHQ-9 will be utilized to assess baseline and treatment-emergent depression and suicidal ideation and behavior in subjects at visits per the SoA (Table 1).

9.1.8.1. Columbia Suicide Severity Rating Scale

The C-SSRS is a validated instrument to prospectively assess suicidal ideation and behavior. The C-SSRS questionnaire will be administered as an interview by the Investigator or a qualified study center personnel. There are specific questionnaires designed for use at screening and at baseline (Baseline/Screening version) and visits throughout the study (Since Last Visit version). All versions of the C-SSRS include a series of screening questions related to suicidal ideation and suicidal behavior (Posner 2011). Subject responses of "yes" to one or more screening questions will prompt additional questions that evaluate the frequency and intensity of suicidal ideation and/or behavior.

The C-SSRS will be assessed at the timepoints indicated in Table 1. The "Baseline/Screening" version of the C-SSRS will be used at screening. The "Since Last Visit" version of the C-SSRS will be used at all other study visits.

9.1.8.2. Patient Health Questionnaire - 9

The PHQ-9 is a validated self-administered screening tool that assesses the presence and intensity of depressive symptoms. The PHQ-9, which incorporates the 9 Diagnostic and Statistical Manual IV depression criteria as "0" (not at all) to "3" (nearly every day), was developed for use in primary care settings (Kroenke 2001).

During the study conduct, a subject will be referred to a mental health professional (MHP) for a PHQ-9 score of >10, any suicidal behavior, or any suicidal ideation of type 4 or 5 on the C-SSRS. A referral to an MHP should also be made if, in the opinion of the Investigator, it is necessary for the safety of the subject.

9.2. Adverse Events and Serious Adverse Events

9.2.1. Definition of Adverse Events

An AE is any untoward medical occurrence in a subject temporally associated with the use of study drug, whether or not it is considered related to the study drug. Thus, an AE can be any unfavorable and unintended sign (including laboratory finding), symptom, or disease (new or exacerbated) temporally associated with the use of a study drug. An AE can, therefore, be:

- Any abnormal laboratory test results (hematology, serum chemistry, or urinalysis) or other safety assessments (eg, ECG, radiological scans, vital signs measurements), including those that worsen from Baseline, considered clinically significant in the medical and scientific judgment of the Investigator. Specifically, an abnormal laboratory test result must be reported as an AE if it: a) is accompanied by clinical symptoms, b)

results in a change in study drug, or c) is clinically significant in the Investigator's judgment.

  - Note: if the laboratory abnormality can be characterized by a precise clinical term per standard definitions, the clinical term should be recorded as the AE. If a clinically significant laboratory abnormality is a sign of a disease or syndrome, only the diagnosis should be reported. Observations of the same clinically significant laboratory abnormality from visit to visit should only be recorded once as an AE.

- Exacerbation of a chronic or intermittent pre-existing condition including either an increase in frequency and/or intensity of the condition.
- New conditions detected or diagnosed after study drug administration even though it may have been present before the start of the trial.
- Signs, symptoms, or the clinical sequelae of a suspected drug-drug interaction.
- Signs, symptoms, or the clinical sequelae of a suspected overdose of either study drug or a concomitant medication. Overdose per se will not be reported as an AE/SAE unless it is an intentional overdose taken with possible suicidal/self-harming intent. Such overdoses should be reported regardless of sequelae.

"Lack of efficacy" or "failure of expected pharmacological action" will not be reported as an AE or SAE. Such instances will be captured in the efficacy assessments. However, the signs, symptoms, and/or clinical sequelae resulting from lack of efficacy will be reported as an AE or SAE if they fulfill the definition of an AE or SAE.

9.2.1.1. Special Cases

The following are special cases of AEs.

9.2.1.1.1. Adverse Events Secondary to Other Adverse Events

These AEs are also called "cascade events" or "clinical sequelae." In general, these AEs should be identified by their primary cause, except if they are severe or serious secondary AEs. If a medically significant secondary AE occurs, the AE may be recorded as an independent AE after consultation with the Sponsor. All AEs should be recorded separately if it is not evident whether the events are associated.

9.2.1.1.2. Persistent and Recurrent Adverse Events

A persistent AE is one that extends continuously, without resolution, between subject evaluation time points. These AEs should only be recorded once. The initial severity of the AE will be recorded at the time the event is first reported. If it becomes more severe, the most extreme severity should be recorded. If it becomes serious, the end date of the AE will be the date the AE became serious, which will be the same as the start date of the SAE. For this SAE, all requirements for SAEs apply.

A recurrent AE is one that resolves between subject visits and subsequently recurs. Each recurrence must be recorded as a separate AE.

9.2.1.2. Events NOT Meeting the Definition of an Adverse Event

The following events should not be recorded as an AE:

- Medical or surgical procedure (eg, endoscopy, appendectomy): the condition that leads to the procedure is the AE.
- Situations in which an untoward medical occurrence did not occur (social and/or convenience admission to a hospital).
- Anticipated day-to-day fluctuations of pre-existing disease(s) or condition(s) present or detected at the start of the trial that do not worsen.
- Findings discovered during Screening, or between Screening and the first dose, where it is reasonable to assume these findings were present before informed consent.

9.2.2. Procedure-related Adverse Events

Procedure-related AEs are those events considered by the Investigator to be related to the conduct of the clinical study, independent of the study drug. That is, the event may be related to the fact that a subject is participating in the study. Examples of procedure-related AEs include:

- Reactions at the injection site (eg, rashes, nodules, pain, bleeding)
- Pain, bruising, dizziness, syncope due to blood collection
- Shortness of breath, nausea, fall, bruises during exertion examinations
- Vomiting or dizziness due to fasting for an examination or injection
- Skin irritation, redness, or itching during an ECG

If the Investigator determines that the AE is associated with the study procedures, the Investigator must record this causal relationship in the source documents and eCRF, as appropriate, and report such an assessment in accordance with the SAE reporting requirements.

9.2.3. Definition of Serious Adverse Events

An SAE is an AE occurring during any study period (ie, Baseline, treatment, washout, or follow-up), and at any dose of the study drug that fulfills one or more of the following:

- Results in death
- Is immediately life-threatening
  - The term “life-threatening” in the definition of “serious” refers to an event in which the subject was at risk of death at the time of the event. It does not refer to an event which hypothetically might have caused death if it were more severe.
- Requires inpatient hospitalization or prolongation of existing hospitalization
  - In general, hospitalization signifies that the subject has been admitted (usually involving at least an overnight stay) at the hospital or emergency ward for observation and/or treatment that would not have been appropriate in the physician’s office or outpatient setting. Complications that occur during hospitalization are AEs. If a complication prolongs hospitalization or fulfills any other serious criteria, the

event is serious. When in doubt as to whether "hospitalization" occurred or was necessary, the AE should be considered serious.

- Emergency room visits/hospital admissions for AEs less than 24 hours in duration do not meet SAE criterion unless they meet any of the other SAE criteria in this list.
- A scheduled or elective hospitalization for medical/surgical procedure planned prior to ICF for a pre-existing condition that **has not worsened** from Baseline during participation in the study is not considered to be an SAE. However unexpected complications and/or prolongation of hospitalization that occur during elective surgery should be recorded as AEs and assessed for seriousness. Admission to the hospital for social or situational reasons (ie, no place to stay, too far away to come for hospital visits, respite care) will not be considered inpatient hospitalizations.

- Results in persistent or significant disability or incapacity
  - "Disability" means a substantial disruption of a person's ability to conduct normal life functions.
  - This definition is not intended to include experiences of relatively minor medical significance such as uncomplicated headache, nausea, vomiting, diarrhea, influenza, and accidental trauma (eg, sprained ankle) which may interfere with or prevent everyday life functions but do not constitute a substantial disruption.
- Is a congenital abnormality or birth defect
- Is an important medical event (eg, invasive or malignant cancers, intensive treatment for allergic bronchospasms) that may jeopardize the subject or may require medical intervention to prevent one of the outcomes listed above.

9.2.4. Treatment-Emergent Adverse Event

An AE is treatment-emergent if the onset time is after administration of the first dose of study drug through the final follow-up visit or, in the event that onset time precedes study drug administration, the AE increases in severity during the Safety Follow-up Period.

9.2.5. Adverse Events of Special Interest

This section includes AEs of special interest (AESI), whether due to observed safety findings, potential safety findings based on drug class, and/or agreed upon consultation with regulatory agencies. The Investigator must report any AESI to the Sponsor Medical Monitor or designee within 24 hours of becoming aware of the event regardless of whether or not it is serious or nonserious and regardless of causality. The AESIs will be reported according to applicable regulatory requirements. Summaries and analyses for the incidence of AESIs will be provided by treatment group. The details of the analysis of AESI will be provided in the statistical analysis plan (SAP). The following types of AEs are of special interest:

Pancreatitis

Acute pancreatitis has been observed with the use of GLP-1 RA drugs. A diagnosis of acute pancreatitis requires 2 out of the following 3 criteria:

- abdominal pain consistent with pancreatitis (often described as "a belt-like tightening")

- a serum amylase or lipase ≥3× ULN
- findings consistent with pancreatitis on cross-sectional abdominal imaging (computed tomography [CT] or magnetic resonance imaging [MRI])

If a subject is suspected of having acute pancreatitis, the following steps, and other procedures/assessments as deemed necessary by the Investigator, may be performed to confirm the diagnosis:

- conduct appropriate laboratory tests to measure levels of enzymes (eg, pancreatic amylase and lipase) associated with pancreatitis
- perform relevant imaging assessments, including abdominal CT scan with or without contrast, MRI, gallbladder ultrasound

Subjects with a confirmed diagnosis of acute pancreatitis should be discontinued from study treatment.

Liver Injury

The criteria for diagnosis of acute liver injury shall follow the Drug-Induced Liver Injury (DILI) guidelines and are based on the presence of hepatocellular injury, as indicated by increases in serum aminotransferase (AT) activities in the absence of other causes, and accompanied by increased serum total bilirubin levels without initial findings of cholestasis. Specifically, a diagnosis of DILI can be considered under the following conditions:

- **Elevated serum ATs**: The drug causes hepatocellular injury, generally shown by a higher incidence of ≥3× ULN of AST or ALT compared with the nonhepatotoxic control drug or placebo
- **Increased serum total bilirubin**: Among subjects showing such AT elevations, often with ATs much greater than 3× ULN, 1 or more also show elevation of serum total bilirubin >2× ULN without initial findings of choleostasis (elevated serum ALP)
- **Exclusion of other causes**: No other reason can be found to explain the combination of increased AT or total bilirubin. These reasons may be viral hepatitis A, B, or C; a pre-existing or acute liver disease; or another drug capable of causing the observed injury.

These criteria align with the observations of Hy's Law, which is considered a specific and strong predictor of a drug's potential to cause severe liver injury, including the need for a liver transplantation or resulting in death. Hy's Law cases are characterized by significant hepatocellular injury (as indicated by marked AT elevation) accompanied by jaundice (bilirubin >2× ULN) in the absence of cholestasis and other liver disease causes.

The steps for the clinical evaluation of DILI are shown in Table 12 .

Table 12: Clinical Evaluation of Drug-Induced Liver Injury

Event	Follow-Up Monitoring
**Detection of DILI**	• Obtain liver enzyme (ALT, AST, and ALP) and bilirubin tests every 2 to 4 weeks, at least for a few months during early drug trials. This helps in early detection of liver injury

Event	Follow-Up Monitoring
**Confirmation**	• Repeat testing within 48 to 72 hours for ALT, AST, ALP, and bilirubin to confirm abnormalities and determine if they are increasing or decreasing
**Close Observation**	• Initiate close observation immediately upon detection and confirmation of early signals of possible DILI. Close observation includes the following:<br>○ Repeat liver enzyme and serum bilirubin tests twice weekly or thrice weekly. The frequency of retesting can decrease to QW or less if abnormalities stabilize or the study drug has been discontinued and the subject is asymptomatic.<br>○ Obtain a more detailed history of symptoms and prior or concurrent diseases.<br>○ Obtain a history of concomitant drug use (including nonprescription medications and herbal and dietary supplement preparations), alcohol use, recreational drug use, and special diets.<br>○ Rule out acute viral hepatitis types A, B, C, D, and E; autoimmune or alcoholic hepatitis; NASH; hypoxic/ischemic hepatopathy; and biliary tract disease.<br>○ Obtain a history of exposure to environmental chemical agents.<br>○ Obtain additional tests to evaluate liver function, as appropriate (eg, INR and direct bilirubin).<br>○ Consider gastroenterology or hepatology consultations.
**Decision to stop study drug administration**	• The decision to stop drug administration due to potential DILI involves careful consideration of laboratory values and symptoms. The key points include the following:<br>○ Laboratory values: Persistent or significantly elevated liver enzymes (ALT or AST) indicate potential liver damage. Immediate action may not always lead to rapid improvement, as lab values and symptoms can continue to worsen even after stopping the drug.<br>○ Discontinuation of study drug should be considered if:<br>▪ ALT or AST >8× ULN<br>▪ ALT or AST >5× ULN for ≥2 weeks<br>▪ ALT or AST >3× ULN and total bilirubin >2× ULN **or** INR >1.5<br>▪ ALT or AST >3× ULN with appearance of fatigue, nausea, vomiting, right upper quadrant pain or tenderness, fever, rash, and/or eosinophilia (>5%)<br>○ Considerations: Stopping a drug at the first sign of liver enzyme elevation does not always allow for assessment of whether the liver can adapt to the drug, which could be important for drugs that cause liver injury without leading to severe DILI. However, the safety of the subject is paramount, and significant enzyme elevations or signs of liver dysfunction necessitate drug discontinuation.
**Evaluating data for alternative causes**	• Consider the importance of ruling out other causes for liver abnormalities, such as acute viral hepatitis, alcoholic and autoimmune hepatitis, hepatobiliary disorders, NASH, cardiovascular causes, and concomitant treatments, which is crucial for managing DILI.
**Follow-up to resolution**	• Continue to monitor patients until liver test abnormalities return to baseline or resolve completely.

Abbreviations: ALP, alkaline phosphatase; ALT, alanine aminotransferase; AST, aspartate aminotransferase, BIW, twice weekly, DILI, drug-induced liver injury; INR, international normalized ratio; NASH, nonalcoholic steatohepatitis; QW, weekly; TIW, thrice weekly; ULN, upper limit of normal.

Source: FDA Guidance for Industry: Drug-Induced Liver Injury: Premarketing Clinical Evaluation.

Depression/Suicidality

The C-SSRS and PHQ-9 will be utilized to assess baseline and treatment emergent depression, suicidal ideation and behavior. During the study conduct, a subject will be referred to a MHP for a PHQ-9 score of >10, any suicidal behavior, or any suicidal ideation of type 4 or 5 on the C-SSRS.

CK Elevations

To ensure subject safety and enhance reliability in determining the potential for CK elevations with apitegromab, a standardized process for identification, monitoring, and evaluation of CK elevations should be followed:

1. Throughout the study, instruct subjects to promptly report any unexplained muscle pain or weakness, particularly if associated with malaise or fever. If this occurs, measure CK as soon as possible.
2. If CK is found to be elevated >5× ULN on routine testing, question the subject about muscle symptoms.
3. A study site physician should be alerted to the occurrence of unexplained muscle symptoms and any CK >5× ULN and must take immediate action if CK is >10× ULN.
4. Perform specific questioning and follow-up investigations according to Table 13.

Table 13: Safety Monitoring Guidance for Creatine Kinase Elevations

Event	Follow-Up Monitoring
CK >5× ULN with muscle symptoms OR CK>10× ULN with or without muscle symptoms	• Clarify the nature, duration and intensity of any muscle symptoms<br>• Review possible predisposing factors, such as unaccustomed exercise, heavy alcohol intake, viral illness (consider performing serology), concomitant medications, and consider diagnosis of other conditions which can cause myopathy<br>• Conduct physical examination for muscle tenderness, weakness, and rash<br>• Measure CK again within a few days<br>• Measure serum creatinine<br>• Obtain urine dipstick<br>• Arrange to review the subject again in 4 to 10 days, or earlier if symptoms of myopathy appear or worsen, or if the urine becomes very dark<br>• Discontinue drug for CK >10× ULN with or without muscle symptoms<br>• Subjects should be followed up until resolution of the AE
CK between 5× and 10× ULN	• If asymptomatic, or if symptomatic but an alternative explanation exists, follow symptoms and CK levels weekly until there is no longer medical concern, or symptoms worsen and meet criteria in the previous row<br>• If symptomatic, and no alternative explanation exists, discontinue drug

Abbreviations: AE, adverse event; CK, creatine kinase; ULN, upper limit of normal

9.3. Common Terminology Criteria for Adverse Events/Serious Adverse Events

Clinical and laboratory AEs will be graded using the National Cancer Institute Common Terminology Criteria for Adverse Events, Version 5.0 or higher. The term "severe" is often used to describe the intensity (severity) of a specific event (as in mild, moderate, or severe myocardial infarction); the event itself, however, may be of relatively minor medical significance (such as severe nausea). This is not the same as "serious," which is based on subject/event outcome or action criteria usually associated with events that pose a threat to a subject's life or functioning.

9.4. Adverse Event Monitoring

Each subject will be monitored for the occurrence of AEs, including SAEs, from the signing of the ICF through the final follow-up visit.

Subjects will be questioned and/or examined by the Investigator or a qualified designee for evidence of AEs. The questioning of subjects with regard to the possible occurrence of AEs will be generalized such as, "How have you been feeling since your last visit?" The presence or absence of specific AEs should not be elicited from subjects.

Subjects having AEs will be monitored until resolution or stabilization (in the case of persistent impairment), or until the event becomes chronic in nature, or the subject dies.

Adverse events, actions taken as a result of AEs, and follow-up results must be recorded in the eCRF as well as in the subject's source documentation. Follow-up laboratory results should be filed with the patient's source documentation.

For any SAEs or AEs that require the subject to be discontinued from dosing, relevant clinical assessments and laboratory tests will be repeated as clinically appropriate, until final resolution or stabilization of the event(s). Subjects will continue to be followed through 20 weeks after the final dose.

All safety laboratory analyses will be performed at a central laboratory. The clinical laboratory values will be reported to the Investigator who will review them for clinical significance and consideration of abnormal values as potential AEs.

9.5. Recording Adverse Events and Serious Adverse Events

When an AE/SAE occurs, it is the responsibility of the Investigator to review all documentation (eg, hospital progress notes, laboratory reports, and diagnostics reports) related to the event. The Investigator will then record all relevant AE/SAE information.

It is not acceptable for the Investigator to send photocopies of the subject's medical records to Safety in lieu of completion of the SAE form. There may be instances when copies of medical records for certain cases are requested by Safety. In this case, all subject identifiers, with the exception of the subject number, will be redacted on the copies of the medical records before submission to Safety. The Investigator will attempt to establish a diagnosis of the event based on signs, symptoms, and/or other clinical information. Whenever possible, the diagnosis (not the individual signs/symptoms) will be documented as the AE/SAE.

There may be instances when an AE becomes an SAE. In such cases, the AE will end when the SAE starts. If an SAE becomes nonserious, the same applies: the SAE will end when the nonserious AE starts. For an AE to be serious, it needs to meet one of the regulatory seriousness criteria defined in Section 9.2.3. Hence, event start and end dates should match the start and end dates of when the criterion/criteria was/were met (eg, if a subject is hospitalized from Friday to Sunday, the start date will be Friday and the end date will be Sunday). If the Investigator feels that the event also met another seriousness criterion outside of the hospitalization dates, the event start and end dates may be different than the admission and discharge dates as the extra days are covered by the additional seriousness criterion.

9.5.1. Assessment of Intensity

The Investigator will assess intensity for each AE and SAE reported during the study according to the NCI CTCAE Grading Scale, Version 5.0 or higher (NIH 2023). Only AEs not listed in the CTCAE should be graded as summarized in Table 14.

Table 14: Adverse Event and Serious Adverse Event Assessment of Intensity

CTCAE Grade	Equivalent To	Definition
Grade 1	Mild	Mild; asymptomatic or mild symptoms; clinical or diagnostic observations only; intervention not indicated
Grade 2	Moderate	Moderate; minimal, local or noninvasive intervention indicated; limiting age-appropriate instrumental ADL[a]
Grade 3	Severe	Severe or medically significant but not immediately life-threatening; hospitalization or prolongation of hospitalization indicated; disabling; limiting self-care ADL[b]
Grade 4	Life-threatening/disabling	Life-threatening consequences; urgent intervention indicated
Grade 5	Death	Death related to AE

Abbreviations: ADL, activities of daily living; AE, adverse event, CTCAE, Common Terminology Criteria for Adverse Events.
Semi-colon indicates "or" within the description of the grade.
a Instrumental ADL refer to preparing meals, shopping for groceries or clothes, using the telephone, managing money, etc.
b Self-care ADL refer to bathing, dressing and undressing, feeding self, using the toilet, taking medications, and not bedridden.

9.5.2. Assessment of Causality

A medically qualified Investigator must assess the relationship of any AE (including SAEs) to the use of the study drug, as related or not related, based on clinical judgment and using all available information. A "reasonable possibility" of a relationship conveys that there are facts, evidence, and/or arguments to suggest a causal relationship, rather than a relationship cannot be ruled out.

The Investigator will use clinical judgment to determine the relationship. Alternative causes, such as underlying disease(s), concomitant therapy, and other risk factors, as well as the

temporal relationship of the event to study drug administration will be considered and investigated.

The causal relationship between the study drug and AE/SAE will be assessed to be one of the following:

- Not Related
    - Temporal relationship is lacking (eg, event did not occur within a reasonable timeframe following administration of study drug)
    - Other causative factors more likely explain the AE/SAE (eg, pre-existing condition, other concomitant medications)
- Related
    - There is a positive temporal relationship (eg, event occurred within a reasonable timeframe following administration of study drug)
    - The AE/SAE is more likely explained by the study drug than by other causes (the AE shows a pattern consistent with previous knowledge of the study drug or the class of the study drug)

For each AE/SAE, the Investigator must document in the medical notes that he/she has reviewed the AE/SAE and has provided an assessment of causality. There may be situations in which an SAE has occurred, and the Investigator has minimal information. However, it is very important that the Investigator always assess causality for every event.

The Investigator may change his/her opinion of causality in light of follow-up information and send an SAE follow-up report with the updated causality assessment. The causality assessment is one of the criteria used when determining regulatory reporting requirements.

9.5.3. Assessment of Expectedness

As part of the regulatory reporting requirements, the Sponsor must perform an assessment of expectedness (expected/unexpected from the perspective of previously observed, not on the basis of what might be anticipated from the pharmacological properties of a study drug) for AEs. Adverse reactions will be considered unexpected if the nature, seriousness, severity or outcome of the reaction(s) is not consistent with the reference safety information section of the apitegromab (SRK-015) Investigator's Brochure for CM Obesity.

9.5.4. Instructions for Recording AEs in eCRF

9.5.4.1. Recording Diagnosis Versus Signs and Symptoms

If a subject reports signs and symptoms that represent a medical diagnosis/syndrome, the final diagnosis/syndrome should be recorded in the eCRF rather than each sign and symptom (eg, cough, runny nose, fever = upper respiratory infection). However, if a medical diagnosis cannot be made, then each sign or symptom should be recorded as an individual SAE or AE, as appropriate.

9.5.4.2. Abnormal Laboratory Values or Vital Signs

Protocol-defined laboratory values and vital signs should not be reported as AEs unless the abnormal laboratory value or vital sign meets at least one of the following:

- Requires an adjustment in the study drug(s) or discontinuation of treatment;
- Requires medical or surgical intervention;
- Is associated with accompanying signs/symptoms that are not considered part of a pre-existing diagnosis or syndrome (or if considered part of a pre-existing diagnosis or syndrome, is associated with disease worsening); or
- Is considered clinically significant by the Investigator

9.6. Reporting Serious Adverse Events or AESIs

9.6.1. Reporting to Safety Using an Electronic Data Collection Tool

The primary mechanism for reporting an SAE or AESIs to Safety will be the electronic data collection (EDC) tool. The SAE/AESI will be reported regardless of whether it is serious or non-serious and regardless of causality. The process is as follows:

- The site will enter the SAE/AESIs data into the electronic system as soon as it becomes available.
- After the study is completed at a given site, the EDC will be taken off-line to prevent the entry of new data or changes to existing data.
- If a site receives a report of a new SAE/AESI from a study subject or receives updated data on a previously reported SAE/AESI after the EDC has been taken off-line, then the site can report this information on a paper SAE/AESI form.
- Contacts for SAE/AESI reporting can be found in the SAE/AESI form, in the site binder.

9.6.2. Reporting to Safety Using a Paper SAE/AESI Form

If the electronic system is unavailable, then the site will use the paper SAE/AESI form to report the event within 24 hours. Non-serious AESIs will also be reported within 24 hours on an SAE form using the email address provided in the site binder. The backup method for SAE/AESI reporting is via email using the email address provided in the site binder. Should technical issues not allow for email transmission of the SAE/AESI Report Form, the form may be submitted using the fax number provided in the site binder.

In rare circumstances and in the absence of facsimile equipment, notification by telephone is acceptable with a copy of the SAE/AESI data collection form sent by overnight mail or courier service.

Initial notification via telephone does not replace the need for the Investigator to complete and sign the SAE/AESI data collection form within the designated reporting time frames.

Contacts for SAE/AESI reporting can be found in the SAE/AESI form, in the site binder.

9.7. Time Period and Frequency for Collecting AE, SAE, and AESI Information

All AEs, including SAEs, and AESIs will be collected from the signing of the ICF until the final follow-up visit at the time points specified in Table 1.

During Screening, subjects may undergo more examinations and tests than they have in the past, and therefore new findings may be detected. Medical occurrences that begin before the start of study drug but after obtaining informed consent will be recorded as Medical History/Current Medical Conditions, not as AEs. In other words, if it is reasonable that the finding started/developed before obtaining informed consent, it should be classified as Medical History/Current Medical Condition. Otherwise, or if there is no way to determine, it is an AE.

All SAEs and AESIs will be recorded and reported immediately and under no circumstance should this exceed 24 hours. The Investigator will submit any updated SAE/AESI data within 24 hours of it being available. Investigators are not obligated to actively seek information on AEs or SAEs after conclusion of the study participation. However, if the Investigator learns of any SAE, including a death, at any time after a subject has been discharged from the study, and he/she considers the event to be reasonably related to the study drug or study participation, the Investigator must promptly notify the Sponsor.

9.8. Follow-up of AEs, SAEs, and AESIs

After the initial AE/SAE/AESI report, the Investigator is required to proactively follow each subject at subsequent visits/contacts. The Investigator is obligated to perform or arrange for the conduct of supplemental measurements and/or evaluations as medically indicated or as requested by the Sponsor's Safety and Pharmacovigilance group to elucidate the nature and/or causality of the AE or SAE/AESI as fully as possible. This may include additional laboratory tests or investigations, histopathological examinations, or consultation with other health care professionals.

If a subject dies during participation in the trial or during a recognized follow-up period, the Investigator will provide to the Sponsor's Safety and Pharmacovigilance group with a copy of any post-mortem findings including histopathology. New or updated information will be recorded in the originally submitted documents. The Investigator will submit any updated SAE/AESI data to Safety within 24 hours of receipt of the information.

All SAEs will be followed until resolution, stabilization, the event is otherwise explained, or the subject is lost to follow-up.

9.8.1. Regulatory Reporting Requirements for SAEs/AESIs

Prompt notification by the Investigator to the Sponsor of an SAE/AESI is essential so that legal obligations and ethical responsibilities toward the safety of subjects and the safety of a study drug under clinical investigation are met.

The Sponsor has a legal responsibility to notify both the local regulatory authority and other regulatory agencies about the safety of a study drug under clinical investigation. The Sponsor will comply with country-specific regulatory requirements relating to safety reporting to the regulatory authority, IRB, and Investigators.

An Investigator who receives an Investigator safety report describing an SAE/AESI or other specific safety information (eg, summary or listing of SAEs/AESIs) from the Sponsor will review and then file it along with the Investigator's Brochure and will notify the IRB/IEC, if appropriate according to local requirements.

Investigator safety reports must be prepared for suspected unexpected serious adverse reactions according to local regulatory requirements and Sponsor policy and forwarded to Investigators as necessary.

9.9. Contraceptive and Barrier Guidance

9.9.1. Definitions

A female is considered to be of childbearing potential after menarche and until becoming postmenopausal, unless permanently sterile.

Permanent sterilization methods include hysterectomy, bilateral salpingectomy, and bilateral oophorectomy.

A postmenopausal state is defined as no menses for 12 months without an alternative medical cause.

9.9.2. Contraception Guidance

The effects of apitegromab on conception, pregnancy, and lactation are unknown.

No changes in reproductive organs were noted during rat and monkey repeat-dose toxicology studies and no changes in reproductive organs, sperm parameters, or fertility were observed in a juvenile rat toxicology study. An increase in mean estrous cycle length in females and a decrease in sperm concentration in males was noted during a study on rat fertility and early embryonic development; however, these changes had no effect on mating or fertility indices. Increases in mean maternal and/or fetal body weights were also observed in rabbit and rat embryo/fetal development studies that had no impact on maternal well-being or embryo-fetal development.

No effects in skeletal variations or developmental malformations were observed in these studies. The long-term effects of these changes on reproductive potential are unknown.

Due to potential risks to fetus when using incretin mimetic therapy, the female subjects of childbearing potential must use 1 or more highly effective method(s) of contraception that is/are not user dependent or 2 highly effective contraception methods that are user dependent (Table 15) throughout the clinical study and for 20 weeks after the last dose of study drug. The use of contraceptive methods is not required if the male partner (of the female subject) has a documented history of a vasectomy or if the female subject has a documented history of hysterectomy, bilateral salpingectomy, or bilateral oophorectomy, or if she is postmenopausal for at least 12 months.

Male subjects are not required to use contraceptive methods.

However, all subjects should be reminded to use the most conservative method of contraception that is consistent with the local prescribing information (eg, SmPC, FDA Principal Investigator) for their concomitant medications.

9.9.3. Acceptable Methods of Contraception

Acceptable methods of contraception allowed during the study are listed in Table 15.

Use of incretin mimetic therapy may reduce the efficacy of oral hormonal contraceptives due to delayed gastric emptying. Patients using oral hormonal contraceptives should switch to a non-oral contraceptive method and/or add a barrier method of contraception for 4 weeks after initiation with incretin mimetic therapy and for 4 weeks after each dose escalation.

Table 15: Methods of Contraception Allowed During the Study

**Highly Effective Methods[a] That Have Low User Dependency[b]**
• Implantable progestogen-only hormone contraception associated with inhibition of ovulation[c]<br>• Intrauterine device<br>• Intrauterine hormone-releasing system<br>• Bilateral tubal occlusion
**Highly Effective Methods[a] That Are User Dependent**
• Combined (estrogen- and progestogen-containing) hormonal contraception associated with inhibition of ovulation[c]<br>○ Oral<br>○ Intravaginal<br>○ Transdermal<br>• Progestogen-only hormone contraception associated with inhibition of ovulation[c]<br>○ Oral<br>○ Injectable<br>• Sexual abstinence<br>○ *Sexual abstinence is considered a highly effective method only if defined as refraining from heterosexual intercourse during the entire period of risk associated with the study drug. The reliability of sexual abstinence needs to be evaluated in relation to the duration of the study and the preferred and usual lifestyle of the subject.*

a Failure rate of <1% per year when used consistently and correctly. Typical use failure rates differ from those when used consistently and correctly.

b These methods of contraception, which are considered to have low user dependency, should preferably be used, in particular, when contraception is introduced as a result of participation in the clinical study.

c If locally required, in accordance with Clinical Trial Facilitation Group guidelines, acceptable contraceptive methods are limited to those which inhibit ovulation as the primary mode of action.

10. STATISTICS

Throughout this section, treatment group refers to the randomization arm (ie, apitegromab + incretin mimetic therapy or placebo + incretin mimetic therapy).

10.1. Statistical Hypotheses

Let μ_p and μ_a stand for the average change from Baseline in LBM (measured in kg) at Week 24 for subjects in the placebo + incretin mimetic therapy group and the apitegromab + incretin mimetic therapy group, respectively. The null hypothesis of primary interest of the study is $\mu_p \geq \mu_a$ against the alterative hypothesis $\mu_p < \mu_a$.

Analyses are considered descriptive, and no formal hypothesis testing will be conducted. For this proof-of-concept study, any hypothesis testing should be considered exploratory.

10.2. Sample Size Determination

The estimated difference between the apitegromab + incretin mimetic therapy group and the placebo + incretin mimetic therapy group in mean change from Baseline of LBM at Week 24 will be reported together with a 2-sided 80% confidence interval. Assuming the change follows an approximately normal distribution with a standard deviation of 4.6 kg, a sample size of 50 subjects per arm yields approximately 80% power to detect an effect size of 2 kg. If approximately 13% of subjects are not evaluable for an analysis, a sample size of 43 subjects per arm yields approximately 75% power to detect an effect size of 2 kg.

10.3. Analysis and Populations

Unless otherwise specified, the efficacy outcome analyses will be conducted on the Intention-to-Treat (ITT) population, defined as all randomized subjects, even if the subject does not receive the correct treatment, or otherwise did not follow the protocol. Subjects will be analyzed according to the treatment group to which they were assigned.

Safety analyses will be done on the Safety population, which includes all randomized subjects who receive at least 1 dose of study drug.

Further details and other populations will be described in the SAP.

10.4. Statistical Analyses

The SAP will be finalized prior to the unblinding of treatment assignment, and it will include a more technical and detailed description of the statistical analyses described in this section. This section is a summary of the planned statistical analyses of the most important endpoints. Changes in the analysis methods before the database lock that are not part of a protocol amendment will be delineated in the SAP, and any deviations from the SAP will be documented in the clinical study report.

10.4.1. General Considerations

A statistical analysis of this study will be the responsibility of Scholar Rock or its designee. A detailed SAP describing the statistical methodologies will be developed by Scholar Rock or its designee.

Unless otherwise specified, all analyses will be conducted using Statistical Analysis System (Version 9.4 or later, Cary, North Carolina).

Baseline for all efficacy and safety variables will be defined as the last non-missing measurement prior to the first dose of study drug.

Important intercurrent events in this study include 1) permanent early discontinuation of study treatment, or 2) modification of planned study treatment. Additional intercurrent events may be delineated in the SAP.

10.4.2. Primary Efficacy Endpoint Analysis

The efficacy of apitegromab + incretin mimetic therapy will be assessed by the change from Baseline in LBM (kg) at Week 24 compared with placebo + incretin mimetic therapy in the ITT population.

The difference between treatment groups in change from Baseline in LBM at Week 24 will be estimated together with a 2-sided 80% confidence interval using a linear regression model, controlling for Baseline weight, Baseline LBM, age, and sex.

The primary analysis will utilize a treatment policy strategy for handling intercurrent events and will include all values regardless of intercurrent events or protocol deviations. All subjects in the ITT population will be included and analyzed according to randomized treatment group.

In the primary analysis, missing data for LBM at Week 24 will be imputed as the body weight measurement closest to Day 169 (ie, the target day for the Week 24 visit) multiplied by the subject’s Baseline LBM percentage. If necessary, imputation for missing Baseline LBM or missing Baseline regression covariates will be specified in the SAP.

Sensitivity analyses may involve including different covariates in the regression model, implementing different methods for handling missing data or other variations on the primary analysis and will be described in the SAP. Alternate strategies for handling intercurrent events may be explored and will be described in the SAP.

10.4.3. Secondary and Exploratory Efficacy Endpoint Analysis

All efficacy variables will be summarized by visit and treatment group in the ITT population. For designated efficacy endpoints and visits, the difference in mean change from Baseline between treatment groups will be estimated using a regression model appropriate for the distribution of the endpoint, controlling for selected Baseline characteristics. In addition, correlation between efficacy endpoints will be explored. Details will be provided in the SAP.

10.4.4. Multiplicity

For this proof-of-concept study, any hypothesis testing is considered exploratory. No adjustment for multiple comparisons will be applied.

10.4.5. Safety Endpoints Analyses

Safety data will be summarized descriptively by treatment group and analyzed using the Safety population.

AEs will be analyzed based on the principle of treatment-emergence. TEAEs are defined as AEs that started or worsened in severity after the first dose of study drug. Only TEAEs will be included in summaries; any reported predose AEs will be listed separately. AEs will be coded using the Medical Dictionary for Regulatory Affairs (MedDRA) and summarized using MedDRA hierarchies as described in the SAP. The incidence of TEAEs, serious TEAEs and TEAEs leading to treatment discontinuation will be summarized; additional analyses of TEAEs (eg, by severity) will be detailed in the SAP.

Shifts from Baseline in laboratory parameters, vital signs, ECGs, and depression severity (as assessed by the PHQ-9) will be summarized. Findings from the C-SSRS will be summarized.

10.4.6. Pharmacokinetic and Pharmacodynamic Endpoints Analyses

All subjects who receive at least 1 dose of study drug and have at least 1 quantifiable PK result will be included in the PK analysis. Apitegromab concentrations and circulating latent myostatin concentrations will be summarized by visit. The apitegromab and latent myostatin concentrations from this study may be combined with other study data to support the population PK and PK/PD analyses.

10.4.7. Anti-apitegromab Antibody Endpoint Analyses

All subjects who receive at least one dose of study drug and have at least 1 ADA result will be included in the immunogenicity evaluation. The number and percentage of subjects who become positive for ADAs and who develop neutralizing antibodies will be summarized by Baseline ADA status (negative, positive, or missing). Antibody results for subjects with any positive value will be listed.

10.4.8. Additional Endpoints Analyses

Analyses for additional endpoints will be specified in the SAP.

10.4.9. Subgroup Analyses

Subgroup analyses by various factors (such as type of incretin mimetic therapy or selected Baseline characteristics) may be performed to explore whether the treatment effect and/or safety profile of study drug is similar across subgroups. Subgroup analyses will be described in the SAP.

10.5. Interim Analysis

No interim analysis will be conducted. At the end of the Treatment Period, the study team will be unblinded and the primary efficacy analysis will be performed. The Investigator and the subjects will remain blinded until after the Safety Follow-up period is completed; details will be provided in the unblinding plan.

11. DIRECT ACCESS TO SOURCE DATA/DOCUMENTS

11.1. Study Monitoring

Before an investigational site can enter a subject into the study, an authorized representative of Scholar Rock, Inc. will visit the investigational study site to:

- Determine the adequacy of the facilities
- Discuss with the Investigator(s) and other personnel their responsibilities with regard to protocol adherence, and the responsibilities of Scholar Rock, Inc. or its representatives. This will be documented in a Clinical Study Agreement between Scholar Rock, Inc. and the Investigator.

During the study, a monitor from Scholar Rock, Inc. or authorized representative will have regular contacts with the investigational site, for the following:

- Provide information and support to the Investigator(s)
- Confirm that facilities remain acceptable
- Confirm that the investigational team is adhering to the protocol, that data are being accurately recorded in the case report forms, and that investigational product accountability checks are being performed
- Perform source data verification. This includes a comparison of the data in the case report forms with the subject's medical records at the hospital or practice, and other records relevant to the study. This will require direct access to all original records for each subject (eg, clinic charts).
- Record and report any protocol deviations not previously sent to Scholar Rock, Inc.
- Confirm AEs and SAEs have been properly documented on CRFs and confirm any SAEs have been forwarded to Scholar Rock, Inc. and those SAEs that met criteria for reporting have been forwarded to the IRB.

The monitor will be available between visits if the Investigator(s) or other staff needs information or advice.

11.2. Audits and Inspections

Authorized representatives of Scholar Rock, Inc., a regulatory authority, an IRB may visit the site to perform audits or inspections, including source data verification. The purpose of a Scholar Rock, Inc. audit or inspection is to systematically and independently examine all study-related activities and documents to determine whether these activities were conducted, and data were recorded, analyzed, and accurately reported according to the protocol, GCP guidelines of the ICH, and any applicable regulatory requirements. The Investigator should contact Scholar Rock, Inc. immediately if contacted by a regulatory agency about an inspection.

11.3. Institutional Review Board/Independent Ethics Committee

The Investigator must obtain IRB approval for the clinical study. Initial IRB approval, subsequent amendments, and all materials approved by the IRB for this study including the subject consent form and recruitment materials must be maintained by the Investigator and made available for inspection.

12. QUALITY CONTROL AND QUALITY ASSURANCE

To ensure compliance with ICH/GCP and all applicable regulatory requirements, Scholar Rock, Inc. may conduct a quality assurance audit. Please see Section 11.2 for more details regarding the audit process.

12.1. Protocol Adherence

The Investigator will conduct the study in accordance with the protocol. Deviations must be documented and will be reviewed by the Sponsor. Prospective approval of protocol deviations, also known as protocol waivers or exemptions, is not permitted.

13. ETHICS

13.1. Ethics Review

The final study protocol, including the final version of the ICF, must be approved or given a favorable opinion in writing by an IRB, as appropriate. The Investigator must submit written IRB approval to Scholar Rock, Inc. before he or she can recruit any subject into the study.

The Investigator is responsible for informing the IRB of any amendment to the protocol in accordance with local requirements. In addition, the IRB must approve all advertising used to recruit subjects for the study. The protocol must be re-approved by the IRB upon receipt of amendments and annually, as local regulations require.

The Investigator is also responsible for providing the IRB with reports of any reportable serious ADRs from any other study conducted with the investigational product. Scholar Rock, Inc. will provide this information to the Investigator.

Progress reports and notifications of serious ADRs will be provided to the IRB according to local regulations and guidelines.

13.2. Ethical Conduct of the Study

The study will be performed in accordance with ethical principles that have their origin in the Declaration of Helsinki (Appendix 1 Section 17.1) and are consistent with ICH/GCP, applicable regulatory requirements and the Scholar Rock, Inc.'s policy on Bioethics.

13.3. Written Informed Consent

The informed consent process is described in full in Section 17.1.3. The Investigator(s) at each study site will ensure that the subject is given full and adequate oral and written information

about the nature, purpose, possible risk and benefit of the study. Subjects must also be notified that they are free to discontinue from the study at any time. The subject should be given the opportunity to ask questions and allowed time to consider the information provided.

The subject's signed and dated informed consent must be obtained before conducting any study procedures.

The Investigator(s) must maintain the original, signed, and dated ICF. A copy of the signed ICF must be given to the subject.

14. DATA HANDLING AND RECORDKEEPING

14.1. Inspection of Records

Scholar Rock, Inc. and its authorized representatives will be permitted to examine clinical records for the evaluation of study safety, progress, and data validity, audits, and quality assurance reviews. The investigator/institution should make available for direct access all requested study-related records for Sponsor examination, IRB review, and regulatory inspection.

Scholar Rock, Inc. and its authorized representatives will be allowed to conduct site visits to the facilities for the purpose of monitoring any aspect of the study. The Investigator agrees to allow the monitor to inspect the drug storage area, study drug stocks, drug accountability records, subject charts and study source documents, and other records relative to study conduct.

14.2. Retention of Records

The Investigator must maintain all documentation relating to the study for a period of 2 years after the last marketing application approval, or if not approved 2 years following the discontinuance of the investigational product for investigation. If it becomes necessary for Scholar Rock, Inc. or the regulatory authority to review any documentation relating to the study, the Investigator must permit access to such records.

Investigator must obtain written approval from the Sponsor, prior to transfer or destruction of any study records, data, or media.

15. PUBLICATION POLICY

The information obtained in the conduct of this study is the confidential information of the Sponsor and is subject to the terms of the study center's Clinical Trial Agreement with the Sponsor.

The results of this study may be published or presented by the Sponsor at scientific meetings. The Sponsor will comply with the requirements for publication of study results. In accordance with standard editorial and ethical practice, the Sponsor will generally support publication of multi-site studies only in their entirety and not as individual site data.

Authorship will be determined by mutual agreement and in line with International Committee of Medical Journal Editors authorship requirements.

16. LIST OF REFERENCES

Acosta A, Streett S, Kroh MD, Cheskin LJ, Saunders KH, Kurian M, et al. White Paper AGA: POWER — Practice Guide on Obesity and Weight Management, Education, and Resources. *Clin Gastroenterology and Hepatology*. 2017;15(5):631-649.

Aristizabal JC, Freidenreich DJ, Volk BM, Kupchak BR, Saenz C, Maresh CM, et al. Effect of resistance training on resting metabolic rate and its estimation by a dual-energy X-ray absorptiometry metabolic map. *Eur J Clin Nutr*.2015;69: 831–836. https://doi.org/10.1038/ejcn.2014.216.

Aronne LJ, Sattar N, Horn DB, Bays HE, Wharton S, Lin W-Y, et al. Continued treatment with tirzepatide for maintenance of weight reduction in adults with obesity. The SURMOUNT-4 Randomized Clinical Trial. *JAMA*. 2024;331(1):38-48.

Bergmann NC, Davies MJ, Lingvay I, Knop FK. Semaglutide for the treatment of overweight and obesity: A review. *Diabetes Obes Metab*. 2023(Jan);25(1):18-35.

Bilic et al. Comparison of predicted efficacy between 10 mg/kg and 20 mg/kg of apitegromab in spinal muscular atrophy patients aged from 2 to 12 years of age. *Modeling and Simulation Analysis Memo*. 2023.

Campbell C, McMillan HJ, Mah JK, Tarnopolsky M, Selby K, McClur T, et al. Myostatin inhibitor ACE-031 treatment of ambulatory boys with Duchenne muscular dystrophy: Results of a randomized, placebo-controlled clinical trial. *Muscle Nerve*. 2017 Apr;55(4):458-464.

Casati M, Costa AS, Capitanio D, Ponzoni L, Ferri E, Agostini S, et al. The Biological Foundations of Sarcopenia: Established and Promising Markers. *Front Med (Lausanne)*. 2019 Aug 13:6:184.

Centers for Disease Control. Accessed 10 October 2023. https://www.cdc.gov/nchs/data/nhanes/nhanes_07_08/manual_an.pdf

Coelho M, Oliveira T, Fernandes R. Biochemistry of adipose tissue: an endocrine organ. *Arch Med Sci*. 2013;9(2): 191-200. 2013.

Crawford TO, Darras BT, Day JW, et al. Effect of apitegromab on motor function and PEDICAT and PROMIS at 36-months in patients with type 2 nonambulatory type 3 spinal muscular atrophy combined presentation of the phase 2 TOPAZ study. 2023 Annual SMA Conference. https://scholarrock.com/wp-content/uploads/2023/06/FINAL_TOPAZ-CureSMA-combined-PRO-and-MOTOR-at-36m_vFINAL_063023_Reformatted.pdf

Columbia-Suicide Severity Rating Scale. Accessed 29 January 2024. https://cssrs.columbia.edu/

Czernichow S, Kengne AP, Stamatakis E. Body mass index, waist circumference, and waist-hip ratio: which is the better discriminator of cardiovascular disease mortality risk? Evidence from an individual-participant meta-analysis of 82, 864 participants from nine cohort studies. *Obes Rev*. 2011. 12(9): 680-687.

Cunningham JM. Updated recommendations for the treatment of immune thrombocytopenia. *Clin Adv Hematol & Oncol*. 2020(August);18(8):442-446.

Day JW, Howell K, Place A, Long K, Rossello J, Kertesz N, et al. Advances and limitations for the treatment of spinal muscular atrophy. *BCM Pediatrics*. 2022;22:632.

Friedl KE, Moore RJ, Hoyt RW, Marchitelli LJ, Martinez-Lopez LE, Askew EW. Endocrine markers of semistarvation in healthy lean men in a multistressor environment. *J Appl Physiol* (1985). 2000 May;88(5):1820-30.

Fukushima Y, Kurose S, Shinno H, Thu HC, Takao N, Tsutsumi H, et al. Importance of lean muscle maintenance to improve insulin resistance by body weight reduction in female patients with obesity. *Diabetes Metab J*. 2016;40: 147-153.

Garito T, Zakaria M, Papanicolaou DA, Li Y, Pinot P, Petricoul O, et al. Effects of bimagrumab, an activin receptor type II inhibitor, on pituitary neurohormonal axes. *Clin Endocrinol (Oxf)*. 2018(Jun);88(6):908-919.

Garvey, WT, Lewington S, Sherliker P, Clarke R, Emberson J, Halsey J, et al. Body-mass index and cause-specific mortality in 900,000 adults: collaborative analyses of 57 prospective studies. *Lancet.* 2009;373;1083-96.

Ghusn W, De la Rosa A, Sacoto D, Cifuentes L, Campos A, Feris F, et al. Weight loss outcomes associated with semaglutide treatment for patients with overweight or obesity. *JAMA*. 2022(Sep 1);5(9):e2231982.

Goh LGH, Dhaliwal SS, Welborn TA, Lee AH, Della PR. Anthropometric measurements of general and central obesity and the prediction of cardiovascular disease risk in women: a cross-sectional study. *BMJ Open*. 2014;4: e004138.

Harman SM, Blackman MR. Use of growth hormone for prevention or treatment of effects of aging. *J Gerontol A Biol Sci Med Sci*. 2004 Jul;59(7):652-8

Heymsfield SB, Coleman LA, Miller R, Rooks DS, Laurent D, Petricoul O, et al. Effect of bimagrumab versus placebo on body fat mass among adults with type 2 diabetes and obesity. *JAMA Network Open*. 2021;4(1):e2033457.

Ida S, Kaneko R, Imataka K, Okubo K, Shirakura Y, Azuma K, et al. Effects of antidiabetic drugs on muscle mass in type 2 diabetes mellitus. *Curr Diabetes Rev*. 2021;17:293-303.

Jastreboff AM, Aronne JL, Ahmad NN, et al. Tirzepatide once weekly for the treatment of obesity. New Eng J Med. 2022(July 22);387(3):205-216.

Jensen MD, Ryan DH, Apovian CM, Ard JD, Comuzzie AG, Donato KA, et al. 2013 AHA/ACC/TOS guideline for the management of overweight and obesity in adults: a report of the American College of Cardiology/American Heart Association Task Force on Practice Guidelines and The Obesity Society. *J Am Coll Cardiol*. 2014;63(Pt B):2985–3023.

Joshi GP, Abdelmalak BB, Weigel WA, Soriano SG, Harbell MW, Kuo CI, et al. American Society of Anesthesiologists consensus-based guidance on preoperative management of patients (adults and children) on glucagon-like peptide-1 (GLP-1) receptor agonists. American Society of Anesthesiologists Press Release. 29 June 2023. Accessed 05 September 2023. https://www.asahq.org/about-asa/newsroom/news-releases/2023/06/american-society-of-anesthesiologists-consensus-based-guidance-on-preoperative.

Klein SR, Hobai IA. Semaglutide, delayed gastric emptying, and intraoperative pulmonary aspiration: A case report. *Can J Anesth*. 2023.

Kroenke K, Spitzer RL, Williams JBW. The PHQ-9. *J Gen Inter Med*. 2001;16: 606-613. https://doi.org/10.1046/j.1525-1497.2001.016009606.x.

Kobori T, Onishi Y, Yoshida Y, Tahara T, Kikuchi T, Kubota T, et al. Association of glucagon-like peptide-1 receptor agonist treatment with gastric residue in an esophagogastroduodenoscopy. *J Diabetes Investig*. 2023;14:767-73.

Ligibel JA, Alfano CM, Courneya KS, Denmark-Wahnefried W, Burger RA, Chlebowski RT, et al. American Society of Clinical Oncology position statement on obesity and cancer. *J Clin Oncol*. 2014;32: 3568-3574

Lindegaard B, Hansen T, Hvid T, van Hall G, Plomgaard P, Ditlevsen S, et al. The effect of strength and endurance training on insulin sensitivity and fat distribution in human immunodeficiency virus-infected patients with lipodystrophy. *J Clin Endocrinol Metab*. 2008;93:3860–9.

Long KK, O'Shea KM, Khairallah RJ, Howell K, Paushkin S, Chen KS, et al. Specific inhibition of myostatin activation is beneficial in mouse models of SMA therapy. *Hum Mol Genet*. 2019;28(7):1076-1089.

Mercuri E, Barisic N, Boespflug-Tanguy O, et al. SUNFISH Part 2: Efficacy and safety of risdiplam (RG7916) in patients with Type 2 or non-ambulant Type 3 spinal muscular atrophy (SMA). 2nd International Scientific and Clinical Congress on Spinal Muscular Atrophy. February 5-7, 2020. Evry, France.

National Institutes of Health. Clinical Guidelines on the Identification, Evaluation, and Treatment of Overweight and Obesity in Adults. The Evidence Report. NIH Publication No. 98-4086. September 1998.

National Institutes of Health. Overweight and Obesity. Accessed 5 December 2023 https://www.nhlbi.nih.gov/health/overweight-and-obesity.

Obesity Medicine Association. Obesity Algorithm®. 2021.

Pirruccello-Straub M, Jackson J, Wawersik S, Webster MT, Salta L, Long K, et al. Blocking extracellular activation of myostatin as a strategy for treating muscle wasting. *Sci Rep*. 2018;8:2292.

Posner K, Brown GK, Stanley B, et al. The Columbia-Suicide Severity Rating Scale: initial validity and internal consistency findings from three multisite studies with adolescents and adults. *Amer J Psychiatry* 2011;168(12):1266-77.

Prado CMM, Heymsfield SB. Lean tissue imaging: a new era for nutritional assessment and intervention. *JPEN J Parenter Enteral Nutr*. 2014;38: 940-953.

Roh E, Choi KM. Health consequences of sarcopenic obesity: a narrative review. *Front. Endocrinol*. 2020;11: 332.

Romero-Corral A, Somers VK, Sierra-Johnson J, Thomas RJ, Collazo-Clavell ML, Korinek J, et al. Accuracy of body mass index to diagnose obesity in the US adult population. *In J Obes.* 2008; 32(6); 959-966.

Rubino D, Abrahamsson N, Davies M, Hesse D, Greenway FL, Jensen C, et al. Effect of continued weekly subcutaneous semaglutide vs placebo on weight loss maintenance in adults with overweight or obesity: the STEP 4 randomized clinical trial. *JAMA*. 2021;325:1414-1425.

Sargeant JA, Henson J, King JA, Yates T, Khunti K, Davies MJ. A Review of the Effects of Glucagon-Like Peptide-1 Receptor Agonists and Sodium-Glucose Cotransporter 2 Inhibitors on Lean Body Mass in Humans. *Endocrinol Metab (Seoul)*. 2019;34:247-262

Shepherd JA, Ng BK, Sommer MJ, Heymsfield SB. Body Composition by DXA. *Bone*. 2017;104: 101-105.

Silveira SQ, da Silva LM, Abib ACV, de Moura DTH, de Moura EGH, Santos LB, et al. Relationship between perioperative semaglutide use and residual gastric content: A retrospective analysis of patients undergoing elective upper endoscopy. *J Clin Anesth*. 2023;87:111091.

Sjöström L, Rissanen A, Andersen T, Boldrin M, Golay A, Koppeschaar HP, et al. Randomised placebo-controlled trial of orlistat for weight loss and prevention of weight regain in obese patients. European Multicentre Orlistat Study Group. *Lancet.* 1998;352:167-172.

Smith SR, Weissman NJ, Anderson CM, Sanchez M, Chuang E, Stubbe S, et al. Multicenter, placebo-controlled trial of lorcaserin for weight management. *N Engl J Med.* 2010; 363: 245-256.

Srikanthan P, Karlamangla AS. Relative muscle mass is inversely associated with insulin resistance and prediabetes. Findings from the third National Health and Nutrition Examination Survey. *J Clin Endocrinol Metab*. 2011;96:2898–903.

TOPAZ Interim CSR, 11 August 2023.

Volpi E, Nazemi R, Fujita S. Muscle tissue changes with aging. *Curr Opin Clin Nutr Metab Care*. 2004;7(4): 405-410.

WEGOVY (semaglutide). US Package insert. Novo Nordisk. Accessed February 15, 2024. https://www.novo-pi.com/wegovy.pdf.

Weiss T, Carr RD, Pal S, Yang L, Sawhney B, Boggs R, et al. Real-world adherence and discontinuation of glucagon-like peptide-1 receptor agonists therapy in type 2 diabetes mellitus patients in the United States. *Patient Prefer Adherence*. 2020(Nov)27;14:2337-2345.

Wewege MA, Desai I, Honey C, Coorie B, Jones MD, Clifford BK, et al. The effect of resistance training in healthy adults on Body fat percentage, fat mass and visceral fat: A systematic review and meta-analysis. *Sports Med*. 2022(Feb);52(2):287-300.

Wilding JP, Batterham RL, Calanna S, Davies M, Van Gaal LF, Lingvay I, et al. Once-weekly semaglutide in adults with overweight or obesity. *N Engl J Med*. 2021;384(11):989–1002.

Wilding JP, Batterham RL, Davies M, Van Gaal LF, Kandler K, Konakli K, et al. Weight regain and cardiometabolic effects after withdrawal of semaglutide: The STEP 1 trial extension. *Diabetes Obes Metab*. 2022;24:1553-1564.

World Health Organization. Accessed October 16, 2023. https://www.who.int/news-room/fact-sheets/detail/obesity-and-overweight.

Yu JG, Liu JX, Carlsson L, Thornell LE, Stål PS. Re-Evaluation of Sarcolemma Injury and Muscle Swelling in Human Skeletal Muscles after Eccentric Exercise. *PLoS One*. 2013 Apr 15;8(4):e62056.

ZEPBOUND (tirzepatide). US Package insert. Lilly. Accessed February 15, 2024. https://uspl.lilly.com/zepbound/zepbound.html#pi.

Zurlo, F, Larson, K, Bogardus, C, Ravussin E. Skeletal muscle metabolism is a major determinant of resting energy expenditure. *J Clin Invest*. 1990;86(5), 1423-1427.

17. APPENDICES

17.1. Appendix 1: Regulatory, Ethical, and Study Oversight Considerations

17.1.1. Regulatory and Ethical Considerations

This study will be conducted in accordance with the protocol and with the following:

- Consensus ethical principles derived from international guidelines including the Declaration of Helsinki and Council for International Organizations of Medical Sciences International Ethical Guidelines
- Applicable International Council for Harmonisation (ICH) Good Clinical Practice (GCP) Guidelines
- Applicable laws and regulations

The protocol, protocol amendments, informed consent form (ICF), Investigator's Brochure (IB), and other relevant documents (eg, advertisements) must be submitted to an Institutional Review Board (IRB)/ Independent Ethics Committee (IEC) by the Investigator and reviewed and approved by the IRB/IEC before the study is initiated.

Any amendments to the protocol will require IRB/IEC approval before implementation of changes made to the study design, except for changes necessary to eliminate an immediate hazard to study subjects.

Protocols and any substantial amendments to the protocol will require health authority approval prior to initiation except for changes necessary to eliminate an immediate hazard to study subjects.

The Investigator will be responsible for the following:

- Providing written summaries of the status of the study to the IRB/IEC annually or more frequently in accordance with the requirements, policies, and procedures established by the IRB/IEC
- Notifying the IRB/IEC of SAEs or other significant safety findings as required by IRB/IEC procedures
- Providing oversight of the conduct of the study at the site and adherence to requirements of 21 Code of Federal Regulations (CFR), ICH guidelines, the IRB/IEC, European regulation 536/2014 for clinical studies (if applicable), and all other applicable local regulations

17.1.2. Financial Disclosure

Investigators and sub-Investigators will provide the Sponsor with sufficient, accurate financial information as requested to allow the Sponsor to submit complete and accurate financial certification or disclosure statements to the appropriate regulatory authorities. Investigators are responsible for providing information on financial interests during the course of the study and for 1 year after completion of the study.

17.1.3. Informed Consent Process

The Investigator or his/her representative will explain the nature of the study to the subject or their legally authorized representative and answer all questions regarding the study.

Subjects must be informed that their participation is voluntary. Subjects or their legally authorized representative (defined as the subject's parent or legal guardian) will be required to sign a statement of informed consent that meets the requirements of 21 CFR50, local regulations, ICH guidelines, Health Insurance Portability and Accountability Act (HIPAA) requirements, where applicable, and the IRB/IEC or study site.

The medical record must include a statement that written informed consent was obtained before the subject began any study-specific procedures and the date the written consent was obtained. The authorized person obtaining the informed consent must also sign the ICF.

Subjects must be re-consented to the most current version of the ICF(s) during their participation in the study.

A copy of the ICF(s) must be provided to the subject or their legally authorized representative.

A subject who is rescreened is not required to sign another ICF if the rescreening occurs within 14 days from the previous ICF signature date.

The ICF will contain a separate section that addresses the use of remaining mandatory samples for optional exploratory research. The Investigator or authorized designee will explain to each subject the objectives of the exploratory research. Subjects will be told that they are free to refuse to participate and may withdraw their consent at any time and for any reason during the storage period.

17.1.4. Data Protection

Subjects will be assigned a unique identifier by the Sponsor. Any subject records or datasets that are transferred to the Sponsor will contain the identifier only; subject names or any information which would make the subject identifiable will not be transferred.

The subject must be informed that his/her personal study-related data will be used by the Sponsor in accordance with local data protection law. The level of disclosure must also be explained to the subject who will be required to give consent for their data to be used as described in the ICF and assent form.

The subject must be informed that his/her medical records may be examined by Clinical Quality Assurance auditors or other authorized personnel appointed by the Sponsor, by appropriate IRB/IEC members, and by inspectors from regulatory authorities.

17.1.5. Dissemination of Clinical Study Data

The Sponsor will comply with current regulatory requirements for disclosure and submission of study results. The Sponsor's policy on publication of study results is described in Section 15.

17.1.6. Data Quality Assurance

All subject data relating to the study will be recorded in the electronic case report form (eCRF) unless transmitted to the Sponsor or designee electronically (eg, laboratory data). The

Investigator is responsible for verifying that data entries are accurate and correct by physically or electronically signing the case report form (CRF).

Guidance on completion of CRFs will be provided in the Completion Guidelines provided by Data Management.

The Investigator must permit study-related monitoring, audits, IRB/IEC review, and regulatory agency inspections and provide direct access to source data documents.

Quality tolerance limits (QTLs) will be predefined in the Integrated Quality Risk Management: Risk Assessment Categorization Tool to identify systematic issues that can impact subject safety and/or reliability of study results. These predefined parameters will be monitored during the study and important deviations from the QTLs and remedial actions taken will be summarized in the Clinical Study Report.

Monitoring details describing strategy (eg, risk-based initiatives in operations and quality such as Risk Management and Mitigation Strategies and Analytical Risk-Based Monitoring), methods, responsibilities, and requirements, including handling of noncompliance issues and monitoring techniques (central, remote, or on-site monitoring)are provided in the Monitoring Plan and/or the Risk Management Plan.

The Sponsor or designee is responsible for the data management of this study, including quality checking of the data.

The Sponsor assumes accountability for actions delegated to other individuals (eg, contract research organizations).

Records and documents, including signed ICFs, pertaining to the conduct of this study must be retained by the Investigator for 2 years after the last marketing application approval; or, if not approved, 2 years following the discontinuance of the investigational product for investigation.

study completion unless local regulations or institutional policies require a longer retention period. No records may be destroyed during the retention period without the written approval of the Sponsor. No records may be transferred to another location or party without written notification to the Sponsor.

17.1.7. Source Documents

Source documents provide evidence for the existence of the subject and substantiate the integrity of the data collected. Source documents are filed at the Investigator's site.

Data reported on the CRF or entered in the eCRF that are transcribed from source documents must be consistent with the source documents or the discrepancies must be explained. The Investigator may need to request previous medical records or transfer records, depending on the study. Current medical records must be available.

Definition of what constitutes source data can be found in ICH-GCP guidance for industry E6: Consolidated Guidance.

The Investigator must maintain accurate documentation (source data) that supports the information entered in the CRF.

Study monitors will perform ongoing source data verification to confirm that data entered into the eCRF by authorized site personnel are accurate, complete, and verifiable from source documents; that the safety and rights of subjects are being protected; and that the study is being conducted in accordance with the currently approved protocol and any other study agreements, ICH-GCP, and all applicable regulatory requirements.

Statistical Analysis Plan

Protocol Number: SRK-015-006

Protocol Title: A Phase 2A Randomized, Double-Blind, Placebo-Controlled, Multicenter Study to Evaluate the Efficacy, Safety, and Pharmacokinetics of Apitegromab in Overweight and Obese Adult Subjects

Study Drug: Apitegromab (SRK-015)

Protocol Version: Version 2.0

Protocol Date: 04 March 2024

Trial Name: EMBRAZE

SAP Version: 1.0

SAP Date: 14 April 2025

Author: [redacted]

Sponsor: Scholar Rock, Inc.
301 Binney Street, 3rd Floor
Cambridge, MA 02142

Notice of Confidential and Proprietary Information:

This document contains confidential information. Any use, distribution, or disclosure without the prior written consent of Scholar Rock is strictly prohibited except to the extent required under applicable laws or regulations. Persons to whom the information is disclosed must be informed that the information is confidential and may not be further disclosed by them.

REVISION HISTORY

Version #	Date	Revision Summary
1.0	14 April 2025	Original Version

APPROVAL SIGNATURE

[redacted]

TABLE OF CONTENTS

REVISION HISTORY 2
TABLE OF CONTENTS 4
LIST OF TABLES 6
LIST OF FIGURES 6
1. INTRODUCTION 9
1.1. Scope of Work 9
1.2. Timing of Analyses 9
1.3. Tables, Figures and Listing 9
2. STUDY OBJECTIVES AND ENDPOINTS 10
3. STUDY DESIGN 12
3.1. Brief Description 12
3.2. Determination of Sample Size 13
3.3. Treatment Assignment and Blinding 13
3.4. Timing of the Efficacy and Safety Analyses 13
4. ANALYSIS POPULATION 14
4.1. Completer Population 14
4.2. Intention-to-Treat Population 14
4.3. Modified Intention-to-Treat Population 14
4.4. Per-Protocol Population 14
4.5. Safety Population 14
4.6. Pharmacokinetics/Pharmacodynamics Population 14
4.7. Immunogenicity Population 15
5. GENERAL CONSIDERATIONS FOR DATA ANALYSIS AND HANDLING 16
5.1. General Consideration 16
5.1.1. Analysis Groups Definition 16
5.1.2. Key Definitions 16
5.1.3. Incomplete Date Handling 16
5.1.4. Visit Windows 16
5.2. Subject Disposition 20
5.3. Demographics and Weight Management History 20
5.4. Medical History 20

5.5. Concomitant Medication/Procedures 20
5.6. Study Drug Exposure 21
5.7. Study Drug Compliance 21
5.8. Protocol Deviations 21
6. EFFICACY ANALYSIS 22
6.1. Primary Efficacy Endpoint 22
6.1.1. Main Estimand 22
6.1.2. Supportive Estimand 22
6.1.3. Primary Analysis 22
6.1.4. Sensitivity Analysis 23
6.2. Secondary Efficacy Endpoints 23
6.3. Exploratory Efficacy Endpoints 23
6.4. Data Imputation 24
6.5. Multiplicity 24
6.6. Subgroup Analysis 24
7. SAFETY 25
7.1. Adverse Events 25
7.2. Safety Laboratory Evaluations 26
7.3. Electrocardiogram 26
7.4. Columbia Suicide Severity Rating Scale and Patient Health Qustionnaire-9 27
7.5. Physical Examination 27
7.6. Vital Signs 28
7.7. Pregnancy Test 28
8. PHARMACOKINETICS 29
9. PHARMACODYNAMICS 30
10. IMMUNOGENICITY 31
11. INTERIM ANALYSIS 32
12. CHANGES FROM ANALYSIS PLANNED IN PROTOCOL 33
13. REFERENCES 34
14. APPENDICES 35
DEXA Scan Based Efficacy Endpoints (Derivation and Definition) 35
SAS Example Code of Primary Efficacy Analysis 36

LIST OF TABLES

Table 1: Visit Mapping Schedule 18

Table 2: Categorical Criteria for Treatment-Emergent Abnormal Blood Pressure, Respiratory Rate, Body Temperature, and Heart Rate for Adults 28

LIST OF FIGURES

Figure 1: Overall Study Design 12

ABBREVIATIONS

Abbreviation	Description
A1c	glycated hemoglobin
ADA	antidrug antibody
AE	adverse event
AESI	adverse event of special interest
ATC	anatomical therapeutic chemical classification
BMI	body mass index
BP	blood pressure
CI	confidence interval
CK	creatine kinase
CRF	case report form
C-SSRS	Columbia Suicide Severity Rating Scale
CSR	clinical study report
CTCAE	Common Terminology Criteria for Adverse Events
CV	coefficient of variation
DEXA	dual-energy x-ray absorptiometry
ECG	electrocardiogram
EOI	end of infusion
EOS	End of Study
EOT	End of Treatment
ET	Early Termination
Hb	hemoglobin
HDL	high density lipoprotein
HOMA-IR	homeostatic model assessment for insulin resistance
ICE	intercurrent event
ICH	International Council for Harmonisation
ITT	intention-to-treat
IV	intravenous
IWRS	interactive web response system
LBM	lean body mass
LDL	low density lipoprotein
LST	lean soft tissue
m	number of non-zero concentrations
Max	maximum
MedDRA	Medical Dictionary for Regulatory Affairs
Min	minimum
mITT	modified intention-to-treat
n	number of observations

Abbreviation	Description
PD	pharmacodynamic(s)
PHQ-9	Patient Health Questionnaire - 9
PK	pharmacokinetic(s)
PT	preferred term
Q4W	once every 4 weeks
QTcF	QT interval corrected using Fridericia's formula
QW	weekly
SAE	serious adverse event
SAP	statistical analysis plan
SAS	Statistical Analysis System
SAT	subcutaneous adipose tissue
SD	standard deviation
SOC	system organ class
SRK-015	Scholar Rock designation for apitegromab
TAT	total adipose tissue
TEAE	treatment-emergent adverse event
TFLs	tables, figures, and listings
VAT	visceral adipose tissue
VLDL	very low density lipoprotein
WC	waist circumference
WHO-DD	World Health Organization Drug Dictionary
WHR	waist-to-hip ratio
WHtR	waist-to-height-ratio

1. INTRODUCTION

The intent of this document is to provide guidance for analyses of data for the Phase 2a randomized, double-blind, placebo-controlled, multicenter study of apitegromab in patients who are overweight or obese. This statistical analysis plan (SAP) has been developed after review of the Clinical Study Protocol Number SRK-015-006 (version 2 dated 04 March 2024).

The purpose of this SAP is to ensure that the planned data listings, summary tables, and figures and the statistical methodologies, are complete and appropriate to allow valid conclusions regarding the study objectives. This SAP was developed in accordance with International Council for Harmonisation (ICH) E9 guidance on statistical principles for clinical studies (ICH 2021). All decisions regarding the final analysis, as defined in this SAP document, will be made prior to the Database Freeze (unblinding) of the study data.

1.1. Scope of Work

This SAP covers the analyses of the Phase 2a study, SRK-015-006 (EMBRAZE), and includes all randomized subjects who enrolled in the study.

All analyses of efficacy, safety, pharmacokinetics (PK), pharmacodynamics (PD), and antidrug antibody (ADA) endpoints described in this plan will be included in the final analysis.

1.2. Timing of Analyses

No interim analysis will be conducted for this study. Once all patients complete the Treatment Period, at which time all patients will have completed study drug administration, the study team will be unblinded and the primary efficacy analysis will be performed. The Investigator and the subjects will remain blinded until the Safety Follow-up Period is completed. The details are provided in the unblinding plan.

1.3. Tables, Figures and Listing

A detailed description of the planned Tables, Figures, and Listings (TFLs) to be presented in the clinical study report (CSR) is provided in the accompanying TFLs shell document.

2. STUDY OBJECTIVES AND ENDPOINTS

**Objectives**	**Endpoints**
**Primary**	
• Evaluate the efficacy of apitegromab versus placebo when used as an adjunctive therapy to incretin mimetic therapy in subjects with overweight or obesity and without diabetes	• Change from Baseline at 24 weeks in LBM
**Secondary**	
• Evaluate the effect of apitegromab versus placebo on body weight when used as an adjunctive therapy to incretin mimetic therapy in subjects with overweight or obesity and without diabetes	• Percent change from Baseline at 24 weeks in body weight
• Evaluate the effect of apitegromab versus placebo on body composition when used as an adjunctive therapy to incretin mimetic therapy in subjects with overweight or obesity and without diabetes	• Change from Baseline at 24 weeks in DEXA measurements, including:<br>○ Percent LBM<br>○ Total and percent of fat body mass<br>○ Total and percent of VAT, SAT, and trunk fat body mass<br>○ Percent of weight loss from Baseline due to fat body mass loss or LBM
• Evaluate the PK and PD of apitegromab when used as an adjunctive therapy to incretin mimetic therapy in subjects with overweight or obesity and without diabetes	• Trough and EOI concentration of apitegromab through 24 weeks of treatment<br>• Trough concentrations of latent myostatin through 24 weeks of treatment<br>• Concentration of apitegromab and latent myostatin during Safety Follow-up Period (Week 25 through Week 40)
• Evaluate the safety and tolerability of apitegromab when used as an adjunctive therapy to incretin mimetic therapy in subjects with overweight or obesity and without diabetes	• Frequency of TEAEs and SAEs by severity<br>• Change from Baseline in clinical safety laboratory tests, vital signs, ECG measurements, and psychiatric evaluations<br>• Presence or absence of ADAs against apitegromab in serum from blood samples

Objectives	Endpoints
**Exploratory**	
• Evaluate exploratory efficacy parameters of apitegromab versus placebo when used as an adjunctive therapy to incretin mimetic therapy in subjects with overweight or obesity and without diabetes	• Change from Baseline at 32 weeks in DEXA measurements:<br>○ Proportion of subjects with categorical percent changes from Baseline in body weight, fat body mass, and/or LBM<br>• Proportion of subjects with categorical changes from Baseline in waist circumference<br>• Proportion of subjects with change from Baseline in WHtR and WHR categories<br>• Change from Baseline in force production assessed by handheld dynamometry<br>• Change from Baseline in the number of sit-to-stand repetitions in the chair sit-to-stand test
• Evaluate effect of apitegromab versus placebo on glucose metabolism, lipid metabolism, and systolic and diastolic blood pressure when used as an adjunctive therapy to incretin mimetic therapy in subjects with overweight or obesity and without diabetes	• Change from Baseline in glucose metabolism assessments, including plasma glucose, HbA1c, serum insulin, C-peptide, and HOMA-IR values<br>• Change from Baseline in lipid panel tests including total cholesterol, triglycerides, LDL, HDL, and VLDL<br>• Change from Baseline in systolic and diastolic blood pressure
• Evaluate effect of apitegromab versus placebo on an exploratory biomarker when apitegromab is used as an adjunctive therapy to incretin mimetic therapy in subjects with overweight or obesity and without diabetes	• Change from Baseline in adiponectin values
• Evaluate the PK of incretin mimetic therapy in the presence and absence of apitegromab in subjects with overweight or obesity and without diabetes	• Concentration of incretin mimetic therapy through 24 weeks of treatment

Abbreviations: A1c, glycated hemoglobin; ADA, antidrug antibody; DEXA, dual-energy x-ray absorptiometry; ECG, electrocardiogram; EOI, end of infusion; Hb, hemoglobin; HOMA-IR, homeostatic model assessment for insulin resistance; LBM, lean body mass; PD, pharmacodynamics; PK, pharmacokinetics; SAE, serious adverse event; SAT, subcutaneous adipose tissue; TEAE, treatment-emergent adverse event; VAT, visceral adipose tissue; VLDL, very low density lipoprotein; WHR, waist-to-hip ratio; WHtR, waist-to-height ratio.

3. STUDY DESIGN

3.1. Brief Description

This is a Phase 2a randomized, double-blind, placebo-controlled, multicenter study. Approximately 100 overweight or obese adult subjects without diabetes will be randomized 1:1 to receive apitegromab 10 mg/kg once every 4 weeks (Q4W) + incretin mimetic therapy weekly (QW) or placebo Q4W + incretin mimetic therapy QW. Administration of incretin mimetic therapy will be based on a dose escalation schedule, beginning at the lowest dose and escalating Q4W to the next dose until a recommended maximum tolerated maintenance dose has been reached.

As shown in Figure 1, the study will include a Screening Period (up to 4 weeks), a Treatment Period, and a Safety Follow-up Period. Subjects completing the 24-week (169-day) Treatment Period will be followed for 16 weeks (112 days) in the Safety Follow-up Period, during which time they will not be receiving any study drug (apitegromab, placebo, or incretin mimetic therapy).

Figure 1: Overall Study Design

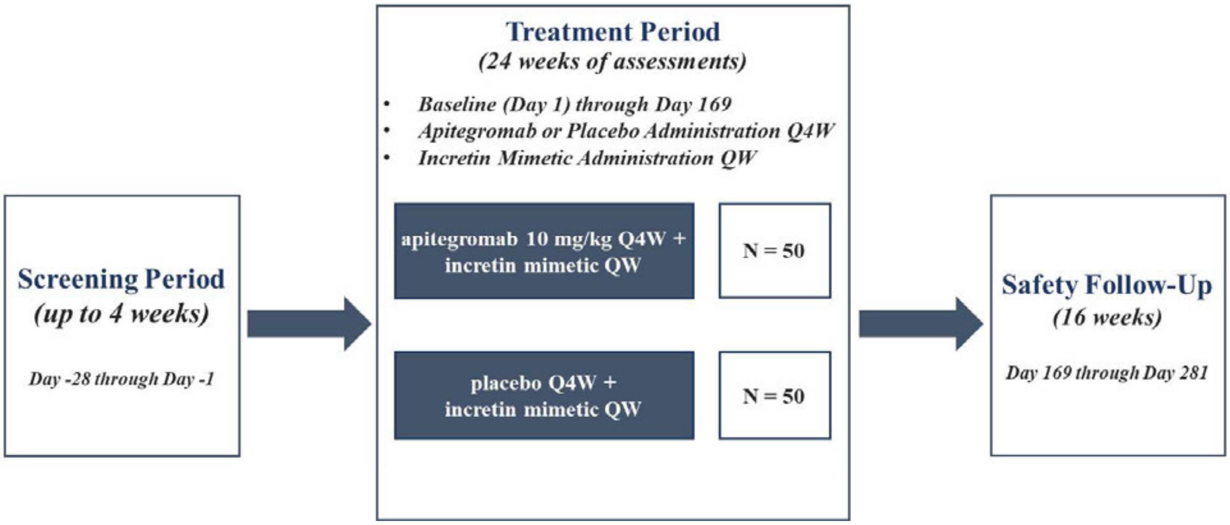

Abbreviations: Q4W, once every 4 weeks; QW, weekly.

The Schedule of Assessments for the study is provided in the study protocol. During the Screening Period, all subject Screening activities and eligibility determinations will be conducted after written informed consent has been provided and within 28 days before administration of the first dose of study drug. Assessments conducted during the study will include cardiometabolic function measurements, body weight and composition changes, assessments of a circulating biomarker and fasting glucose metabolism, PK and PD measurements, testing for (ADAs, and safety monitoring. Dual-energy X-ray absorptiometry (DEXA), a 3 compartmental model for measuring bone mineral, fat mass, and lean soft tissue (LST) (Prado 2014), will be used to evaluate body composition. Data will be reviewed on an ongoing basis in a blinded manner by the Medical Monitor(s) and the Sponsor to ensure subject safety and study integrity.

All subjects will receive the first administration of incretin mimetic therapy at the study site on Visit 1 (Day 1), at which time they will also be trained to self-administer incretin mimetic therapy QW, using the injection pen, and will receive a 1-month supply of incretin mimetic therapy. Thereafter, all subjects will receive additional 1-month supplies of incretin mimetic therapy at the Q4W visits (Visit 2 [Day 29], Visit 3 [Day 57], Visit 4 [Day 85], Visit 5 [Day 113], and Visit 6 [Day 141]).

Subjects will be randomized 1:1 in a double-blind manner to receive either apitegromab or placebo via intravenous (IV) infusion at Visits 1 through 6 at the study sites, with subject visits occurring approximately every 4 weeks through the end of the Treatment Period.

Upon completion of the Treatment Period, all subjects will enter the Safety Follow-up Period where they will be monitored for safety. During this time, subjects will not be receiving any study drug (apitegromab, placebo, or incretin mimetic therapy).

3.2. Determination of Sample Size

The estimated difference between the apitegromab + incretin mimetic therapy group and the placebo + incretin mimetic therapy group in mean change from Baseline of LBM at Week 24 was used to calculate the sample size. Assuming the change follows an approximately normal distribution with a standard deviation (SD) of 4.6 kg, a sample size of 50 subjects per arm yields approximately 80% power to detect an effect size of 2 kg at an overall 2-sided 20% significance level. If approximately 13% of subjects are not evaluable for an analysis, a sample size of 43 subjects per arm yields approximately 75% power to detect an effect size of 2 kg.

3.3. Treatment Assignment and Blinding

Subjects are randomized to receive apitegromab 10 mg/kg Q4W + incretin mimetic therapy QW or placebo Q4W + incretin mimetic therapy QW. Administration of the incretin mimetic therapy is based on a dose escalation schedule, beginning at the lowest dose and escalating Q4W to the next dose until a maximum tolerated maintenance dose has been reached.

This is a double-blind, placebo-controlled study. The Sponsor, subjects, Investigators, and site personnel, with the exception of the Pharmacist, are blinded to treatment assignments.

The Sponsor study team remains blinded until the end of the Treatment Period, when the unblinded efficacy analysis is performed. The Investigators, site personnel, and all subjects remain blinded until the Safety Follow-up Period is completed. The details of the blinding and unblinding process are specified in the unblinding plan.

3.4. Timing of the Efficacy and Safety Analyses

At the end of the Treatment Period, the primary and secondary efficacy endpoints, along with the safety endpoints collected up to that point will be analyzed with unblinded treatment assignment. Afterwards, at the end of the Safety Follow-up Period, the applicable exploratory efficacy endpoints and all safety endpoints collected throughout the study will be analyzed. The results will be used for the final CSR.

4. ANALYSIS POPULATION

4.1. Completer Population

The completer population is defined as all randomized subjects who completed the Treatment Period and have both evaluable baseline and postbaseline DEXA measurement of LBM at the scheduled Visit 7. The completer population will be used for primary efficacy analysis. Subjects will be analyzed according to the treatment group to which they were randomized.

4.2. Intention-to-Treat Population

The intention-to-treat (ITT) population is defined as all randomized subjects, including the subjects who did not receive the correct treatment, or the subjects who did not follow the protocol. Subjects will be analyzed according to the treatment group to which they were randomized. All efficacy analyses, along with summaries of demographics and weight management history, subject disposition, medical history, and prior and concomitant medications will be based on the ITT population.

4.3. Modified Intention-to-Treat Population

The modified ITT (mITT) population is defined as all randomized subjects who have evaluable baseline and one postbaseline DEXA measurement of lean body mass (LBM). The mITT population will be used for efficacy analysis. Subjects will be analyzed according to the treatment group to which they were randomized.

4.4. Per-Protocol Population

The per-protocol population is defined as the completer population excluding any subjects with important protocol deviations deemed to impact efficacy analyses or any subject that did not meet inclusion/exclusion criteria. The per-protocol population will be used for efficacy analysis. Subjects will be analyzed according to the treatment group to which they were randomized. If per-protocol population is identical with any of the 3 analysis populations above, this population will not be used for analyses.

4.5. Safety Population

The safety population includes all randomized subjects who received at least 1 dose of study drug. The analyses based on the Safety population will be summarized based on the actual treatment the subjects received.

4.6. Pharmacokinetics/Pharmacodynamics Population

Assessment of PK and PD of study drug apitegromab, and PK of incretin mimetic therapy will be performed in this study. The PK/PD population is defined as all subjects who receive at least 1 dose of study drug, or incretin mimetic therapy and have at least 1 quantifiable PK/PD result. The PK/PD population will be used for PK/PD analysis. The analyses based on the PK/PD population will be summarized based on the actual treatment the subjects received.

4.7. Immunogenicity Population

Assessment of immunogenicity data will be performed in this study. The immunogenicity population is defined as all subjects who receive at least 1 dose of study drug or incretin mimetic therapy and have at least 1 quantifiable ADA result. The immunogenicity population will be used for ADA analysis. The analyses based on the immunogenicity population will be summarized based on the actual treatment the subjects received.

5. GENERAL CONSIDERATIONS FOR DATA ANALYSIS AND HANDLING

5.1. General Consideration

Summary statistics and statistical analyses will only be presented for the data detailed in this SAP. Relevant data will be included in listings and presented by treatment group, by subject, and by visit where applicable.

The statistical analyses for efficacy endpoints will be conducted using the method detailed in Section 6. No formal hypothesis testing will be performed. Any p-values displayed should be considered descriptive, and no adjustment for multiplicity will be performed. For all applicable estimates, the 2-sided 80% confidence internal (CI) will be reported.

For descriptive summaries, continuous variables will be summarized using the number of observations (n), mean, SD, median, minimum, and maximum. Categorical variables will be summarized using number of observations (n), frequency, and percentage. The applicable endpoints will be analyzed using the survival analysis method detailed in Section 6.

All analyses will be conducted using Statistical Analysis System (SAS® Version 9.4 or later, Cary, North Carolina).

5.1.1. Analysis Groups Definition

In general, efficacy and safety data will be presented by treatment group along with overall in total.

5.1.2. Key Definitions

Study Day 1 is defined as the day when the first dose of study drug is taken, as recorded on the Study Drug Administration Case Report From (CRF) page.

Study Days are calculated relative to Study Day 1. For an event that occurred after Study Day 1, the study day is calculated as (visit/event date minus Study Day 1 plus 1). For an event that occurred before Study Day 1, the study day is calculated as (visit/event date minus Study Day 1).

Baseline for all efficacy and safety variables is defined as the last non-missing measurement prior to the first dose of study drug.

5.1.3. Incomplete Date Handling

An adverse event (AE) with partial start/end dates will be treated as treatment-emergent unless the partial information excludes the possibility (eg, the partial end date indicates the AE ended before the first dosing of the study drug).

5.1.4. Visit Windows

In general, data will be summarized or analyzed by visit for summary statistics and statistical analyses according to the scheduled visit as outlined in the protocol.

Early Termination (ET) Visit may be mapped into a scheduled visit using the window scheme shown in Table 1. Similarly, if records on a scheduled visit are not available, records collected

on unscheduled visit can be used if it fits into the visit window. If there are 2 or more ET and unscheduled assessments available in the same analysis window for a subject, the one that is closest to the target visit day will be used for analysis. If there are 2 or more ET or unscheduled assessments in the same analysis window with the same distance from the target visit day, use the ET visit over unscheduled visit. If both visits are unscheduled, the earlier/earliest assessment will be used.

Table 1: Visit Mapping Schedule

Activity/Assessment	SCR	Treatment Period							Safety Follow-up Period	
**Visit<br>Timepoint (Study Day)**	**SCR<br>-28 to -1**	**V1<br>1**	**V2<br>29**	**V3<br>57**	**V4<br>85**	**V5<br>113**	**V6<br>141**	**V7<br>169<br>EOT/ET**	**V8<br>Day 84 after last dose**	**V9<br>Day 140 after last dose**
**Visit window (study days)**	**<=-1**	**1**	**2-36**	**37-64**	**65-92**	**93-120**	**121-148**	**149-(176 or EOT/ET)**	**>EOT/ET-112 post last dose**	**>112 post last dose**
Body weight	X	X	X	X	X	X	X	X	X	X
BMI	X	X	X	X	X	X	X	X	X	X
DEXA scan		X						X (Day 2-EOT/ET)	X	X
Handheld dynamometry<br>Chair sit-to-stand test		X						X (Day 2-EOT/ET)	X	X
12-lead ECG		X						X (Day 2-EOT/ET)		X<br>(>EOT/ET)
Waist and hip circumference<br>C-peptide<br>Serum insulin<br>Adiponectin		X			X<br>(Day 2-127)			X (Day 128-EOT/ET)	X	X
Lipid Panel<br>HbA1c	X	X			X<br>(Day 2-127)			X (Day 128-EOT/ET)	X	X
Physical examination	X	X	X	X	X	X	X	X	X	X
Vital signs	X	X	X	X	X	X	X	X	X	X
Safety laboratory tests	X	X	X	X	X	X	X	X	X	X
PK and PD sampling		X	X	X	X	X	X	X	X	X
ADA sampling		X	X	X	X	X	X	X	X	X

Abbreviations: EOT, End of Treatment; ET, Early Termination; EOS, end of study; V, Visit. ADA, antidrug antibody; BMI, body mass index; PD, pharmacodynamics; PK, pharmacokinetics.

Note: Other than the assessment visits with window days specified in brackets, the study visit window follows the study day specified in the row of “Visit Window (study days).” Subjects who ET prior to V7 and agree to participate in the Safety Follow-up Period visits will complete V7/EOT/ET as their ET visit and then complete V8 and V9 during the Safety Follow-up Period. Subjects who ET prior to V7 and do not agree to participate in the Safety Follow-up Period visits will complete V9/EOS/ET as the ET visit. The ET Visit should be conducted within 8 weeks of the last apitegromab or placebo dose.

5.2. Subject Disposition

The summary of subject disposition will be provided by treatment groups and overall using the ITT population. This summary will include the number of subjects randomized. The summary will include the number and percentage for the following categories where the percentage is based on the number of randomized subjects:

- Subjects dosed
- Treatment Period completion status and primary reason for Treatment Period discontinuation
- Study completion status and primary reason for study discontinuation

The listing of subject disposition and inclusion of each population at subject level will be provided.

5.3. Demographics and Weight Management History

Descriptive summaries of demographic (age, sex, race, and ethnicity) and weight management history will be summarized by treatment group along with overall in total using the ITT population.

The listings of subject demographic data and weight management history at subject level will be provided.

5.4. Medical History

Medical history information will be coded using Medical Dictionary for Regulatory Activities (MedDRA) version 27.0 or higher and summarized by treatment group using the ITT population. Summaries will be ordered alphabetically by system organ class (SOC) and then, within a SOC, alphabetically by preferred term (PT). A subject will be counted once for each SOC, and counted once for each PT within SOC. If there are any subjects who received study drug different from what was assigned, the summary table will also be repeated using the Safety population.

The listing of medical history data at subject level will be provided.

5.5. Concomitant Medication/Procedures

Concomitant medications will be coded using the World Health Organization Drug Dictionary (WHO-DD), version March 2024 or above. Medications initiated prior to the start of treatment and maintained during the study, or taken during the course of the study will be considered as concomitant medications. Medications received prior to and not continuing during the study treatment will be considered as prior medications. Medications with partial or missing start date will be assumed to be concomitant medications, unless there is clear evidence (through comparison of partial dates or end date with the first dose date of study drug) to suggest that the medications are not taken during treatment. Concomitant medications will be summarized by anatomical therapeutic chemical classification (ATC) main class (1st level) and preferred name. Prior medications will be presented in the corresponding listing.

Concomitant interventions or procedures will be presented in a listing.

5.6. Study Drug Exposure

The apitegromab or placebo dose regimen will be 10 mg/kg Q4W. The dose will be administered by IV infusion.

The study drug of apitegromab or placebo received during Treatment Period will be summarized with the parameters below:

- Exposure duration in weeks (Treatment Period = (last dose date-date of first dose+1)/7)
- Total number of infusions

The infusion interruption/start status, infusion not received or infusion interruption reason, as well as overdose status will be presented in the listing.

The incretin mimetic therapy subjects received will be summarized separately by treatment group as well:

- Number and percentages of subjects who received the incretin mimetic therapy
- Number of doses administered
- Counts of doses administered at incretin mimetic therapy titration levels (2.5mg, 5mg, 7.5mg, 10mg, 12.5mg, and 15mg) and at the highest level

The incretin memetic therapy administration status and missed reason will be presented in the listing.

5.7. Study Drug Compliance

Study drug compliance in percentage will be calculated as follows:

$$100 \times \frac{\text{Number of infusions received}}{\text{Number of infusions expected to be received}}$$

The number of infusions expected to be received is based on the last Treatment Period visit where the subject received treatment (eg, if V4 was the last visit where they were dosed then # expected = 4).

Subject counts and percentages will be summarized for compliance categories (ie, ≥80% versus <80%).

5.8. Protocol Deviations

The listing of important and nonimportant protocol deviations will be provided. The study team will review the comprehensive list of protocol deviations and identify the important protocol deviations that could have significant impact on the efficacy analysis before database lock. Subjects with such protocol deviations would be excluded from per-protocol population for efficacy analysis and the identified list will be reported.

6. EFFICACY ANALYSIS

6.1. Primary Efficacy Endpoint

6.1.1. Main Estimand

The primary efficacy estimand (ICH E9(R1) Addendum 2019) for the primary efficacy endpoint "Change from Baseline at 24 weeks in LBM" is the main estimand for the comparison between apitegromab and placebo.

- Treatment: Apitegromab 10 mg/kg Q4W + incretin mimetic therapy QW or placebo Q4W + incretin mimetic therapy QW during the 24-week Treatment Period.
- Population: Completer population
- Variable: Change from Baseline at 24 weeks in LBM
- Population-level Summary: The mean difference between change from baseline at 24 weeks in LBM
- Intercurrent events (ICEs) and Strategies for Handling ICEs:
    - Premature stopping of study treatment due to AE: not applicable.
    - Premature stopping of the study treatment due to death: not applicable.
    - Premature stopping of study treatment due to reasons other than AE and death: not applicable.
    - Modification of incretin mimetic therapy: Treatment policy strategy will be applied where values after the ICEs will be used.

6.1.2. Supportive Estimand

The supportive estimand for the primary efficacy analysis will be based on efficacy analysis populations other than the completer population, with all other attributes of the main estimand maintained.

6.1.3. Primary Analysis

Let μ_p and μ_a stand for the average change from Baseline in LBM (measured in kg) at Week 24 for subjects in the placebo + incretin mimetic therapy group and the apitegromab + incretin mimetic therapy group, respectively. The null hypothesis of the primary analysis is $\mu_p \geq \mu_a$ against the alternative hypothesis $\mu_p < \mu_a$. As this is a proof-of-concept trial, the hypothesis testing is not formal.

The difference between treatment groups in change from Baseline in LBM at Week 24 will be estimated with a 2-sided 80% CI and corresponding p-value using a linear regression model, controlling for Baseline body weight, Baseline LBM, age, and sex. Restricted maximum likelihood will be used as the estimation method.

6.1.4. Sensitivity Analysis

Other than baseline body weight, baseline LBM, age, and sex, other covariates may be included in the linear regression model as sensitivity analysis in an ad-hoc way as part of the exploratory analyses. Confidence level and p-values will be calculated using the same method at 2-sided 80% CI level as the primary analysis unless otherwise noted.

6.2. Secondary Efficacy Endpoints

The secondary efficacy endpoints include:

- Percent Change from Baseline at 24 weeks in body weight
- Change from Baseline at 24 weeks in DEXA measurements, including:
    - Percent LBM
    - Total and percent of fat body mass
    - Total and percent of VAT, SAT, and trunk fat body mass
    - Percent of weight loss from baseline due to fat body mass loss or LBM

The difference between treatment groups in the secondary efficacy endpoints above will be analyzed using a linear regression model, controlling for baseline body weight, baseline LBM, age, and sex. Restricted maximum likelihood will be used as the estimation method. The 2-sided 80% CIs will be reported. The nominal p-values of the difference between treatment groups in the secondary efficacy endpoints might be calculated but no multiplicity adjustment will be made.

6.3. Exploratory Efficacy Endpoints

The exploratory efficacy endpoints include:

- Change from Baseline at 32 weeks in DEXA measurements:
    - Proportion of subjects with categorical percent changes from Baseline in body weight (weight reduction ≥5%, 10%, 15%, and 20%), fat body mass, and/or LBM
- Proportion of subjects with categorical changes from Baseline in waist circumference (>5 cm loss, >2.5 to ≤5 cm loss, ≤2.5 cm loss or gain, >2.5 to ≤5 cm gain, and >5 cm gain)
- Proportion of subjects with shift status from Baseline categories in WHtR (<0.55 versus ≥0.55) and WHR categories (<0.85 versus ≥0.85 for women; <1 versus ≥1 for men)
- Change from Baseline in force production assessed by handheld dynamometry
- Change from Baseline in the number of sit-to-stand repetitions in the chair sit-to-stand test
- Change from Baseline in glucose metabolism assessments, including plasma glucose, HbA1c, serum insulin, C-peptide, and HOMA-IR values. HOMA-IR value is derived using the formula: fasting glucose (mg/dL) × fasting insulin (μU/mL)/405.
- Change from Baseline in lipid panel tests including total cholesterol, triglycerides, LDL, HDL, and VLDL

- Change from Baseline in systolic and diastolic blood pressure
- Change from Baseline in adiponectin values

The maximum value of the three measurements of each handheld dynamometry will be used for analysis. The analysis of handheld dynamometry will be provided by sex, left hand, right hand, and dominant hand respectively.

The difference between treatment groups in the numeric change from baseline exploratory efficacy endpoints above will be analyzed using a linear regression model, controlling for baseline body weight, baseline LBM, age, and sex. Restricted maximum likelihood will be used as the estimation method. The 2-sided 80% CIs and nominal p-values will be reported. .

The categorical endpoints above will be summarized with number of subjects, frequency and percentages by treatment group, or status shift tables as applicable.

Other exploratory endpoints may be derived and analyzed as needed.

In addition to change from baseline, change from baseline in percentage may be explored for any efficacy endpoints.

Correlation between efficacy endpoints may be explored.

6.4. Data Imputation

In efficacy analysis with missing data for LBM at Week 24, the LBM value will be imputed as the body weight measurement closest to Day 169 (ie, the target day for the Week 24 Visit) multiplied by the subject's Baseline LBM in percentage. The LBM percent is computed as the percentage of total LBM out of the total mass (TM). If baseline LBM is missing, the average baseline LBM in the treatment group may be used as the imputed value. Alternative imputation methods may be employed, as well, as part of the sensitivity analyses.

If a subject terminates the study treatment early, the first DEXA scan after the last dose will be included in the treatment period for efficacy analysis.

6.5. Multiplicity

For this proof-of-concept study, any hypothesis testing is considered exploratory. No adjustment for multiple comparisons will be applied.

6.6. Subgroup Analysis

Subgroup analyses by various factors (selected baseline characteristics) may be performed as applicable to explore whether the treatment effect and/or safety profile of study drug is similar across subgroups.

7. SAFETY

Safety analyses will be based on the safety population. Safety will be assessed based on AEs, clinical laboratory data, electrocardiogram (ECG) parameters, psychiatric instruments, physical examinations, vital signs, and pregnancy tests.

7.1. Adverse Events

All AEs will be analyzed based upon the principle of treatment emergence. Treatment-emergent adverse events (TEAEs), including SAEs (ie, serious TEAEs) will be summarized in the safety analyses unless otherwise specified. A TEAE is defined as an AE that started or worsened in severity on or after the date of first dose of study drug.

For summaries of TEAEs by SOC and PT, a subject will be counted once at the SOC level and once at each PT within the SOC level. For summaries of TEAE by SOC, PT, and maximum Common Terminology Criteria for Adverse Events (CTCAE) grade, a subject will be counted once at the highest CTCAE grade level for which the event occurred at the SOC level and the highest CTCAE grade level for each unique PT within that SOC level. In cases when CTCAE is missing for a TEAE, a Grade 3 will be imputed. In cases when relationship to study treatment is missing for a TEAE, it will be considered as related to study treatment.

The summaries presenting frequency of TEAEs by SOC and PT will be ordered by overall descending frequency of SOC and then, within an SOC, by overall descending frequency of PT.

An overall summary table will be provided which includes the number of TEAEs, the number and percentage of subjects will be presented for TEAEs, study drug-related TEAEs, serious TEAEs, study drug-related serious TEAEs, TEAE leading to drug withdrawn, TEAE leading to drug interruption, TEAE leading to dose modification, and TEAEs leading to death, AESIs (pancreatitis, liver injury, depression/suicidality, creatine kinase (CK) elevations, and other), incretion memetic therapy related TEAE, incretion memetic therapy related serious TEAE, and procedure related TEAE.

The following AE tables will be provided by treatment group:

- Summary of TEAEs by SOC and PT
- Summary of TEAEs by SOC and PT with at least 5% PT
- Summary of TEAEs by SOC and PT, and maximum severity
- Summary of TEAEs by PT
- Summary of Study Drug-related TEAEs by SOC and PT
- Summary of Study Drug-related TEAEs by SOC and PT, and maximum severity
- Summary of Serious TEAEs by SOC and PT
- Summary of Serious TEAEs by SOC and PT, and maximum severity
- Summary of Study Drug-related Serious TEAEs by SOC and PT
- Summary of Study Drug-related Serious TEAEs by SOC and PT, and maximum severity

- TEAEs leading to drug withdrawn by SOC and PT
- TEAEs of subjects who withdraw from the study due to AE
- TEAEs leading to death by SOC and PT
- TEAE with Severity Grade ≥3 by PT

The following AE listings will be provided which include those that are not TEAEs:

- All AEs
- SAEs
- Treatment-related SAEs
- AEs leading to drug withdrawal
- All deaths
- AESIs (pancreatitis, liver injury, depression/suicidality, CK elevations)
- Non-treatment Emergent AEs

7.2. Safety Laboratory Evaluations

The safety laboratory tests are specified in Table 10 and Table 11 of the protocol in Section 9.1.5. All hematology, serum chemistry, thyroid, urinalysis, and coagulation results will be converted into SI units (the International System of Units) for summary. All data will be listed per subject for each assessment. Laboratory determinations categorized as in or out of normal range (low or high) will be indicated in the listings. For laboratory tests with continuous data, descriptive statistics will be tabulated based on actual value and change from baseline value by visit, as well as minimum and maximum values across the postbaseline values. Selected laboratory tests may be plotted by visit using box and whisker plots as applicable.

Laboratory values will be graded and summarized based on applicable normality categories. The number and percentage of subjects with worst grade across all postbaseline values will be tabulated by test. Shift analyses will be provided for laboratory tests at Baseline contrasted with worst postbaseline grade with subject count and percentage.

7.3. Electrocardiogram

Triplicate 12-lead electrocardiograms (ECGs) are to be performed with the subject in the supine position after the subject rests for at least 5 minutes before the first reading, and then remains resting during the subsequent readings. The 3 individual ECG tracings should be obtained as closely as possible in succession, but no more than 2 minutes apart. The ECGs will be obtained using an ECG machine that automatically calculates the heart rate and measures PR, QRS, QT, RR, and QT interval corrected using Fridericia’s formula (QTcF) intervals. The average of all non-missing 12-lead electrocardiogram (ECG) triplicate measurements will be calculated and summarized with descriptive statistics for each treatment group by visit. The baseline value and change from baseline at each visit will be summarized by treatment group as well. The subject count and percentage of change from overall interpretation of normal at baseline to abnormal at any of the postbaseline visit will be summarized by actual treatment group.

The counts and percentages of subjects who meet the following criteria at any time during the double-blind Treatment Period, through the Safety Follow-up Period will be summarized by treatment group:

- QTcF >450 msec
- QTcF >480 msec
- QTcF >500 msec
- QTcF increase from baseline >30 msec
- QTcF increase from baseline >60 msec
- QTcF >500 msec at any time post-baseline and >60 msec increase from baseline

Shift tables will be used to produce the changes in ECG values from baseline to each postbaseline visit where ECG is performed. Shift tables will include counts and percentages of subjects who shift from normal ECG at baseline to abnormal ECG at any postbaseline visit where ECG is performed.

7.4. Columbia Suicide Severity Rating Scale and Patient Health Qustionnaire-9

The Columbia Suicide Severity Rating Scale (C-SSRS) and the Patient Health Questionnaire-9 (PHQ-9) are both validated psychiatric instruments. They are utilized to assess baseline and treatment-emergent suicidal ideation/behavior and depressive symptoms, respectively, in subjects at visits specified in the protocol.

The number of subjects who completed the C-SSRS and the number and percentage of subjects with positive suicidal ideation (responses of "yes" to one or more screening questions) and suicidal behavior will be summarized by treatment group and by visit. The treatment-emergent suicidal ideation and suicidal behavior will be summarized by treatment group. In addition, the severity of suicidal ideation and the corresponding change from baseline will be summarized by treatment group and by visit. The relevant listing of C-SSRS score and each domain at the subject level will be provided.

The PHQ-9 is a validated self-administered screening tool that assesses the presence and intensity of depressive symptoms. The PHQ-9, which incorporates the 9 Diagnostic and Statistical Manual IV depression criteria as "0" (not at all) to "3" (nearly every day). During the study conduct, a subject will be referred to a mental health professional for a PHQ-9 score of >10, any suicidal behavior, or any suicidal ideation of type 4 or 5 on the C-SSRS.

The number of subjects who completed the PHQ-9 and the number and percentage of subjects with PHQ-9 total score >10 (signifying moderate depression) will be summarized by treatment group and by visit. The relevant listing of PHQ-9 score and each questionnaire result at the subject level will be provided.

7.5. Physical Examination

A listing of all physical examination assessments will be presented for the Safety Population.

7.6. Vital Signs

Vital sign assessments will include heart rate, blood pressure, body temperature, and respiratory rate (Table 2). The assessments will be summarized with descriptive statistics for each treatment group by visit. The baseline value and change from baseline at each visit will be summarized by treatment group as well.

The counts and percentages of subjects with treatment-emergent abnormal (high or low) vital signs (sitting systolic blood pressure, diastolic blood pressure, respiratory rate, body temperature, and heart rate) will be summarized by treatment group and overall.

Table 2: Categorical Criteria for Treatment-Emergent Abnormal Blood Pressure, Respiratory Rate, Body Temperature, and Heart Rate for Adults

Parameter	Low	High
Systolic BP (mmHg)	≤90 and decrease from baseline≥20	≥160 and increase from baseline ≥20
Diastolic BP (mmHg)	≤50 and decrease from baseline ≥10	≥100 and increase from baseline ≥10
Respiratory Rate (breaths/min)	≤8	≥20
Body Temperature	<35.4	>37.8
Heart Rate (bpm)	<45	>100

Abbreviations: BP, blood pressure; bpm, beats per minute.

7.7. Pregnancy Test

By subject listing will be provided for pregnancy test and any other applicable tests.

8. PHARMACOKINETICS

Assessment of PK and PD of study drug apitegromab, and PK of incretin mimetic therapy will be performed in this study. All subjects who receive at least 1 dose of study drug or incretin mimetic therapy and have at least 1 quantifiable PK result will be included in the PK analysis. The apitegromab concentrations from this study may be combined with other study data to support the population PK analyses.

Descriptive statistics of PK parameters will include number of subjects (N), observed number of subjects (n), number of non-zero concentrations (m), mean, SD, coefficient of variation (CV)%, minimum (min), and maximum (max). A geometric mean and geometric CV% will also be presented for plasma PK parameters, if appropriate.

Graphical presentations of primary PK data may be presented (geometric mean and individual values).

9. PHARMACODYNAMICS

All subjects who receive at least 1 dose of study drug and have at least 1 quantifiable PD result will be included in the PD analysis. Apitegromab concentrations and circulating latent myostatin concentrations will be summarized by visit. The apitegromab and latent myostatin concentrations from this study may be combined with other study data to support the PK/PD analyses.

10. IMMUNOGENICITY

All subjects who receive at least 1 dose of study drug and have at least 1 ADA result will be included in the immunogenicity evaluation. The number and percentage of subjects who become positive for ADAs and who develop neutralizing antibodies will be summarized by Baseline ADA status (negative, positive, or missing). Antibody results for subjects with any positive value will be listed.

11. INTERIM ANALYSIS

No interim analysis will be conducted for this study.

12. CHANGES FROM ANALYSIS PLANNED IN PROTOCOL

Not applicable.

13. REFERENCES

ICH E9 (R1): Statistical Principles for Clinical Trials: Addendum: Estimands and Sensitivity Analysis in Clinical Trials Guideline for Industry. 2021

Prado CMM, Heymsfield SB. Lean tissue imaging: a new era for nutritional assessment and intervention. JPEN J Parenter Enteral Nutr. 2014;38: 940-953.

14. APPENDICES

DEXA Scan Based Efficacy Endpoints (Derivation and Definition)

- LBM, total mass, body fat mass, percent of body fat mass, total VAT are directly measured in DEXA scan results.
- LBM percentage is calculated based on LBM and total mass, using formula LBM (%)=(LBM/TM)*100%.
- Percent VAT is calculated based on VAT and total body fat, using formula VAT (%)=(VAT/TAT)*100%
- SAT (%)=((TAT-VAT)/TAT)*100%. TAT and VAT are directly measured in DEXA scan results.

SAS Example Code of Primary Efficacy Analysis

```
proc mixed data=Adam.ADEFF order=data method=reml;
  class trt01p sex;
  model CHG= trt01p base weight age sex/solution ;
  lsmeans trt01p / pdiff tdiff CL alpha=0.2;
  estimate 'SRK-015/Placebo' trt01p 1 -1;
run;
```

Note: TRT01P=planned treatment group; Base=baseline LBM; CHG=change form baseline in LBM (kg) at Week 24. Baseline weight, age, and sex are baseline covariates controlled in the model.
