# Supplementary material for: Apitegromab for lean mass preservation during tirzepatide-induced weight loss: a randomized, double-blind, placebo-controlled phase 2 trial
Source: Nat Med. 2026 Jun 8;32(7):2673–8. doi: 10.1038/s41591-026-04440-4 (PMC13375528; doi:10.1038/s41591-026-04440-4)
Supplement: Supplementary file 2 — Reporting Summary [file 41591_2026_4440_MOESM2_ESM.pdf]

nature portfolio

Corresponding author(s): Richard E. Pratley
AdventHealth Translational Research Institute
301 E Princeton St
Orlando, FL 32804
407-303-2519
Richard.Pratley.MD@AdventHealth.com

Last updated by author(s): Apr 1, 2026

Reporting Summary

Nature Portfolio wishes to improve the reproducibility of the work that we publish. This form provides structure for consistency and transparency in reporting. For further information on Nature Portfolio policies, see our Editorial Policies and the Editorial Policy Checklist.

Statistics

For all statistical analyses, confirm that the following items are present in the figure legend, table legend, main text, or Methods section.

n/a	Confirmed	
☐	☒	The exact sample size (*n*) for each experimental group/condition, given as a discrete number and unit of measurement
☐	☒	A statement on whether measurements were taken from distinct samples or whether the same sample was measured repeatedly
☐	☒	The statistical test(s) used AND whether they are one- or two-sided<br>*Only common tests should be described solely by name; describe more complex techniques in the Methods section.*
☐	☒	A description of all covariates tested
☐	☒	A description of any assumptions or corrections, such as tests of normality and adjustment for multiple comparisons
☐	☒	A full description of the statistical parameters including central tendency (e.g. means) or other basic estimates (e.g. regression coefficient) AND variation (e.g. standard deviation) or associated estimates of uncertainty (e.g. confidence intervals)
☐	☒	For null hypothesis testing, the test statistic (e.g. *F*, *t*, *r*) with confidence intervals, effect sizes, degrees of freedom and *P* value noted<br>*Give P values as exact values whenever suitable.*
☒	☐	For Bayesian analysis, information on the choice of priors and Markov chain Monte Carlo settings
☒	☐	For hierarchical and complex designs, identification of the appropriate level for tests and full reporting of outcomes
☒	☐	Estimates of effect sizes (e.g. Cohen's *d*, Pearson's *r*), indicating how they were calculated

Our web collection on statistics for biologists contains articles on many of the points above.

Software and code

Policy information about availability of computer code

Data collection	Summarization of data was done using Statistical Analysis System (Version 9.4 or later, Cary, North Carolina).
Data analysis	All analyses were conducted using Statistical Analysis System (Version 9.4 or later, Cary, North Carolina).

For manuscripts utilizing custom algorithms or software that are central to the research but not yet described in published literature, software must be made available to editors and reviewers. We strongly encourage code deposition in a community repository (e.g. GitHub). See the Nature Portfolio guidelines for submitting code & software for further information.

Data

Policy information about availability of data

All manuscripts must include a data availability statement. This statement should provide the following information, where applicable:

- Accession codes, unique identifiers, or web links for publicly available datasets
- A description of any restrictions on data availability
- For clinical datasets or third party data, please ensure that the statement adheres to our policy

Scholar Rock, Inc. is committed to sharing deidentified clinical trial data with external investigators upon reasonable request. Individual researchers requesting clinical trial data for academic or non-commercial use must reach out to Scholar Rock, Inc. (medicalinformation@scholarrock.com) and include a research proposal

clarifying how the data will be used, including proposed analysis methodology. Inquiring researchers should anticipate a response acknowledging their request within two to three business days. Scholar Rock, Inc. will consider and evaluate unsolicited requests for clinical trial data on a case-by-case basis.

Research involving human participants, their data, or biological material

Policy information about studies with human participants or human data. See also policy information about sex, gender (identity/presentation), and sexual orientation and race, ethnicity and racism.

Reporting on sex and gender	Male and female participants who met the inclusion criteria and were at least 18 years old, were eligible to enroll in the study. All participants provided written informed consent prior to participating in any study-specific procedures. Sex at birth was self-reported by each eligible participant and is presented in the demographic table within the manuscript (Table 1). Study outcomes were expected to be generally consistent across sex, which was shown in the exploratory post-hoc subgroup analysis of the primary end point by sex (Extended Data Table 3).
Reporting on race, ethnicity, or other socially relevant groupings	The race of enrolled patients was reported in the demographics table as White, Black or African American, and 'Other' (Table 1). The 'Other' race category included American Indian or Alaska Native, Asian, multiple races or other (unspecified). Race was self-reported by the participants. Patient consent to report these data was obtained. Individual-level data was not reported for this study.
Population characteristics	Participant characteristics are provided in Table 1 of the manuscript, including age, sex, race, baseline weight, lean body mass, BMI, body fat mass, HbA1C levels, glucose levels, and cholesterol levels. Age, sex, weight, and lean body mass were characteristics used as covariates in these analyses.
Recruitment	Patient enrollment/recruitment began on June 18, 2024 and ended on September 17, 2024. Patient selection was conducted by the Principal Investigators in accordance with each institution's established procedures and the requirements outlined in the approved study protocol. Following review, patient information was submitted to the Medical Monitor for confirmation of eligibility. This standardized recruitment process minimizes the potential for self-selection and other confounding biases. Eligibility criteria are described in the manuscript.<br><br>Participant data was collected in health clinic settings at 7 sites across the United States. Data collection took place from May 21, 2024 (first screening visit) to June 23, 2025 (final study visit).
Ethics oversight	The trial protocol was approved by Advarra, a central institutional review board as well as the institutional review boards at participating sites: ProSciento CRU (Chula Vista, California, United States, 91911), AdventHealth Translational Research Institute (Orlando, Florida, United States, 32804), Great Lakes Clinical Trials, LLC d/b/a Flourish Research (Chicago, Illinois, United States, 60640), Tandem Clinical Research GI, LLC (Marrero, Louisiana, United States, 70072), Alliance for Multispecialty Research, LLC (Norman, Oklahoma, United States, 73069), Apex Mobile Clinical Research (Bellaire, Texas, United States, 77401), and Clinical Trials of Texas, LLC dba Flourish Research (San Antonio, Texas, United States, 78229).<br><br>The trial protocol was designed in accordance with the principles established by the International Council for Harmonisation for guidelines on Good Clinical Practice (GCP), the Declaration of Helsinki, and Council for International Organizations of Medical Sciences International Ethical Guidelines. A study monitor was designated by the study sponsor to carefully monitor all aspects of the study for compliance with GCP, standard operating procedures, and applicable government regulations. The study monitor met with the Investigator and staff shortly before the start of the study to review the procedures for study conduct and documentation. During the study, the study monitor visited each site to verify record keeping and adherence to the protocol.

Note that full information on the approval of the study protocol must also be provided in the manuscript.

Field-specific reporting

Please select the one below that is the best fit for your research. If you are not sure, read the appropriate sections before making your selection.

☒ Life sciences ☐ Behavioural & social sciences ☐ Ecological, evolutionary & environmental sciences

For a reference copy of the document with all sections, see nature.com/documents/nr-reporting-summary-flat.pdf

Life sciences study design

All studies must disclose on these points even when the disclosure is negative.

Sample size	Assuming a standard deviation of 4.6 kg, an evaluable sample size of 43 participants per study arm was determined to yield approximately 75% power to detect an effect size of 2 kg for the primary endpoint of lean body mass change from baseline at week 24. Anticipating that approximately 13% would not be evaluable for the primary analysis, a total of 50 participants per arm was planned.<br><br>In total, the EMBRAZE study enrolled 102 participants and was divided into two treatment arms: apitegromab 10 mg/kg + tirzepatide (n=51) or placebo + tirzepatide (n=51). The mean (SD [min, max]) age of patients in the apitegromab arm was 44.2 (11.0 [21, 63]) years; 84.3% (43/51) of participants were female, while 15.7% (8/51) were male. The mean (SD [min, max]) age of patients in the placebo arm was 42.6 (11.5 [18, 64]) years; 80.4% (41/51) of participants were female, while 19.6% (10/51) were male.<br><br>Primary and secondary efficacy analyses at week 24 were conducted in the completer population (all dosed participants who completed treatment and had evaluable lean body mass at week 24 of study [apitegromab arm, n=43; placebo arm, n=44]). Exploratory cardiometabolic

parameters and physical function assessments were also conducted at weeks 24 and 32 for the completer population (apitegromab arm, n=43; placebo arm, n=44). The preservation of treatment effect on lean body mass (apitegromab arm, n=43; placebo arm, n=43), body fat mass (apitegromab arm, n=43; placebo arm, n=43), and body weight (apitegromab arm, n=43; placebo arm, n=42) were also assessed in participants who completed the trial at week 32 of the study.

All safety, apitegromab-related pharmacokinetic, and apitegromab-related pharmacodynamic assessments were conducted for the enrolled participant popultion (apitegromab arm, n=51; placebo arm, n=51). Tirzepatide-related pharmacokinetic assessments were conducted in all participants who received the study drug and had at least one quantifiable result (apitegromab arm, n=51; placebo arm, n=48).

Data exclusions

There were no data exclusions

Replication

The authors are confident in the reproducibility of the findings given a standardized protocol that provided predefined eligibility criteria, dosing regimens, and centralized data collection, was used and strictly followed. The authors confirm that the study is sufficiently powered to demonstrate effect. All subgroup analyses and sensitivity analyses performed were consistent with primary analysis. To provide a broader context, the myostatin biology is well established and apitegromab's mechanism of action is based on selective inhibition of myostatin, with reproducible findings across animal models in multiple species. Importantly, the role of apitegromab to increase muscle mass and function has been demonstrated in 2 separate clinical studies, with the placebo-controlled Phase 3 SAPPHIRE study showing statistically significant and clinically meaningful improvement in motor function versus placebo. Analysis of secondary endpoints as well as all subgroup analyses and sensitivity analyses performed were consistent with primary analysis. Taken together, the authors believe the experimental findings were robust and reproducible.

Randomization

Participants were randomized after the Investigator and Medical Monitor had verified their eligibility. Participants were randomized 1:1 via an interactive web response system (managed by an independent vendor, without blocking or stratification factors) in a double-blind manner to receive apitegromab 10 mg/kg + tirzepatide or placebo + tirzepatide.

Blinding

EMBRAZE was a randomized, double-blind, placebo-controlled, phase 2 trial. The study sponsor, participants, Investigators, and site personnel, with the exception of the pharmacist, will be blinded to treatment assignments. The site pharmacist remained unblinded throughout the duration of the trial; however, if for any reason sponsor personnel needed to be unblinded, the list of personnel and the reason for unblinding was documented. DEXA scans were performed and assessed by blinded personnel, and statistical analyses were planned prior to unblinding at the end of the treatment period.

Reporting for specific materials, systems and methods

We require information from authors about some types of materials, experimental systems and methods used in many studies. Here, indicate whether each material, system or method listed is relevant to your study. If you are not sure if a list item applies to your research, read the appropriate section before selecting a response.

Materials & experimental systems

n/a	Involved in the study	
☐	☒	Antibodies
☒	☐	Eukaryotic cell lines
☒	☐	Palaeontology and archaeology
☒	☐	Animals and other organisms
☐	☒	Clinical data
☒	☐	Dual use research of concern
☒	☐	Plants

Methods

n/a	Involved in the study	
☒	☐	ChIP-seq
☒	☐	Flow cytometry
☒	☐	MRI-based neuroimaging

Antibodies

Antibodies used

Apitegromab is an investigational, fully human monoclonal antibody that selectively binds precursor forms of myostatin to potently inhibit myostatin activation, without activity toward other TGF-β superfamily members.

Validation

Pirruccello-Straub, et al. (Sci Rep, 2018), showed potent myostatin precursor-specific antibody, SRK-015 (apitegromab) bound robustly to proMyostatin and latent myostatin, and no binding could be detected against the TGFβ family members GDF11, Activin A, BMP9 and 10, or TGFβ1.

Clinical data

Policy information about clinical studies

All manuscripts should comply with the ICMJE guidelines for publication of clinical research and a completed CONSORT checklist must be included with all submissions.

Clinical trial registration

ClinicalTrials.gov identifier: NCT06445075

Study protocol

Provided as a supplemental file.

Data collection

From June 18, 2024 through September 17, 2024, participants were enrolled and randomized to receive apitegromab 10 mg/kg

Data collection	every 4 weeks with tirzepatide or placebo every 4 weeks with tirzepatide. Patient data were collected from June 18, 2024 to June 17, 2025 in clinic settings at 7 sites across the United States.

Outcomes

All primary, secondary, and exploratory outcomes were pre-defined in the approved protocol.

Baseline characteristics were collected via standard assessments and a complete physical examination (see protocol for additional details). Demographic information, such as age, sex, race, and ethnicity, were self-reported by eligible participants. Participant sex was not considered in the study design; however, it was used as a covariate in the data analysis as described in the statistical methods section. All participants received standard care counseling with regard to lifestyle recommendations such as diet, physical activity, and behavior modification; however, reporting on these variables was not mandated or monitored.

The primary efficacy endpoint was change from baseline in lean body mass at 24 weeks as assessed by whole body dual-energy X-ray absorptiometry (DEXA; Hologic and GE Lunar) scans in participants receiving apitegromab with tirzepatide compared with those receiving tirzepatide alone. Specifically, each DEXA scan included bilateral arms, bilateral legs, and trunk (including the body organs). Cross-calibration between DEXA systems was not warranted.

Secondary efficacy endpoints included change from baseline in body weight, total fat mass, and DEXA parameters on body composition at week 24. Body weight was obtained via a calibrated scale in a fasted state at each visit from screening through last trial visit. For primary and secondary endpoints based on DEXA measurements, scans were obtained within 7 days prior to participants' initial dose of apitegromab or placebo and were repeated at 24 weeks.

Exploratory efficacy endpoints included change from baseline in DEXA measurements at week 32, physical function (force production with handheld dynamometry and number of repetitions in the chair sit-to-stand test) at weeks 24 and 32, and cardiometabolic markers (eg, hemoglobin A1C [HbA1C]) at weeks 24 and 32.

Prespecified PK and PD endpoints were assessed through the treatment and safety follow-up periods for all participants who received at least 1 dose of apitegromab or placebo. Apitegromab and tirzepatide trough samples were collected every 4 weeks during the treatment period and during safety follow-up visits, and end-of-infusion samples of apitegromab were collected on day 1, week 12, and week 20. Trough concentrations of latent myostatin were collected through 24 weeks and during the safety follow-up period.

Secondary safety endpoints included the frequency of adverse events, clinical laboratory tests, vital signs, electrocardiogram measurements, and physical and psychiatric evaluations reported through last study visit. Data were reviewed throughout the study in a blinded manner by Medical Monitors and the sponsor to ensure participant safety. The presence of serum antidrug antibodies against apitegromab was also assessed. Safety data were collected through week 40 of the study.

Least-squares (LS) means together with 80% confidence intervals (80% CI) for each treatment group and the difference between groups in change from baseline at week 24 in lean body mass were estimated using a linear regression model that controlled controlling for baseline weight, baseline lean body mass, age, and sex. The same regression model was used to estimate LS means and differences between groups with their associated confidence intervals for the changes in other DEXA parameters and the change in weight. For this phase 2 study, all P values are nominal, and no adjustments were made for multiplicity.

Plants

Seed stocks	No seed stocks used.
Novel plant genotypes	No novel plant genotypes produced.
Authentication	No authentication procedures undertaken.
